# Comparative effectiveness of statins for chronic obstructive pulmonary disease patients with pulmonary hypertension: systematic review and network meta-analysis

**DOI:** 10.3389/fmed.2025.1640270

**Published:** 2025-09-02

**Authors:** Guobo Xu, Rui Zhang, Wenrui Huang, Xuelian Du, Weifang Xu

**Affiliations:** ^1^Shenzhen Hospital (Fu Tian) of Guangzhou University of Chinese Medicine, Shenzhen, China; ^2^Shenzhen Traditional Chinese Medicine Hospital, Shenzhen, China

**Keywords:** statins, pulmonary hypertension, chronic obstructive pulmonary disease, network meta-analysis, systematic review

## Abstract

**Introduction and objectives:**

Statins may effectively treat PH-COPD, but current guidelines do not endorse their use. This study aims to assess the comparative effectiveness and safety of Statins in adult patients with pulmonary hypertension associated with chronic obstructive pulmonary disease (PH-COPD) through a systematic review and network meta-analysis.

**Materials and methods:**

We searched 8 databases for randomized controlled trials (RCTs) involving Statins in individuals with PH-COPD from inception to July 1, 2024. We assessed bias using the ROB 2.0 tool and evaluated evidence quality with the CINeMA framework. We employed a Bayesian network meta-analysis approach to assess outcomes including pulmonary artery pressure, exercise tolerance, lung function, oxygenation parameters, inflammatory markers, and vasoactive substances. Using RStudio and other software, we generated forest plots, league tables, and SUCRA curves to evaluate both direct and indirect comparisons.

**Results:**

We analyzed data from 41 RCTs involving 3,606 participants. Our analysis revealed that all 5 statins were effective in reducing Systolic Pulmonary Artery Pressure (sPAP) compared to standard treatment (ST). Rosuvastatin was the most effective, significantly lowering sPAP [MD = –8.8; (95%CI –11.68, −5.85)] and IL-6 (MD = -16.41; 95%Cl − 29.64, −3.04) and improving the 6-Minute Walk Distance (6MWD) (MD = 67.03; 95%Cl 2.77, 130.86). Atorvastatin 20 mg was the most effective in improving lung function, increasing PO2, reducing inflammatory markers such as TNF-*α* and hs-CRP, and lowering ET-1. Finally, Simvastatin 20 mg + ST was identified as the most effective regimen for reducing PCO2 and increasing NO levels.

**Conclusion:**

Our study demonstrates that statins are more effective than standard treatment for adults with PH-COPD. Rosuvastatin is the most effective at reducing sPAP. It also improves the 6MWD and lowers IL-6 levels. Additionally, statins have significantly enhanced lung function, oxygenation parameters, and inflammatory markers in PH-COPD patients, with Atorvastatin showing the best performance in these areas.

**Systematic review registration:**

https://www.crd.york.ac.uk/PROSPERO/view/CRD42024573849, identifier CRD42024573849.

## Introduction

Chronic Obstructive Pulmonary Disease (COPD) is the third leading cause of death globally, following cardiovascular diseases and stroke, posing a significant public health challenge (1, 2). Over the past five decades, the prevalence of COPD has risen markedly, now affecting over 400 million people worldwide (1, 2). According to the World Economic Forum, by 2030, the global cost of COPD treatment will reach $50 trillion annually, surpassing the expenses associated with cardiovascular diseases (2, 3). Pulmonary hypertension (PH) is diagnosed in COPD patients (PH-COPD) when the mean pulmonary artery pressure (mPAP) is ≥25 mmHg. Severe PH-COPD is identified when mPAP is ≥35 mmHg or the cardiac index is below 2.0 L/min/m2.

Approximately half of COPD patients develop PH, which is associated with a poor prognosis. Without timely treatment, the average survival time for these patients is less than 5 years (4, 5). The 2021 Chinese guidelines for diagnosing and treating pulmonary hypertension classify PH-COPD as Group 3 PH (6). Recent studies have focused on targeted therapies for PH, with long-term oxygen therapy currently recommended for Group 3 PH. The 2021 COPD guidelines (7) suggest that treatment for mild to moderate pulmonary hypertension should focus on managing acute COPD exacerbations and improving hypoxemia and hypercapnia rather than using vasodilators or targeted drugs. Long-term oxygen therapy, administered for over 6 months, has improved survival rates in COPD patients and reduced mPAP, likely due to the reduction of hypoxic pulmonary vasoconstriction. However, it has not proven beneficial for patients with a baseline oxygen saturation above 89% (8). Currently, the standard clinical approach for PH-COPD patients involves routine supportive care. This includes maintaining clear airways, facilitating expectoration, using bronchodilators, managing infections, and improving microcirculation. However, searching for safe and effective treatments for these patients remains a critical challenge.

Statins are widely used in clinical settings to lower plasma cholesterol by inhibiting HMG-CoA reductase. Recent studies have revealed that statins also offer benefits beyond lipid reduction, such as reducing inflammation, stabilizing endothelial cells, preventing pulmonary vascular remodeling, and reducing lipid oxidation (9–16). Additionally, studies have suggested that statins may effectively treat PH-COPD, opening up new treatment possibilities (17). However, current guidelines do not endorse their use for this condition due to small sample sizes and a lack of direct comparisons among different statins. Furthermore, there is no comprehensive analysis comparing the efficacy of various statins. Therefore, further research and network meta-analyses are necessary to assess their potential benefits fully.

## Materials and methods

This study follows the PRISMA 2020 guidelines and the PRISMA-NMA extension for network meta-analyses (18, 19). It has been registered with PROSPERO (CRD42024573849), as outlined in Appendix 1.

### Search strategy

We conducted a comprehensive search across multiple databases, including CKNI, Wanfang, Weipu, CBM, PubMed, Embase, Web of Science, and CENTRAL, to find randomized controlled trials (RCTs) on statins for PH-COPD patients, covering each database from inception to July 1, 2024. We also manually searched ClinicalTrials.gov and reviewed references from selected articles and related systematic reviews. Two reviewers independently selected the studies, resolving discrepancies with a third reviewer. The full search strategy is detailed in Appendix 2.

### Eligibility criteria

Eligible RCTs focused on PH-COPD, including cases with cor pulmonale. Trials assessed any commercially available statin against a placebo, standard treatment (STs), or both in the control group. Control groups could include consistent additional medications, typically oxygen therapy, bronchodilators, expectorants, and antibiotics. Statin interventions had to last more than 4 weeks. Only RCTs published in peer-reviewed journals were included, excluding conference abstracts, duplicates, crossover designs, and non-English or non-Chinese publications.

### Screening process

We imported the retrieved items from the databases into EndNote 20, eliminated duplicates, and integrated them with results from additional sources. The screening process comprised three stages. Initially, two reviewers independently assessed the articles based on their titles, including those with uncertain relevance. The selected articles were summarized in the second stage, and any disagreements were addressed through discussion and consultation with a third reviewer. In the final stage, articles with appropriate titles and abstracts were meticulously reviewed against the established inclusion and exclusion criteria.

### Data extraction

For each eligible study, we systematically collected data using a pre-designed template, including the title, first author, publication date, study location, and methodology (randomization, blinding, allocation concealment, outcome data completeness, selective reporting). We also gathered demographic details (age, sample sizes, sex ratios, inclusion/exclusion criteria, COPD stages, baseline sPAP and intervention specifics; type of statin, dosage, treatment duration, control group interventions).

Our evaluation focused on six outcomes: pulmonary artery pressure, exercise tolerance, lung function, oxygenation parameters, inflammatory markers, and vasoactive substances. Pulmonary artery pressure (sPAP, mPAP), exercise tolerance (6-Minute Walk Distance (6MWD)), lung function (FVC, FEV1, FEV1/FVC), and Oxygenation Parameters (PO2, PCO2) were treated as primary outcomes, while inflammatory markers (TNF-a, hs-CRP, IL-6) and vasoactive substances (NO, ET-1) were secondary outcomes. We assessed the safety of interventions by reviewing the incidence of adverse events. Two reviewers conducted Data extraction independently, with any discrepancies resolved through consultation with a third reviewer.

### Quality assessment of evidence

We assessed the risk of bias in the included trials using the Cochrane Risk of Bias tool (RoB 2.0) (20). This tool evaluates five key areas: randomization process, adherence to intended interventions, management of missing data, consistency of outcome measurements, and reporting of pre-specified outcomes. Proper procedures in these domains indicate a low risk of bias, while issues suggest a high risk. Two reviewers independently conducted the bias assessment, resolving discrepancies by consensus. We used the CINeMA (Confidence in Network Meta-Analysis) framework to evaluate the quality of evidence from our network meta-analysis. CINeMA examines the certainty of evidence across six domains: within-study bias, reporting bias, indirectness, imprecision, heterogeneity, and inconsistency. Each domain was meticulously assessed to ensure a thorough evaluation of the evidence (21, 23).

### Methods for evidence synthesis

This network meta-analysis was conducted in RStudio using a Bayesian framework to integrate direct and indirect evidence, thereby enabling a comprehensive comparison and ranking of multiple treatments. We assessed transitivity by comparing clinical and methodological variables between studies providing direct and indirect evidence (24). Consistency within closed loops and the entire network was evaluated using node-splitting and design treatment interaction models (24–26). For both continuous and dichotomous outcomes, we employed random-effects models to calculate mean differences (MD) with 95% confidence intervals (CIs). We assessed heterogeneity using the *I*^2^ statistic, with values above 50% indicating significant heterogeneit (27). Indirect comparisons employed Bayesian network meta-analysis with Markov chain Monte Carlo (MCMC) methods, involving 20,000 burn-ins and 50,000 iterations with a thinning interval of 10. Summary estimates for pairwise comparisons were derived, and treatment effects were ranked using Surface Under the Cumulative Ranking Curve (SUCRA) values based on posterior probabilities (28).

## Results

### Literature selection and study characteristics

**Figure 1:**
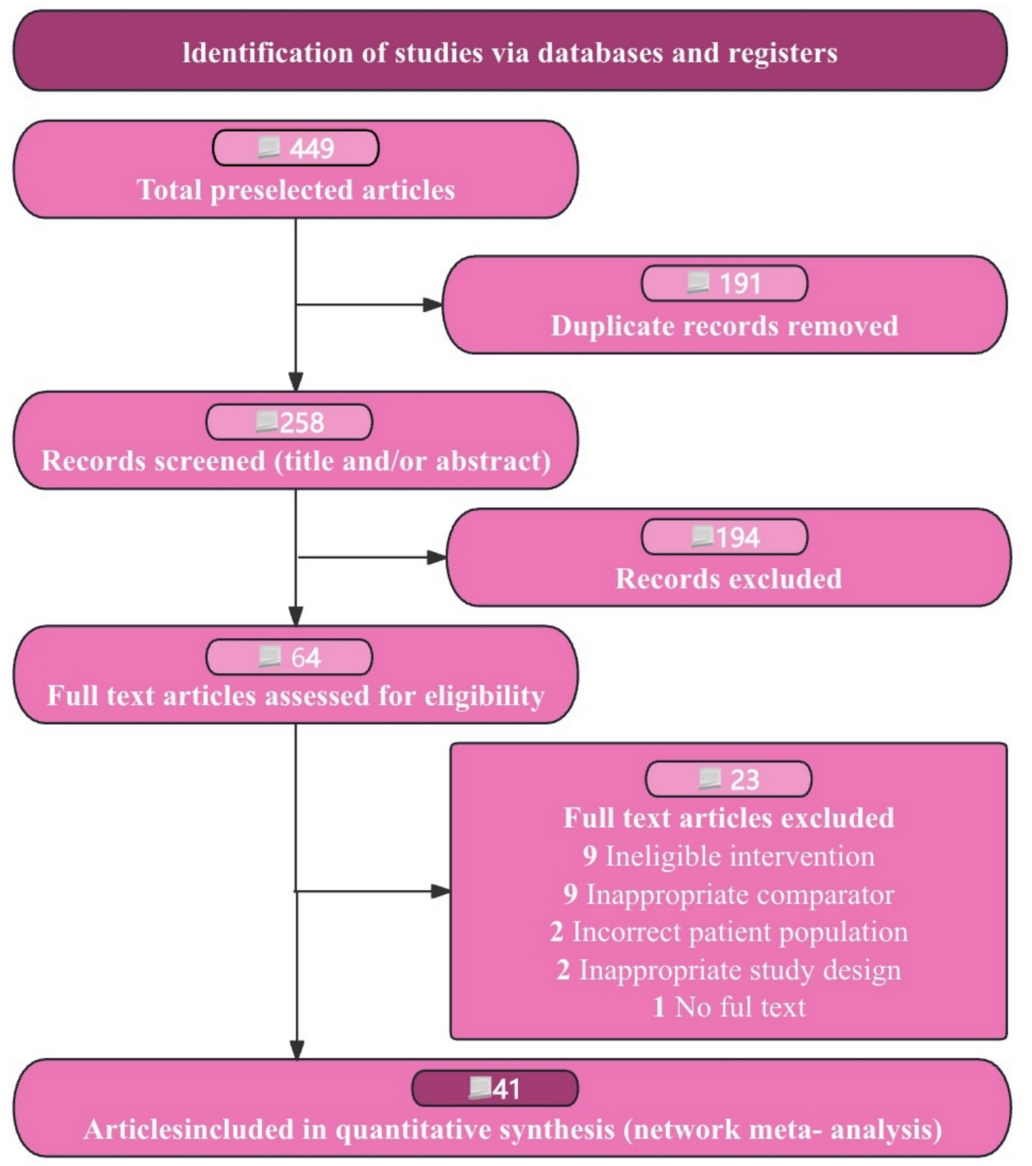
Flow diagram of preferred reporting items identified, included, and excluded for systematic reviews and meta-analyses (PRISMA).

We began with 449 records from our database search. After removing 191 duplicates, we reviewed 258 titles and abstracts, discarding 194 records. This left us with 64 full-text articles for a detailed assessment. Following our inclusion criteria, we identified 41 RCTs involving 3,606 patients as suitable for our study (see Figure 1). These trials were conducted in three countries, with sample sizes ranging from 16 to 80 participants and intervention durations between 1 and 12 months (see Table 1). From the publications reviewed, we identified five statin medications. Our network analysis then compared these five statins, all approved by regulatory authorities (see Appendix 3: Table S3.1).

**Table 1:** Baseline of characteristics of included studies.

Study	Country	Design	Stages of COPD	Gender ratio	Age years	PH/sPAP	Follow-up duration	Number of participants	Randomized treatments	Dose and frequency
Cao 2018 (21)	China	RCT	NA	17: 13	70.46 ± 5.02	57.69 ± 8.02	6 months	60	Atorvastatin + ST:30	20 mg QD
				17: 13	71.03 ± 4.91	56.79 ± 7.64			ST:30	
Qu 2012 (38)	China	RCT	NA	42: 8	70.52 ± 9.63	58.33 ± 8.95	6 months	100	Atorvastatin + ST:50	10 mg QD
				44: 6	71.29 ± 11.1	56.04 ± 8.51			ST:50	
He 2019 (39)	China	RCT	NA	25: 18	68.52 ± 6.21	NA	3 months	86	Atorvastatin + ST:43	20 mg QD
				27: 16	69.48 ± 5.52				ST:43	
Yu 2012 (40)	China	RCT	NA	57: 21	65.154 ± 6.6	50.3 ± 8.4	12 months	156	Atorvastatin + ST:78	10 mg QD
				61: 17	64.895 ± 6.7	51.1 ± 8.2			ST:78	
Luo 2013 (41)	China	RCT	NA	21: 9	72 ± 11	32.2 ± 6.4	3 months	60	Atorvastatin + ST:30	20 mg QD
				20: 10	73 ± 10	31.3 ± 5.8			ST:30	
Wang 2015 (42)	China	RCT	Acute period	27: 12	66.4 ± 6.3	56.46 ± 4.23	3 months	78	Atorvastatin + ST:39	20 mg QD
				29: 10	66.0 ± 6.1	55.68 ± 7.70			ST:39	
Yan 2018 (43)	China	RCT	NA	21: 11	64.9 ± 4.3	52.78 ± 5.42	6 months	64	Atorvastatin + ST:32	20 mg QD
				19: 13	63.3 ± 3.9	53.12 ± 5.67			ST:32	
Mao 2017 (11)	China	RCT	NA	32: 21	51.28 ± 3.12	>36	3 months	106	Atorvastatin + ST:53	20 mg QD
				30: 23	52.42 ± 3.29				ST:53	
Zhang 2019 (44)	China	RCT	NA	32: 28	46.3 ± 15.1	63.37 ± 23.27	6 months	120	Atorvastatin + ST:60	20 mg QD
				29: 31	45.6 ± 14.9	62.43 ± 22.95			ST:60	
Zhang 2013 (45)	China	RCT	NA	NA	52.41 ± 7.75	>36	6 months	98	Atorvastatin + ST:49	20 mg QD
					51.47 ± 7.63				ST:49	
Wu 2014 (46)	China	RCT	NA	NA	NA	NA	1 months	90	Atorvastatin + ST:30	20 mg QD
									Atorvastatin + ST:30	10 mg QD
									ST:30	
Deng 2015 (47)	China	RCT	NA	25: 15	63.13 ± 3.1	>30	12 months	80	Atorvastatin + ST:40	10 mg QD
				24: 16	62.33 ± 3.1				ST:40	
Liu 2016 (48)	China	RCT	NA	19: 21	71.07 ± 6.78	NA	1 months	80	Atorvastatin + ST:40	20 mg QD
				23: 17	70.07 ± 8.78				ST:40	
Li 2012 (49)	China	RCT	NA	NA	NA	NA	2 months	70	Atorvastatin + ST:35	10 mg QD
									ST:35	
Sun 2020 (13)	China	RCT	NA	29: 21	56.5 ± 3.3	NA	3 months	100	Atorvastatin + ST:35	20 mg QD
				27: 23	57.2 ± 4.4				ST:35	
Chen 2016 (50)	China	RCT	stable period	30: 5	66 ± 4.0	50.2 ± 8.6	6 months	76	Atorvastatin + ST:35	20 mg QD
				37: 4	67 ± 4.8	51.8 ± 6.2			ST:41	
Jiang 2015 (51)	China	RCT	NA	38: 26	58.9 ± 8.7	52.3 ± 7.6	3 months	128	Atorvastatin + ST:35	20 mg QD
				40: 24	58.7 ± 8.5	51.2 ± 7.9			ST:41	
Niu 2015 (10)	China	RCT	NA	26: 24	45.34 ± 14.46	52.26 ± 12.16	6 months	100	Atorvastatin + ST:50	20 mg QD
				27: 23	45.22 ± 14.29	51.32 ± 11.84			ST:50	
Wang 2011 (52)	China	RCT	stable period	24: 11	64 ± 3.5	53.2 ± 4.8	6 months	70	Fluvastatin + ST:35	40 mg QD
				22: 13	63 ± 5.2	52.8 ± 4.6			ST:35	
Wang 2012 (53)	China	RCT	stable period	32: 24	62.4 ± 7.3	48.9 ± 8.4	6 months	112	Fluvastatin + ST:56	40 mg QD
				33: 23	68.4 ± 8.5	48.2 ± 7.6			ST:56	
Xu 2020 (22)	China	RCT	Acute period	28: 24	65.42 ± 6.14	52.19 ± 6.18	6 months	108	Rosuvastatin + ST:52	10 mg QD
				30: 26	65.31 ± 6.23	52.04 ± 6.23			ST:56	
Tang 2018 (54)	China	RCT	Acute period	14: 16	69.84 ± 7.07	52.08 ± 11.7	6 months	60	Rosuvastatin + ST:30	10 mg QD
				13: 17	68.12 ± 7.19	50.41 ± 10.6			ST:30	
Ren 2018 (55)	China	RCT	stable period	38: 32	57.42 ± 11.31	68.63 ± 8.26	4 months	140	Rosuvastatin + ST:70	10 mg QD
				39: 31	57.23 ± 11.29	68.39 ± 8.52			ST:70	
Nan 2016 (56)	China	RCT	stable period	NA	NA	55 ± 7	3 months	60	Rosuvastatin + ST:70	20 mg QD
						57 ± 6			ST:70	
Ye 2015 (57)	China	RCT	stable period	23: 7	58.5 ± 7.9	37.91 ± 4.36	1 months	60	Simvastatin + ST:30	20 mg QD
				25: 5	59.4 ± 6.8	37.98 ± 4.45			ST:30	
Rang 2013 (58)	China	RCT	NA	17: 13	73 ± 10	36.2 ± 0.8	3 months	60	Simvastatin + ST:30	40 mg QD
				18: 12	72 ± 8	36.3 ± 0.8			ST:30	
Xia 2013 (59)	China	RCT	NA	84: 16	69.0 ± 8.0	55.3 ± 8.6	2 months	100	Simvastatin + ST:50	40 mg QD
						54.7 ± 7.3			ST:50	
Chen 2017 (60)	China	RCT	stable period	25: 25	63.35 ± 4.26	31.73 ± 4.80	1 months	100	Simvastatin + ST:50	20 mg QD
				28: 22	63.20 ± 4.89	32.76 ± 4.83			ST:50	
Ding 2016 (61)	China	RCT	NA	28: 22	70.29 ± 7.86	65.46 ± 5.81	6 months	100	Simvastatin + ST:50	20 mg QD
				27: 23	70.15 ± 7.62	65.35 ± 5.87			ST:50	
Tang 2017 (62)	China	RCT	NA	26: 17	61.7 ± 9.4	65.15 ± 5.12	6 months	86	Simvastatin + ST:43	20 mg QD
				28: 15	60.8 ± 8.9	64.74 ± 4.86			ST:43	
Zhang 2015 (63)	China	RCT	stable period	31: 14	69.88 ± 6.84	>40	6 months	90	Simvastatin + ST:45	20 mg QD
				33: 12	65.48 ± 6.13				ST:45	
Hu 2019 (64)	China	RCT	stable period	32: 23	62.9 ± 5.8	56.19 ± 6.26	3 months	110	Simvastatin + ST:55	20 mg QD
				30: 25	63.3 ± 6.0	56.14 ± 6.29			ST:55	
Tong 2016 (65)	China	RCT	stable period	40: 25	64.5 ± 8.5	37.15 ± 4.23	3 months	130	Simvastatin + ST:65	20 mg QD
				41: 24	64.2 ± 8.6	36.25 ± 4.42			ST:65	
Sun 2014 (66)	China	RCT	NA	24: 16	67.8	55.25 ± 8.01	3 months	80	Simvastatin + ST:40	20 mg QD
				26: 14	68.2	56.41 ± 7.54			ST:40	
Yan 2012 (67)	China	RCT	Acute period	31: 21	68 ± 10	35.5 ± 0.3	1 months	104	Simvastatin + ST:52	40 mg QD
				29: 23	67 ± 9	35.5 ± 0.4			ST:52	
Liu 2010 (68)	China	RCT	stable period	52: 10	67 ± 4.0	51.1 ± 8.2	6 months	62	Simvastatin + ST:52	20 mg QD
						50.3 ± 8.4			ST:52	
Arian 2017 (9)	Iran	RCT	NA	10: 6	65.8 ± 11.5	47.9 ± 15.4	6 months	34	Atorvastatin + ST:16	40 mg QD
				13: 6	63.7 ± 7.6	49.2 ± 16.3			ST:18	
Chogtu 2016 (16)	India	RCT	stable period	NA	61.4 ± 8.4	NA	3 months	62	Rosuvastatin + ST:32	10 mg QD
					65.9 ± 9.7				ST:30	
Lee 2009 (69)	China	RCT	NA	20: 7	71 ± 8	47 ± 8	6 months	53	Pravastatin: 27	40 mg QD
				19: 7	72 ± 6	47 ± 7			ST: 26	
Liu 2013 (70)	China	RCT	NA	20: 13	66.2 ± 7.4	52.7 ± 8.1	6 months	68	Atorvastatin + ST:33	20 mg QD
				23: 12	64.9 ± 8.2	51.7 ± 7.9			ST:35	
Moosavi 2013 (4)	Iran	RCT	Acute period	15: 9	65.0 ± 11.0	48.5 ± 6.9	6 months	33	Atorvastatin + ST:19	20 mg QD
				13: 8	68.0 ± 14.0	49.7 ± 1.4			ST:17	

PH, Pulmonary Hypertension; sPAP, Systolic Pulmonary Arterial Pressure; RCT, randomized controlled trial; NA, none report; ST, standard treatment; QD, once daily.

### Risk of bias, certainty of evidence, and consistency

Appendix 4 provides an overview of the risk of bias for each trial. A major issue was the insufficient details regarding blinding methods for participants, researchers, and assessors, as well as the absence of data loss reports. Of the 41 trials reviewed, the summaries of those with a low risk of bias are: 37 studies (90.2%) for randomization, 35 studies (85.3%) for adherence to interventions, 38 studies (92.6%) for missing outcome data, 40 studies (97.5%) for outcome measurement, and 38 studies (92.6%) for reporting results. Overall, 5 studies (12.1%) exhibited a high risk of bias, and 2 (4.8%) raised concerns about potential bias.

Our evaluation of the consistency between direct and indirect evidence shows high agreement across all comparisons, illustrated in density and convergence plots (Appendices 6, 7). The *I*^2^ results indicate no significant heterogeneity within the network, with most comparisons showing low heterogeneity (Appendix 5). Using CINeMA, we found most pairwise comparisons had low confidence levels, with a few showing moderate to high confidence (Appendix 8). All networks complied with the transitivity principle, ensuring the validity of indirect comparisons (Appendix 8: Table S8.1). Additionally, the funnel plots showed no asymmetry (Appendix 9). This comprehensive analysis underscores the robustness and reliability of our findings.

### Pulmonary artery pressure

**Figure 2:**
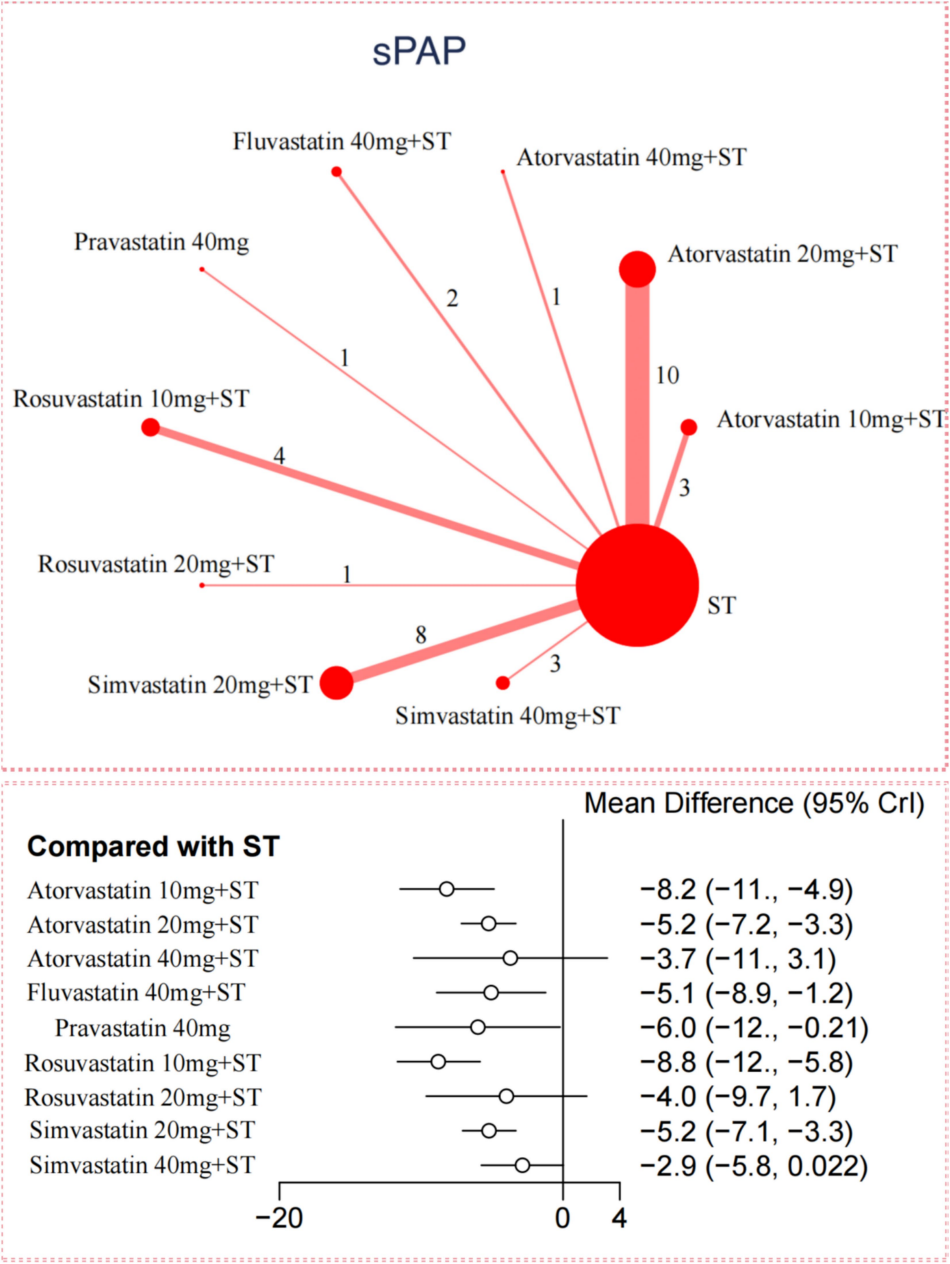
Network and forest plot of available comparisons of Statins for sPAP.

For sPAP reduction, the network meta-analysis included 33 trials with 2,816 participants. Compared to ST, all 5 statins significantly reduced sPAP levels in adults with PH-COPD (Figure 2). Rosuvastatin 10 mg combined with ST showed the most substantial reduction in sPAP [MD = –8.8; (95%CI –11.68 to −5.85); SUCRA 91.5%; high-confidence evidence], followed by Atorvastatin 10 mg with ST [MD = –8.21; (95%CI –11.49 to −4.87); SUCRA 85.8%; high-confidence evidence], and Pravastatin 40 mg [MD = –6.01; (95%CI –11.81 to −0.21); SUCRA 61%; low-confidence evidence]. Among the different statins, Rosuvastatin 10 mg combined with ST resulted in a significantly more significant reduction in sPAP compared to Atorvastatin 20 mg with ST, Simvastatin 20 mg with ST, and Simvastatin 40 mg with ST (Appendix 12: Table S12.1). According to CINeMA, the overall quality of evidence for sPAP was mainly moderate to high (Appendix 8: Table S8.2).

**Figure 3:**
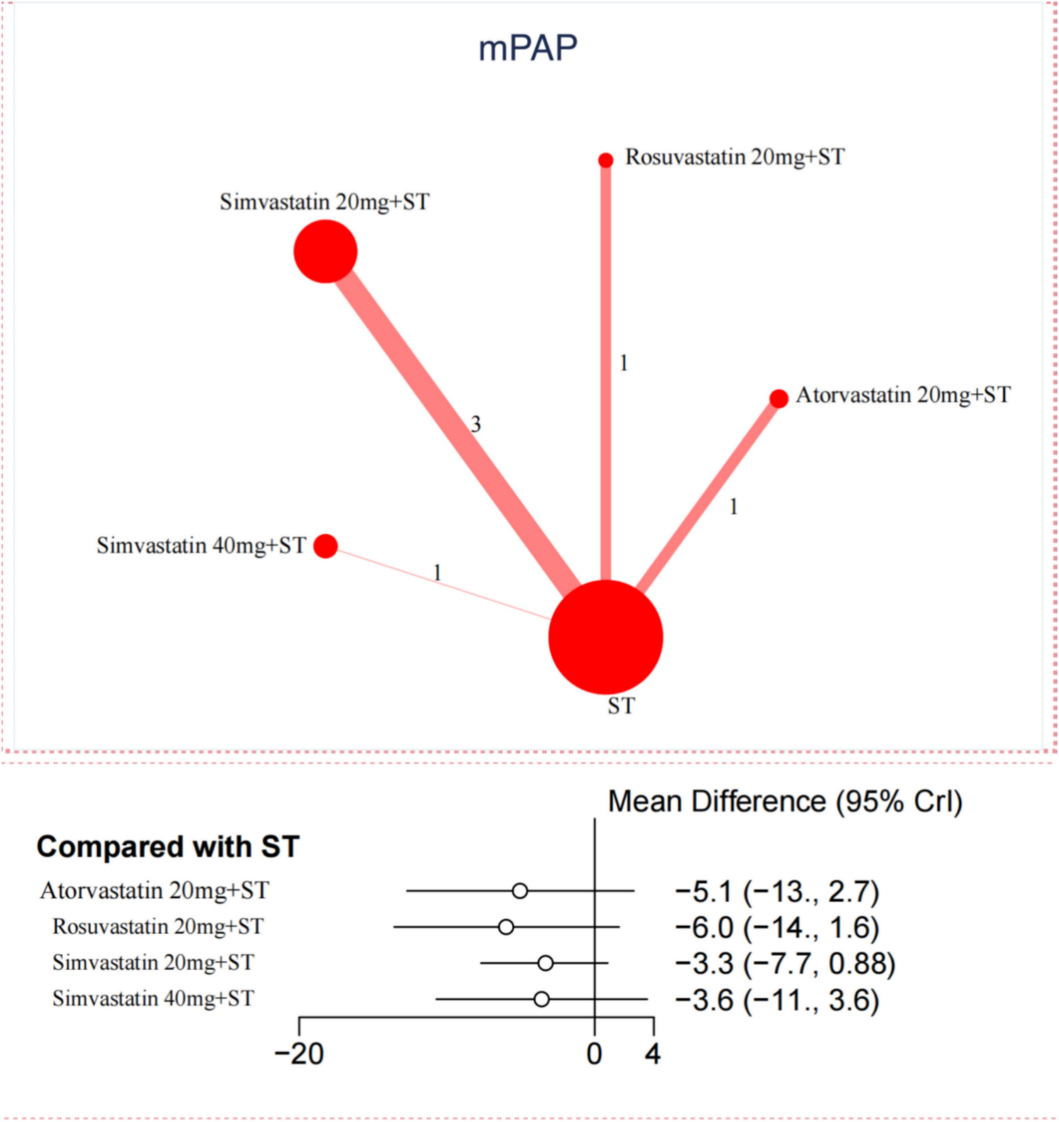
Network and forest plot of available comparisons of Statins for mPAP.

In contrast, for the reduction of mPAP, only 6 trials were included in the network meta-analysis. No significant differences were observed between statins and ST in reducing mPAP (Figure 3). Further details are provided in Appendix Figures S11.1, S11.2 and Appendix Tables S12.1, S12.2.

### Exercise tolerance

Based on the 6MWD assessment, a network meta-analysis was conducted, incorporating 12 RCTs with 1,119 participants. This analysis confirmed the effectiveness of all 5 different doses of statins in comparing ST (Appendix 10: Supplementary Figure S10.1). Among these, Rosuvastatin 20 mg combined with ST was the most effective in enhancing 6MWD, with a MD of 67.03 (95%Cl 2.77 to 130.86) and a SUCRA of 87.9%. A detailed comparison of 6MWD results is provided in Appendix 11: Table S11.3 and Appendix 12: Table S12.3.

### Lung function

The network meta-analysis examined the effects of statins on lung function through 21 studies each for FVC and FEV1. FVC studies involved 1,976 participants, while FEV1 studies included 1,868 participants. Eleven studies with 1,038 participants were also analyzed for the FEV1/FVC ratio.

Atorvastatin 20 mg combined with ST was found to be the most effective statin for improving FVC [MD = 0.4; (95%Cl 0.21 to 0.58); SUCRA 82.2%] (Appendix 10: Figure S10.2). Rosuvastatin 10 mg + ST and Simvastatin 20 mg + ST also significantly improved FVC compared to ST alone. In terms of enhancing FEV1, all 4 different doses of statins were effective. Pravastatin 40 mg led to the most substantial increase in FEV1 (MD = 0.56 (95%CI 0.27 to 0.85); SUCRA 98.7%). This was followed by Rosuvastatin 10 mg + ST (MD = 0.33; (95% CI 0.16 to 0.5); SUCRA 79.5%) and Atorvastatin 20 mg + ST (MD = 0.17; (95% CI 0.06 to 0.3); SUCRA 50.9%). For improving the FEV1/FVC ratio, Atorvastatin 20 mg + ST, Rosuvastatin 10 mg + ST, and Simvastatin 20 mg + ST all showed significant benefits over ST alone. The SUCRA data (Appendix 11: Tables S11.4–S11.6) and additional tables (Appendix 12: Table S12.4–S12.6) provide detailed comparisons of these outcomes.

### Oxygenation parameters

The effect of statins on oxygenation parameters was assessed through measurements of PO2 and PCO2. The network meta-analysis revealed that Atorvastatin 20 mg + ST (MD = 11.81; 95%Cl 2.93 to 20.78), Atorvastatin 10 mg + ST (MD = 11.6; 95%Cl 2.78 to 20.43), and Simvastatin 20 mg + ST (MD = 7.51; 95%Cl 3.71 to 11.49) all led to significant increases in PO2 levels compared to ST alone. Only Simvastatin 20 mg + ST was significantly lower PCO2, with a MD of −9.59 (95%Cl − 16.65 to −2.5) (see Appendix Figures S10.5, S10.6, S11.7, S11.8 and Appendix Tables S11.7, S11.8, S12.7, S12.8).

### Inflammatory markers

The effect of statins on inflammation was evaluated by measuring TNF-*α*, hs-CRP, and IL-6 levels. The network meta-analysis demonstrated that Atorvastatin 20 mg + ST and Simvastatin 20 mg + ST were effective in significantly reducing TNF-α, hs-CRP, and IL-6 compared to ST alone. Among the statins studied, Rosuvastatin 10 mg + ST was found to be the most effective in lowering IL-6, with a MD of −16.41 (95%Cl − 29.64 to −3.04) (refer to Supplementary Figures S10.7–S10.9 and Supplementary Tables S11.9–S11.11, S12.9–S12.11.

### Vasoactive substances

The network meta-analysis assessed the effects of 5 different doses of statins on NO and ET-1 levels. The findings showed that Simvastatin 20 mg + ST, Atorvastatin 10 mg + ST, and Atorvastatin 20 mg + ST were significantly more effective than ST alone in increasing NO and decreasing ET-1. Specifically, Simvastatin 20 mg + ST was the most effective in raising NO levels, with a MD of 8.42 (95%Cl 3.66 to 12.86). Meanwhile, Atorvastatin 20 mg + ST was the most effective in lowering ET-1, with a MD of −9.82 (95%Cl − 13.03 to −6.6) (refer to Supplementary Figures S10.10, S10.11 and Supplementary Tables S11.12, S11.13, S12.12, S12.13).

### Adverse events

Fourteen studies involving 947 patients monitored for adverse reactions during treatment. Of these, 7 studies reported no adverse effects, and 1 study noted that a few patients experienced nausea and vomiting. The remaining 6 studies detailed specific adverse reactions. All reported adverse events occurred in the Atorvastatin treatment groups. These included 4 cases of elevated liver enzymes, all at a dosage of 20 mg, which returned to normal after dose reduction. Additionally, 3 cases of upper abdominal discomfort did not affect the continuation of treatment, 4 cases of muscle pain, and 3 cases of gastrointestinal issues. Specific management measures for these reactions were not provided. Due to the limited data, a network meta-analysis could not be conducted for these adverse effects.

### Sensitivity analyses and meta-regressions

To test the robustness of our results, we performed a sensitivity analysis by excluding one study at a time from each group. No single study was found to affect the outcomes significantly. As shown in Appendix 13, the sensitivity analysis results were consistent with the primary findings, confirming their robustness. We also conducted a meta-regression analysis to evaluate the impact of potential baseline effect modifiers on the primary outcomes. Factors such as the baseline sPAP, gender, and age were assessed. None of these factors significantly influenced the primary outcomes (Appendix 14).

## Discussion

### Principal findings

This network meta-analysis thoroughly assesses the efficacy and safety of 9 different statin doses for treating adults with PH-COPD. The statins evaluated include Atorvastatin, Fluvastatin, Pravastatin, Rosuvastatin, and Simvastatin. We analyzed data from 41 RCTs involving 3,606 participants, focusing on key outcomes such as pulmonary artery pressure, exercise tolerance, lung function, oxygenation parameters, inflammatory markers, and vasoactive substances. Our analysis revealed that all 5 statins were effective in reducing sPAP compared to ST. Rosuvastatin was the most effective, significantly lowering sPAP and IL-6 and improving the 6MWD. Rosuvastatin was particularly effective at 10 mg for the first two outcomes and at 20 mg for enhancing 6MWD. Atorvastatin at 20 mg was the most effective in improving lung function, increasing PO2, reducing inflammatory markers such as TNF-*α* and hs-CRP, and lowering ET-1. Although Pravastatin at 40 mg had the highest SUCRA score for improving FEV1, its findings were based on a single study, which limits its reliability. Finally, Simvastatin 20 mg + ST was identified as the most effective regimen for reducing PCO2 and increasing NO levels. A comprehensive summary of effect estimates for key outcomes, along with their corresponding CINeMA confidence ratings, is presented in Appendix 15 to facilitate interpretation and support clinical decision-making.

### Comparisons with other studies

Earlier pairwise meta-analyses in COPD, sometimes including mixed study designs, suggested a modest reduction in pulmonary arterial pressure (PAP) with statins but without drug–dose differentiation. For example, Lu et al. (29) reported that statins reduced PH in COPD (SMD ≈ −0.71) without robust head-to-head ranking, while Wang et al. found sPAP decreased by ~4–5 mmHg versus placebo. Our NMA corroborates a class effect on sPAP and further ranks rosuvastatin as the most effective, with supportive RCT signals in PH-COPD cohorts (30). For exercise tolerance, prior meta-analyses in pulmonary vascular disease showed inconsistent 6MWD gains, particularly null in PAH-focused analyses (31, 32), whereas COPD-focused reviews suggested potential improvement (33). Our NMA aligns with the COPD signal and indicates rosuvastatin 20 mg confers the greatest 6MWD improvement, consistent with small RCTs/series reporting class-wide benefits in COPD-PH. By integrating more COPD-PH RCTs and modeling dose, we likely captured efficacy diluted in disease-agnostic analyses.

Beyond hemodynamics and exercise capacity, our NMA identifies drug–dose patterns across lung function, oxygenation, and inflammatory/vasoactive biomarkers. Atorvastatin 20 mg ranked highest for FEV₁, PO₂, PCO₂ reduction, TNF-*α*, hs-CRP, and ET-1 improvement; rosuvastatin 10 mg was optimal for IL-6 reduction and sPAP lowering, while 20 mg was needed for maximal 6MWD gain. Simvastatin 20 mg plus ST ranked best for NO increase. These findings expand on prior syntheses (33, 34) by separating drug–dose nodes and are biologically plausible given statins’ pleiotropy (35). The pravastatin 40 mg FEV₁ signal carried wide uncertainty (single study), consistent with the limited direct evidence base. Our dose–response modeling suggests rosuvastatin 10 mg is sufficient for sPAP and IL-6, whereas 20 mg may be required for exercise capacity; atorvastatin 20 mg appears optimal for composite pulmonary/inflammatory endpoints. These results complement mechanistic and observational studies supporting a protective association of statins against PH in COPD, while CINeMA ratings contextualize confidence across nodes (36, 37).

By focusing on PH-COPD and disentangling dose-specific effects, our work addresses limitations of earlier reviews that mixed etiologies, lacked dose resolution, or were underpowered for ranking. Compared with Lu et al.’s COPD-focused NMA (29), we incorporated more PH-COPD RCTs, modeled drug–dose networks across hemodynamic, functional, gas-exchange, inflammatory, and vasoactive outcomes, and applied CINeMA to guide clinical selection (e.g., rosuvastatin for hemodynamics/6MWD; atorvastatin for lung function/inflammation/ET-1). Clinically, our rankings suggest: rosuvastatin (10 mg for hemodynamics/IL-6; 20 mg for 6MWD) when prioritizing vascular and performance outcomes; atorvastatin 20 mg when lung function, oxygenation, and systemic inflammation are key targets; and simvastatin 20 mg when aiming to reduce PCO₂ and augment NO. The absence of new safety signals is consistent with prior COPD meta-analyses (34), though higher-dose nodes warrant future monitoring for myalgias and transaminase elevations.

### Policy implications

This study evaluates the effectiveness of statins in treating adult patients with PH-COPD. Among the statins, Rosuvastatin was the most effective in reducing sPAP and improving 6MWD, especially at higher doses. Atorvastatin showed the greatest improvement in lung function. Statins also demonstrated anti-inflammatory effects, with notable efficacy from Atorvastatin 20 mg and Rosuvastatin 10 mg. Additionally, combining statins with standard treatments significantly improved PO2 and PCO2 levels compared to standard treatments alone. Unlike some targeted therapies, statins effectively reduce pulmonary artery pressure without compromising oxygen saturation, offering a promising approach for managing COPD. The potential benefits of statins in PH may be attributed to their pleiotropic effects beyond lipid-lowering. Statins can improve endothelial function by enhancing NO bioavailability, reducing oxidative stress, and inhibiting the expression of ET-1, a potent vasoconstrictor. They also exert anti-inflammatory effects by suppressing pro-inflammatory cytokines such as TNF-*α* and IL-6, which are implicated in pulmonary vascular remodeling. These mechanisms may collectively contribute to reduced pulmonary artery pressure and improved exercise capacity in PH-COPD patients.

While these pleiotropic mechanisms provide a biologically plausible explanation for the observed clinical benefits, they remain incompletely understood in the context of PH-COPD. Much of the mechanistic evidence is derived from preclinical studies or extrapolated from other disease models, such as atherosclerosis or left heart failure. It is still unclear to what extent individual statins differ in their endothelial, anti-inflammatory, or vasomodulatory effects, especially in patients with coexisting pulmonary and systemic vascular disease. Moreover, the relative contributions of lipid-independent versus lipid-lowering pathways remain debated. A more precise understanding of these mechanisms will require mechanistic studies directly targeting the pulmonary vasculature in COPD-related PH, ideally in human subjects.

However, given that most of the included studies were conducted in Chinese populations, caution is needed when applying these findings to other ethnic and geographic groups. Differences in genetic background, comorbidities, environmental exposures, and healthcare systems may influence treatment response. Future studies in more diverse populations are warranted to validate the generalizability and optimize the clinical application of statins in PH-COPD globally.

### Strengths and limitations of this study

This study is the most comprehensive and current systematic review and network meta-analysis of statins for patients with PH-COPD. It compares the effectiveness of nearly all available statins in reducing pulmonary artery pressure and evaluates their impact on exercise tolerance, lung function, and blood gas levels (PO2, PCO2). The study also examines effects on inflammatory markers (TNF-*α*, hs-CRP, IL-6) and vasoactive substances (NO, ET-1), using the CINeMA quality assessment method to ensure result reliability.

Despite its strengths, this study has several limitations. Most included trials were conducted in China and published in Chinese, which may limit the generalizability of the findings to other populations and ethnic groups. Although five statins were analyzed, some (e.g., pravastatin and fluvastatin) were supported by only a small number of studies, reducing the reliability of efficacy estimates for these agents. Safety reporting was also inconsistent, with adverse events described in only 14 studies, mostly involving atorvastatin, making it difficult to draw firm conclusions regarding tolerability. Furthermore, some outcomes—especially secondary ones—were associated with wide confidence intervals and low certainty ratings according to the CINeMA assessment. Several studies carried a moderate risk of bias due to insufficient reporting of randomization or blinding procedures. Most trials relied on echocardiographic measurements rather than right heart catheterization, which may have compromised the precision of pulmonary pressure estimates.

These limitations highlight the need for future high-quality, multicenter trials conducted in diverse geographic and ethnic populations, with standardized outcome reporting, rigorous methodology, and adequate safety monitoring. Greater emphasis on underrepresented statins and the use of gold-standard diagnostic tools such as RHC will also be essential for strengthening the evidence base and informing clinical decision-making in PH-COPD management.

## Conclusion

Our study demonstrates that statins are more effective than standard treatment for adults with PH-COPD. Among the statins, Rosuvastatin is the most effective at reducing sPAP. It also improves the 6MWD and lowers IL-6 levels. Additionally, statins have significantly enhanced lung function, oxygenation parameters, and inflammatory markers in PH-COPD patients, with Atorvastatin showing the best performance in these areas. Further research with larger sample sizes and higher quality is needed to confirm these findings and evaluate the safety of different statins.

## Data Availability

The raw data supporting the conclusions of this article will be made available by the authors, without undue reservation.
