# Supplementary material for: Comparative effectiveness of statins for chronic obstructive pulmonary disease patients with pulmonary hypertension: systematic review and network meta-analysis
Source: Front Med (Lausanne). 2025 Sep 2;12:1640270. doi: 10.3389/fmed.2025.1640270 (PMC12436114; doi:10.3389/fmed.2025.1640270)
Supplement: Supplementary file 1 [file Data_Sheet_1.docx]

**Supplementary Appendix**

**Comparative effectiveness of Statins for Chronic Obstructive Pulmonary Disease Patients with Pulmonary Hypertension: systematic review and network meta-analysis**

**Table of contents**

*Appendix 1: PRISMA-NMA checklist.................................................................................................................1*

*Appendix 2: Search strategy.............................................................................................................................. 5*

*Appendix 3: Characteristics of included studies ...............................................................................................8*

*Appendix 4: Risk of bias of randomized clinical trials....................................................................................16*

*Appendix 5: Evaluation of consistency and heterogeneity .............................................................................18*

*Appendix 6: Density plots and Trajectory plots of comparisons of each outcome..........................................20*

*Appendix 7: Convergence Diagnostic Plots of comparisons of each outcome................................................34*

*Appendix 8: CINeMA Assessment ...................................................................................................................48*

*Appendix 9: Funnel plots.................................................................................................................................56*

*Appendix 10: Network maps and forest plots of secondary outcomes.............................................................70*

*Appendix 11: SUCRA and cumulative probability plots..................................................................................81*

*Appendix 12: league table of Summary Estimates for Statins on PH-COPD of 41 Trials..............................94*

*Appendix 13: Sensitivity analyses..................................................................................................................107*

*Appendix 14: The meta-regression of the factors that may lead to differences to the outcome indicators...108*

**Appendix 1: PRISMA NMA Checklist**

| **Section/Topic** | **Item #** | **Checklist Item** | **Reported on Page #** |
| --- | --- | --- | --- |
| **TITLE** |  |  |  |
| Title | 1 | Identify the report as a systematic review *incorporating a network meta-analysis (or related form of meta-analysis).* | ***1*** |
|  |  |  |  |
| **ABSTRACT** |  |  |  |
| Structured summary | 2 | Provide a structured summary including, as applicable:  **Background:** main objectives  **Methods:** data sources; study eligibility criteria, participants, and interventions; study appraisal; and *synthesis methods, such as network meta-analysis.*  **Results:** number of studies and participants identified; summary estimates with corresponding confidence/credible intervals; *treatment rankings may also be discussed. Authors may choose to summarize pairwise comparisons against a chosen treatment included in their analyses for brevity.*  **Discussion/Conclusions:** limitations; conclusions and implications of findings.  **Other:** primary source of funding; systematic review registration number with registry name. | 2 |
|  |  |  |  |
| **INTRODUCTION** |  |  |  |
| Rationale | 3 | Describe the rationale for the review in the context of what is already known*, including mention of why a network meta-analysis has been conducted.* | ***3*** |
| Objectives | 4 | Provide an explicit statement of questions being addressed, with reference to participants, interventions, comparisons, outcomes, and study design (PICOS). | 4 |
|  |  |  |  |
| **METHODS** |  |  |  |
| Protocol and registration | 5 | Indicate whether a review protocol exists and if and where it can be accessed (e.g., Web address); and, if available, provide registration information, including registration number. | 4 |
| Eligibility criteria | 6 | Specify study characteristics (e.g., PICOS, length of follow-up) and report characteristics (e.g., years considered, language, publication status) used as criteria for eligibility, giving rationale. *Clearly describe eligible treatments included in the treatment network, and note whether any have been clustered or merged into the same node (with justification).* | 4 |
| Information sources | 7 | Describe all information sources (e.g., databases with dates of coverage, contact with study authors to identify additional studies) in the search and date last searched. | 4 |
| Search | 8 | Present full electronic search strategy for at least one database, including any limits used, such that it could be repeated. | 4 |
| Study selection | 9 | State the process for selecting studies (i.e., screening, eligibility, included in systematic review, and, if applicable, included in the meta-analysis). | 4 |
| Data collection process | 10 | Describe method of data extraction from reports (e.g., piloted forms, independently, in duplicate) and any processes for obtaining and confirming data from investigators. | 5 |
| Data items | 11 | List and define all variables for which data were sought (e.g., PICOS, funding sources) and any assumptions and simplifications made. | 5 |
| **Geometry of the network** | **S1** | Describe methods used to explore the geometry of the treatment network under study and potential biases related to it. This should include how the evidence base has been graphically summarized for presentation, and what characteristics were compiled and used to describe the evidence base to readers. | ***7*** |
| Risk of bias within individual studies | 12 | Describe methods used for assessing risk of bias of individual studies (including specification of whether this was done at the study or outcome level), and how this information is to be used in any data synthesis. | 6 |
| Summary measures | 13 | State the principal summary measures (e.g., risk ratio, difference in means). *Also describe the use of additional summary measures assessed, such as treatment rankings and surface under the cumulative ranking curve (SUCRA) values, as well as modified approaches used to present summary findings from meta-analyses.* | 7 |
| Planned methods of analysis | 14 | Describe the methods of handling data and combining results of studies for each network meta-analysis. This should include, but not be limited to:   - *Handling of multi-arm trials;* - *Selection of variance structure;* - *Selection of prior distributions in Bayesian analyses; and* - *Assessment of model fit.* | 7 |
| **Assessment of Inconsistency** | **S2** | Describe the statistical methods used to evaluate the agreement of direct and indirect evidence in the treatment network(s) studied. Describe efforts taken to address its presence when found. | 7 |
| Risk of bias across studies | 15 | Specify any assessment of risk of bias that may affect the cumulative evidence (e.g., publication bias, selective reporting within studies). | **6** |
| Additional analyses | 16 | Describe methods of additional analyses if done, indicating which were pre-specified. This may include, but not be limited to, the following:   - Sensitivity or subgroup analyses; - Meta-regression analyses; - *Alternative formulations of the treatment network; and* - *Use of alternative prior distributions for Bayesian analyses (if applicable).* | ***7*** |
| **RESULTS†** |  |  |  |
| Study selection | 17 | Give numbers of studies screened, assessed for eligibility, and included in the review, with reasons for exclusions at each stage, ideally with a flow diagram. | 7 |
| **Presentation of network structure** | **S3** | Provide a network graph of the included studies to enable visualization of the geometry of the treatment network. | ***Appendix 10*** |
| **Summary of network geometry** | **S4** | Provide a brief overview of characteristics of the treatment network. This may include commentary on the abundance of trials and randomized patients for the different interventions and pairwise comparisons in the network, gaps of evidence in the treatment network, and potential biases 7reflected by the network structure. | ***Appendix 10*** |
| Study characteristics | 18 | For each study, present characteristics for which data were extracted (e.g., study size, PICOS, follow-up period) and provide the citations. | 7 |
| Risk of bias within studies | 19 | Present data on risk of bias of each study and, if available, any outcome level assessment. | 8 |
| Results of individual studies | 20 | For all outcomes considered (benefits or harms), present, for each study: 1) simple summary data for each intervention group, and 2) effect estimates and confidence intervals. *Modified approaches may be needed to deal with information from larger networks.* | 8 |
| Synthesis of results | 21 | Present results of each meta-analysis done, including confidence/credible intervals. *In larger networks, authors may focus on comparisons versus a particular comparator (e.g. placebo or standard care), with full findings presented in an appendix. League tables and forest plots may be considered to summarize pairwise comparisons.* If additional summary measures were explored (such as treatment rankings), these should also be presented. | 9 |
| **Exploration for inconsistency** | **S5** | Describe results from investigations of inconsistency. This may include such information as measures of model fit to compare consistency and inconsistency models, *P* values from statistical tests, or summary of inconsistency estimates from different parts of the treatment network. | ***Appendix 5*** |
| Risk of bias across studies | 22 | Present results of any assessment of risk of bias across studies for the evidence base being studied. | 8 |
| Results of additional analyses | 23 | Give results of additional analyses, if done (e.g., sensitivity or subgroup analyses, meta-regression analyses*, alternative network geometries studied, alternative choice of prior distributions for Bayesian analyses,* and so forth). |  |
| **DISCUSSION** |  |  |  |
| Summary of evidence | 24 | Summarize the main findings, including the strength of evidence for each main outcome; consider their relevance to key groups (e.g., healthcare providers, users, and policy-makers). | 14 |
| Limitations | 25 | Discuss limitations at study and outcome level (e.g., risk of bias), and at review level (e.g., incomplete retrieval of identified research, reporting bias). *Comment on the validity of the assumptions, such as transitivity and consistency. Comment on any concerns regarding network geometry (e.g., avoidance of certain comparisons).* | 14 |
| Conclusions | 26 | Provide a general interpretation of the results in the context of other evidence, and implications for future research. | 16 |
| **FUNDING** |  |  |  |
| Funding | 27 | Describe sources of funding for the systematic review and other support (e.g., supply of data); role of funders for the systematic review. This should also include information regarding whether funding has been received from manufacturers of treatments in the network and/or whether some of the authors are content experts with professional conflicts of interest that could affect use of treatments in the network. | ***17*** |

**Appendix 2: Search strategy**

**Table S1.** Search strategy of PubMed

| **#** | | **Searches** |
| --- | --- | --- |
| 1 | ((((((((((Pulmonary Disease, Chronic Obstructive[MeSH Terms]) OR (Chronic Obstructive Pulmonary Diseases[Title/Abstract])) OR (COPD[Title/Abstract])) OR (Chronic Obstructive Lung Disease[Title/Abstract])) OR (Chronic Obstructive Pulmonary Disease[Title/Abstract])) OR (COAD[Title/Abstract])) OR (Chronic Obstructive Airway Disease[Title/Abstract])) OR (Airflow Obstruction, Chronic[Title/Abstract])) OR (Airflow Obstructions, Chronic[Title/Abstract])) OR (Chronic Airflow Obstructions[Title/Abstract])) OR (Chronic Airflow Obstruction[Title/Abstract]) | |
| 2 | ((((((Hypertension, Pulmonary[MeSH Terms]) OR (Pulmonary Arterial Hypertension[MeSH Terms])) OR (Pulmonary Hypertension[Title/Abstract])) OR (Arterial Hypertension, Pulmonary[Title/Abstract])) OR (Hypertension, Pulmonary Arterial[Title/Abstract])) OR (PAH[Title/Abstract])) OR (PH[Title/Abstract]) | |
| 3 | #1 AND #2 | |
| 4 | (((((((((((((((((((((((((((((Hydroxymethylglutaryl-CoA Reductase Inhibitors[MeSH Terms]) OR (Hydroxymethylglutaryl CoA Reductase Inhibitors[Title/Abstract])) OR (Inhibitors, Hydroxymethylglutaryl-CoA Reductase[Title/Abstract])) OR (Reductase Inhibitors, Hydroxymethylglutaryl-CoA[Title/Abstract])) OR (HMG-CoA Reductase Inhibitor[Title/Abstract])) OR (HMG CoA Reductase Inhibitor[Title/Abstract])) OR (Statin[Title/Abstract])) OR (Statins[Title/Abstract])) OR (Inhibitors, HMG-CoA Reductase[Title/Abstract])) OR (Inhibitors, HMG CoA Reductase[Title/Abstract])) OR (Reductase Inhibitors, HMG-CoA[Title/Abstract])) OR (HMG-CoA Reductase Inhibitors[Title/Abstract])) OR (HMG CoA Reductase Inhibitors[Title/Abstract])) OR (Inhibitors, Hydroxymethylglutaryl-Coenzyme A[Title/Abstract])) OR (Hydroxymethylglutaryl-Coenzyme A Inhibitors[Title/Abstract])) OR (Inhibitors, Hydroxymethylglutaryl Coenzyme A[Title/Abstract])) OR (Inhibitors, Hydroxymethylglutaryl-CoA[Title/Abstract])) OR (Hydroxymethylglutaryl-CoA Inhibitors[Title/Abstract])) OR (Inhibitors, Hydroxymethylglutaryl CoA[Title/Abstract])) OR (Hydroxymethylglutaryl-CoA Reductase Inhibitor[Title/Abstract])) OR (Hydroxymethylglutaryl CoA Reductase Inhibitor[Title/Abstract])) OR (Reductase Inhibitor, Hydroxymethylglutaryl-CoA[Title/Abstract])) OR (Statins, HMG-CoA[Title/Abstract])) OR (HMG-CoA Statins[Title/Abstract])) OR (Statins, HMG CoA[Title/Abstract])) OR (Lovastatin[Title/Abstract])) OR (Simvastatin[Title/Abstract])) OR (Pitavastatin[Title/Abstract])) OR (Pravastatin[Title/Abstract])) OR (Rosuvastatin[Title/Abstract]) | |
| 5 | ((((Randomized Controlled Trials as Topic[MeSH Terms]) OR (Clinical Trials, Randomized[Title/Abstract])) OR (Trials, Randomized Clinical[Title/Abstract])) OR (Controlled Clinical Trials, Randomized[Title/Abstract])) OR (RCT[Title/Abstract]) | |
| 6 | #3 AND #4 AND #5 | |

**Table S2.** Search strategy of Web of Science

| **#** | **Searches** |
| --- | --- |
| 1 | TS=(Chronic Obstructive Pulmonary Disease OR COPD OR Chronic Obstructive Lung Disease OR Chronic Obstructive Airway Disease OR Airflow Obstruction, Chronic OR Chronic Airflow Obstruction) |
| 2 | TS=(Pulmonary Hypertension OR Pulmonary Arterial Hypertension OR PAH OR PH) |
| 3 | #1 AND #2 |
| 4 | TS=(Hydroxymethylglutaryl-CoA Reductase Inhibitors OR HMG-CoA Reductase Inhibitors OR Statins OR Lovastatin OR Simvastatin OR Pitavastatin OR Pravastatin OR Rosuvastatin) |
| 5 | TS=(Randomized Controlled Trials OR Clinical Trials, Randomized OR RCT) |
| 6 | #3 AND #4 AND #5 |

**Table S3.** Search strategy of Cochrane Central Register of Controlled Trials

| **#** | **Searches** |
| --- | --- |
| 1 | ("Chronic Obstructive Pulmonary Disease":ti,ab,kw OR COPD:ti,ab,kw OR "Chronic Obstructive Lung Disease":ti,ab,kw OR "Chronic Obstructive Airway Disease":ti,ab,kw OR "Airflow Obstruction, Chronic":ti,ab,kw OR "Chronic Airflow Obstruction":ti,ab,kw) |
| 2 | ("Pulmonary Hypertension":ti,ab,kw OR "Pulmonary Arterial Hypertension":ti,ab,kw OR PAH:ti,ab,kw OR PH:ti,ab,kw) |
| 3 | #1 AND #2 |
| 4 | ("Hydroxymethylglutaryl-CoA Reductase Inhibitors":ti,ab,kw OR "HMG-CoA Reductase Inhibitors":ti,ab,kw OR Statins:ti,ab,kw OR Lovastatin:ti,ab,kw OR Simvastatin:ti,ab,kw OR Pitavastatin:ti,ab,kw OR Pravastatin:ti,ab,kw OR Rosuvastatin:ti,ab,kw) |
| 5 | ("Randomized Controlled Trials":ti,ab,kw OR "Clinical Trials, Randomized":ti,ab,kw OR RCT:ti,ab,kw) |
| 6 | #3 AND #4 AND #5 |

**Table S4.** Search strategy of Embase

| **#** | **Searches** |
| --- | --- |
| 1 | ('chronic obstructive pulmonary disease'/exp OR 'chronic obstructive pulmonary disease':ti,ab,kw OR COPD:ti,ab,kw OR 'chronic obstructive lung disease':ti,ab,kw OR 'chronic obstructive airway disease':ti,ab,kw OR 'airflow obstruction, chronic':ti,ab,kw OR 'chronic airflow obstruction':ti,ab,kw) |
| 2 | ('pulmonary hypertension'/exp OR 'pulmonary hypertension':ti,ab,kw OR 'pulmonary arterial hypertension':ti,ab,kw OR PAH:ti,ab,kw OR PH:ti,ab,kw) |
| 3 | #1 AND #2 |
| 4 | ('hydroxymethylglutaryl-coa reductase inhibitor'/exp OR 'hydroxymethylglutaryl-coa reductase inhibitor':ti,ab,kw OR 'hmg-coa reductase inhibitor':ti,ab,kw OR statin:ti,ab,kw OR lovastatin:ti,ab,kw OR simvastatin:ti,ab,kw OR pitavastatin:ti,ab,kw OR pravastatin:ti,ab,kw OR rosuvastatin:ti,ab,kw) |
| 5 | ('randomized controlled trial'/exp OR 'randomized controlled trial':ti,ab,kw OR 'clinical trial, randomized':ti,ab,kw OR RCT:ti,ab,kw) |
| 6 | #3 AND #4 AND #5 |

**Appendix 3: Characteristics of included interventions**

**Table S3.1:** Statins list

| Statins | Synonyms | Drug Type | Company | Highest Phase | Drug-Approved Country/Region | Anatomical Therapeutic Chemical |
| --- | --- | --- | --- | --- | --- | --- |
| Atorvastatin | - Lipitor - Atorvastatin Calcium - Atorvastatin, Calcium Salt - Atorvastatin Calcium Anhydrous - Liptonorm - Atorvastatin Calcium Hydrate - CI 981 - Atorvastatin Calcium Trihydrate | HMG-CoA reductase inhibitor (Statin) | Pfizer | Approved | Worldwide (USA, EU, Japan, etc.) | C10AA05 |
| Rosuvastatin | - Rosuvastatin Calcium - ZD 4522 - Crestor | HMG-CoA reductase inhibitor (Statin) | AstraZeneca | Approved | Worldwide (USA, EU, Japan, etc.) | C10AA07 |
| Fluvastatin | - Lescol - XU 62-320 - Fluvastatin Sodium - Fluindostatin - Fluvastatin Sodium Salt | HMG-CoA reductase inhibitor (Statin) | Novartis | Approved | Worldwide (USA, EU, Japan, etc.) | C10AA04 |
| Simvastatin | - Zocor - MK-733 - Synvinolin | HMG-CoA reductase inhibitor (Statin) | Merck | Approved | Worldwide (USA, EU, Japan, etc.) | C10AA01 |
| Pravastatin | - Eptastatin - SQ-31000 - Pravastatin tert-Octylamine Salt - Pravastatin Sodium - Pravastatin Sodium Salt - CS-514 | HMG-CoA reductase inhibitor (Statin) | Bristol-Myers Squibb | Approved | Worldwide (USA, EU, Japan, etc.) | C10AA03 |

**Appendix 4: Risk of bias of randomized clinical trials**

**Figure S4:** Overall risk of bias presented as percentage of each risk of bias item across all included studies.

Green = Low risk, Red = High risk, Yellow = Some concerns.


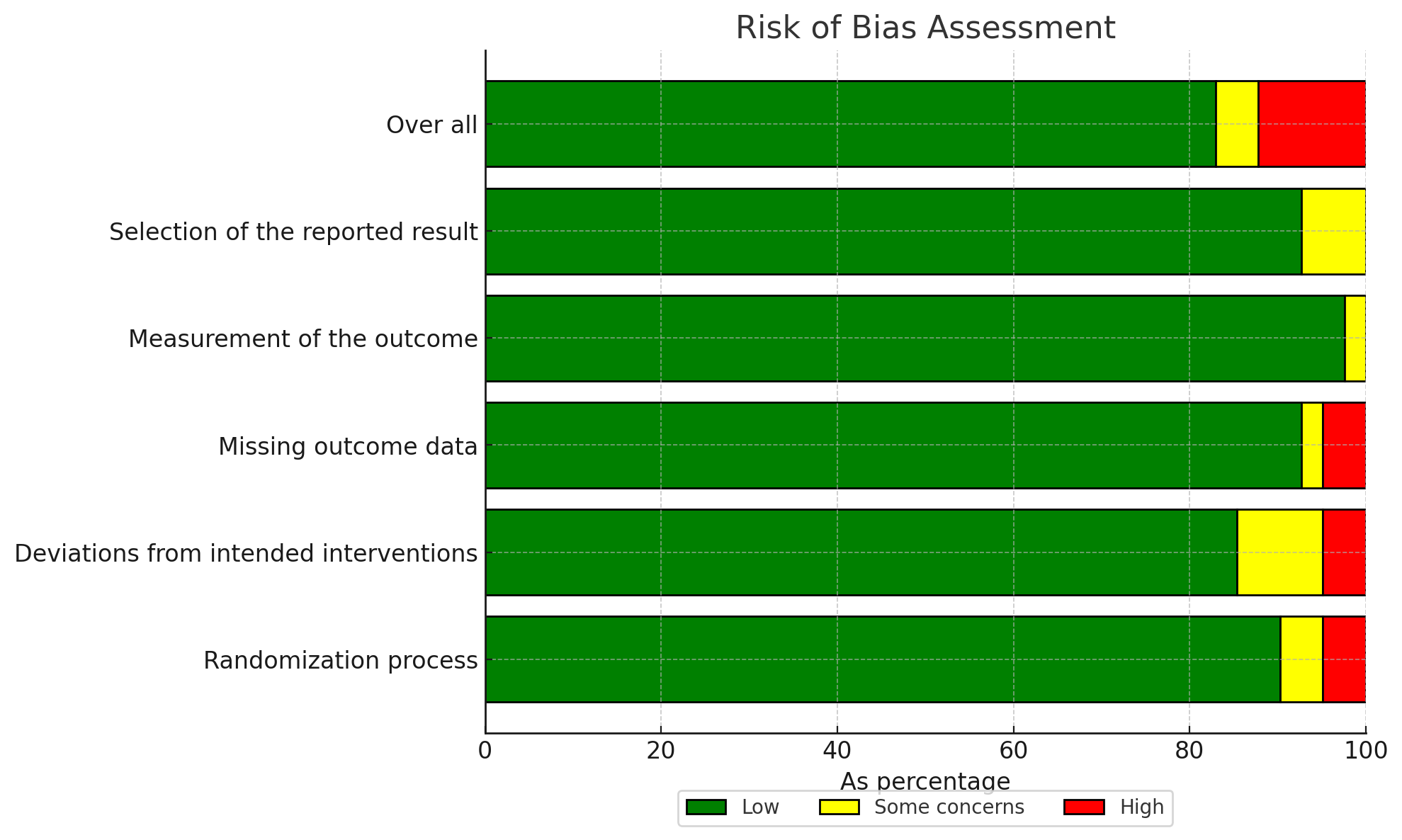


| Unique ID | Randomization process | Deviations from intended interventions | Missing outcome data | Measurement of the outcome | Selection of the reported result | Over all |
| --- | --- | --- | --- | --- | --- | --- |
| Cao 20181 | High | High | High | Low | Some concerns | High |
| Qu 20122 | Low | Low | High | Low | Some concerns | High |
| He 20193 | Low | Low | Low | Low | Low | Low |
| Yu 20124 | Low | Low | Low | Low | Low | Low |
| Luo 20135 | High | High | Some concerns | Some concerns | Some concerns | High |
| Wang 20156 | Low | Some concerns | Low | Low | Low | Some concerns |
| Yan 20187 | Low | Some concerns | Low | Low | Low | Some concerns |
| Mao 20178 | Low | Low | Low | Low | Low | Low |
| Zhang 20199 | Low | Low | Low | Low | Low | Low |
| Zhang 201310 | Low | Low | Low | Low | Low | Low |
| Wu 201411 | Low | Low | Low | Low | Low | Low |
| Deng 201512 | Low | Low | Low | Low | Low | Low |
| Liu 201613 | Low | Low | Low | Low | Low | Low |
| Li 201214 | Low | Low | Low | Low | Low | Low |
| Sun 202015 | Low | Low | Low | Low | Low | Low |
| Chen 201616 | Low | Low | Low | Low | Low | Low |
| Jiang 201517 | Low | Low | Low | Low | Low | Low |
| Niu 201518 | Low | Low | Low | Low | Low | Low |
| Wang 201119 | Low | Low | Low | Low | Low | Low |
| Wang 201220 | Low | Low | Low | Low | Low | Low |
| Xu 202021 | Low | Low | Low | Low | Low | Low |
| Tang 201822 | Low | Low | Low | Low | Low | Low |
| Ren 201823 | Some concerns | Some concerns | Low | Low | Low | High |
| Nan 201624 | Low | Low | Low | Low | Low | Low |
| Ye 201525 | Low | Low | Low | Low | Low | Low |
| Rang 201326 | Low | Low | Low | Low | Low | Low |
| Xia 201327 | Low | Low | Low | Low | Low | Low |
| Chen 201728 | Low | Low | Low | Low | Low | Low |
| Ding 201629 | Low | Low | Low | Low | Low | Low |
| Tang 201730 | Low | Low | Low | Low | Low | Low |
| Zhang 201531 | Low | Low | Low | Low | Low | Low |
| Hu 201932 | Low | Low | Low | Low | Low | Low |
| Tong 201633 | Low | Low | Low | Low | Low | Low |
| Sun 201434 | Low | Low | Low | Low | Low | Low |
| Yan 201235 | Low | Low | Low | Low | Low | Low |
| Liu 201036 | Some concerns | Some concerns | Low | Low | Low | High |
| Arian 201737 | Low | Low | Low | Low | Low | Low |
| Chogtu 201638 | Low | Low | Low | Low | Low | Low |
| Lee 200939 | Low | Low | Low | Low | Low | Low |
| Liu 201340 | Low | Low | Low | Low | Low | Low |
| Moosavi 201341 | Low | Low | Low | Low | Low | Low |

**Table S4:** Study level risk of bias assessment using Cochrane risk of bias tool 2.0 for assessing risk of bias.

**Appendix 5: Evaluation of consistency and heterogeneity**

**Table S5.1:** Evaluation of consistency and heterogeneity

| **Parameters** | **Outcomes** | **Study** | **Consistency** | | | |
| --- | --- | --- | --- | --- | --- | --- |
|  |  |  | **Totresdev** | **pD** | **DIC** | **I²** |
| **Efficacy** | **Pulmonary artery pressure** |  |  |  |  |  |
|  | sPAP | 33 studies, n = 2816 | 63.86611 | 59.02450 | 122.89061 | 0% |
|  | mPAP | 6 studies, n = 532 | 11.91501 | 11.78834 | 23.70335 | 8% |
|  | **Exercise tolerance** |  |  |  |  |  |
|  | 6MWD | 12 studies, n = 1119 | 25.05216 | 23.47900 | 48.53117 | 8% |
|  | **Lung function** |  |  |  |  |  |
|  | FVC | 21 studies, n = 1976 | 41.41004 | 38.04652 | 79.45656 | 1% |
|  | FEV1 | 21 studies, n = 1868 | 41.89376 | 38.69370 | 80.58746 | 2% |
|  | FEV1/FVC | 11 studies, n = 1038 | 22.53627 | 20.42909 | 42.96536 | 7% |
|  | **Oxygenation Parameters** |  |  |  |  |  |
|  | PO2 | 8 studies, n = 766 | 16.44745 | 15.70229 | 32.14974 | 9% |
|  | PCO2 | 7 studies, n = 704 | 14.00268 | 13.88797 | 27.89065 | 7% |
|  | **Inflammatory markers** |  |  |  |  |  |
|  | TNF-α | 8 studies, n = 700 | 16.58665 | 16.32107 | 32.90772 | 4% |
|  | hs-CRP | 18 studies, n = 1684 | 36.75802 | 34.68528 | 71.44329 | 2% |
|  | IL-6 | 10 studies, n = 948 | 20.54803 | 19.73877 | 40.28679 | 3% |
|  | **Vasoactive substances** |  |  |  |  |  |
|  | NO | 11 studies, n = 946 | 22.20785 | 21.14859 | 43.35644 | 5% |
|  | ET-1 | 12 studies, n = 1190 | 24.55807 | 23.60599 | 48.16406 | 2% |

Total residual deviance = totresdev; effective number of parameters = pD; deviance information criterion = DIC. Pulmonary artery pressure(sPAP, Mean Pulmonary Artery Pressure (mPAP)), exercise tolerance(6-Minute Walk Distance (6MWD)), lung function(Forced Vital Capacity (FVC), Forced Expiratory Volume in 1 Second (FEV1), FEV1/FVC), and Oxygenation Parameters(Partial Pressure of Oxygen (PO2), Partial Pressure of Carbon Dioxide (PCO2)) were treated as primary outcomes, while inflammatory markers(Tumor Necrosis Factor-α (TNF-α), High-Sensitivity C-Reactive Protein (hs-CRP), Interleukin-6 (IL-6)) and vasoactive substances(Nitric Oxide (NO), Endothelin-1 (ET-1)) .

**Appendix 6: Density plots of comparisons of each outcome**

**Figure S6.1:** Density plots and Trajectory plots of **sPAP**

**
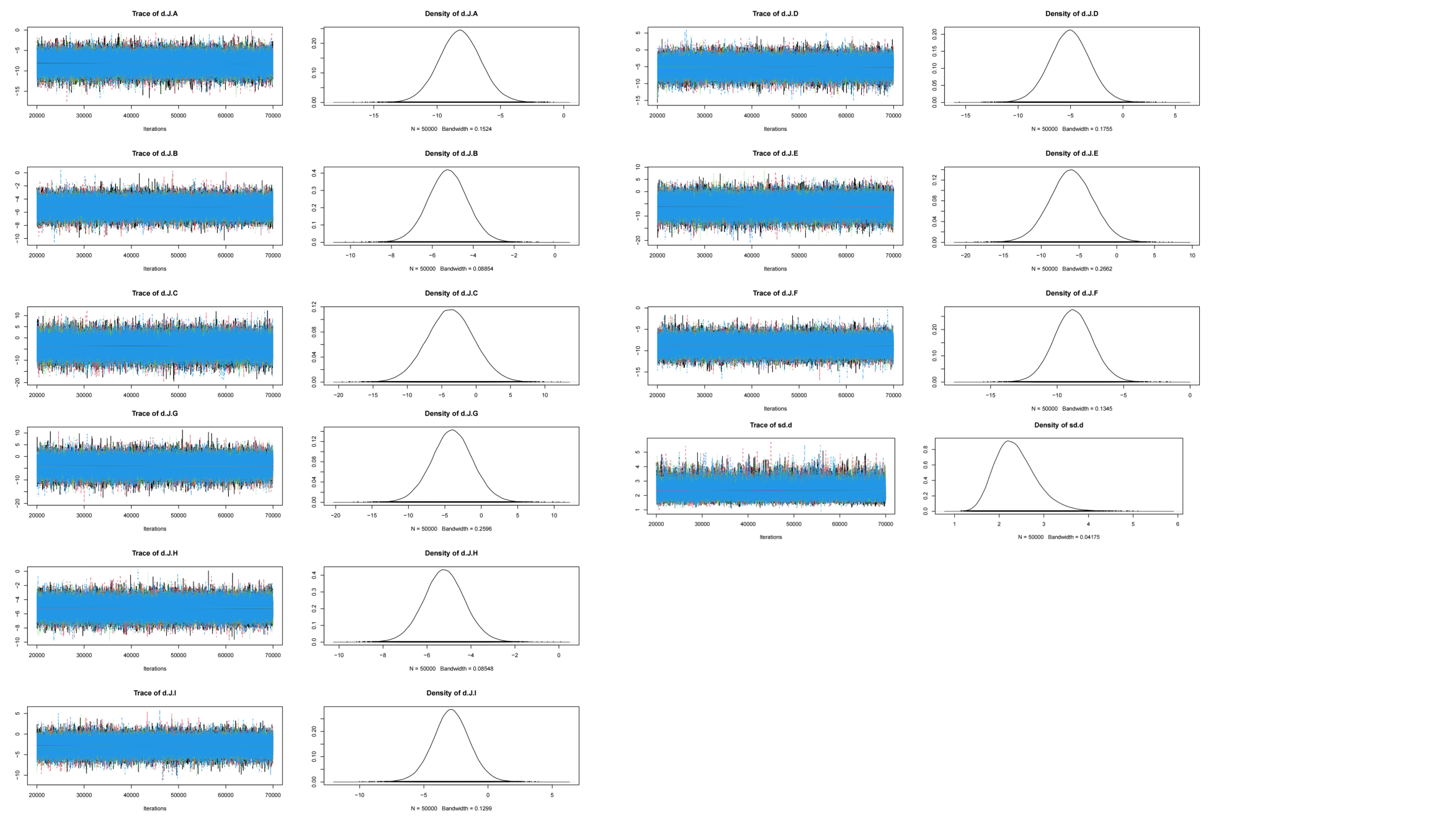
**

**Figure S6.2:** Density plots of **mPAP**

**
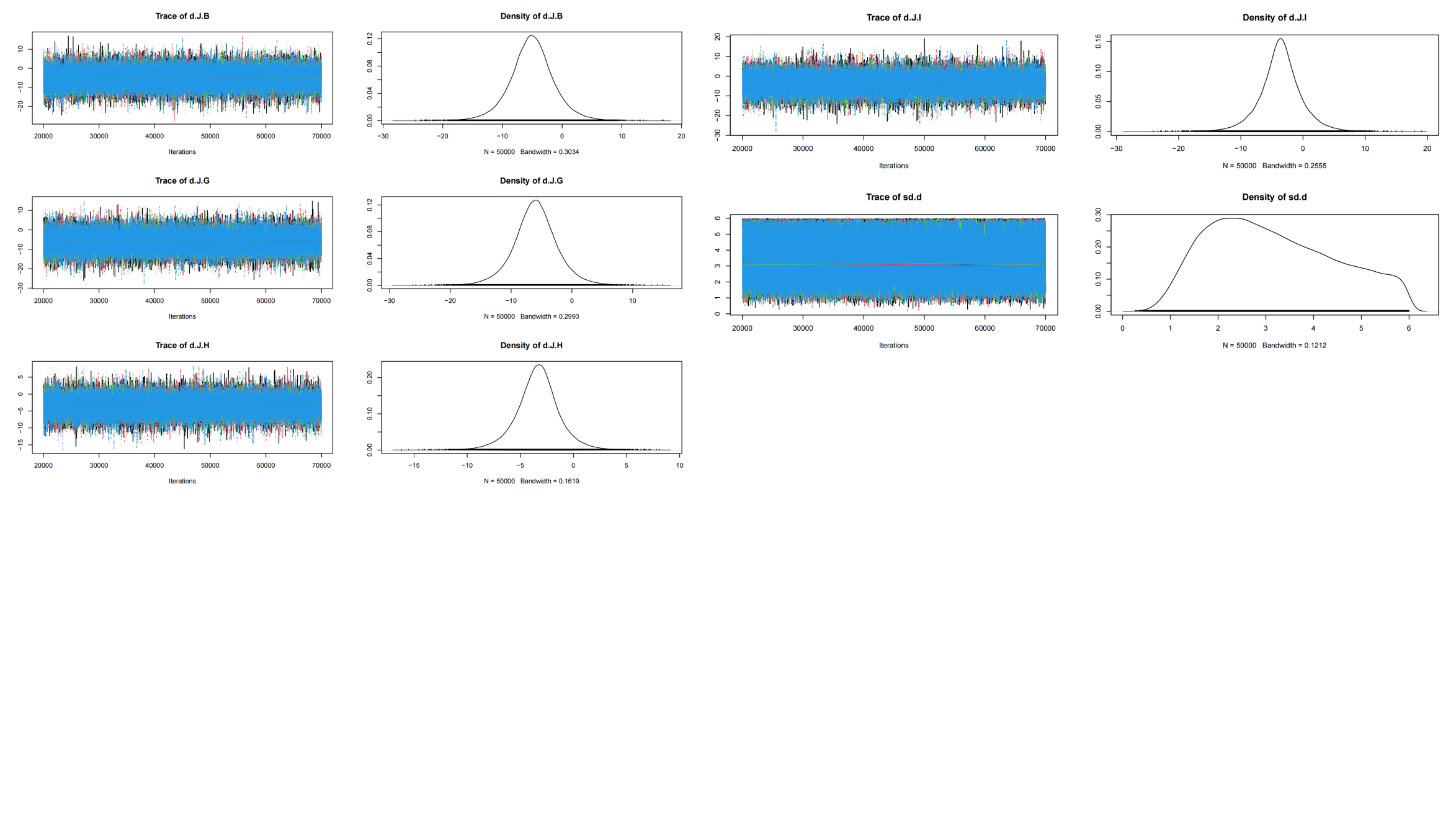
**

**Figure S6.3:** Density plots and Trajectory plots of **6MWD**

**Figure S6.4:** Density plots and Trajectory plots of **FVC**

**Figure S6.5:** Density plots and Trajectory plots of **FEV1**

**
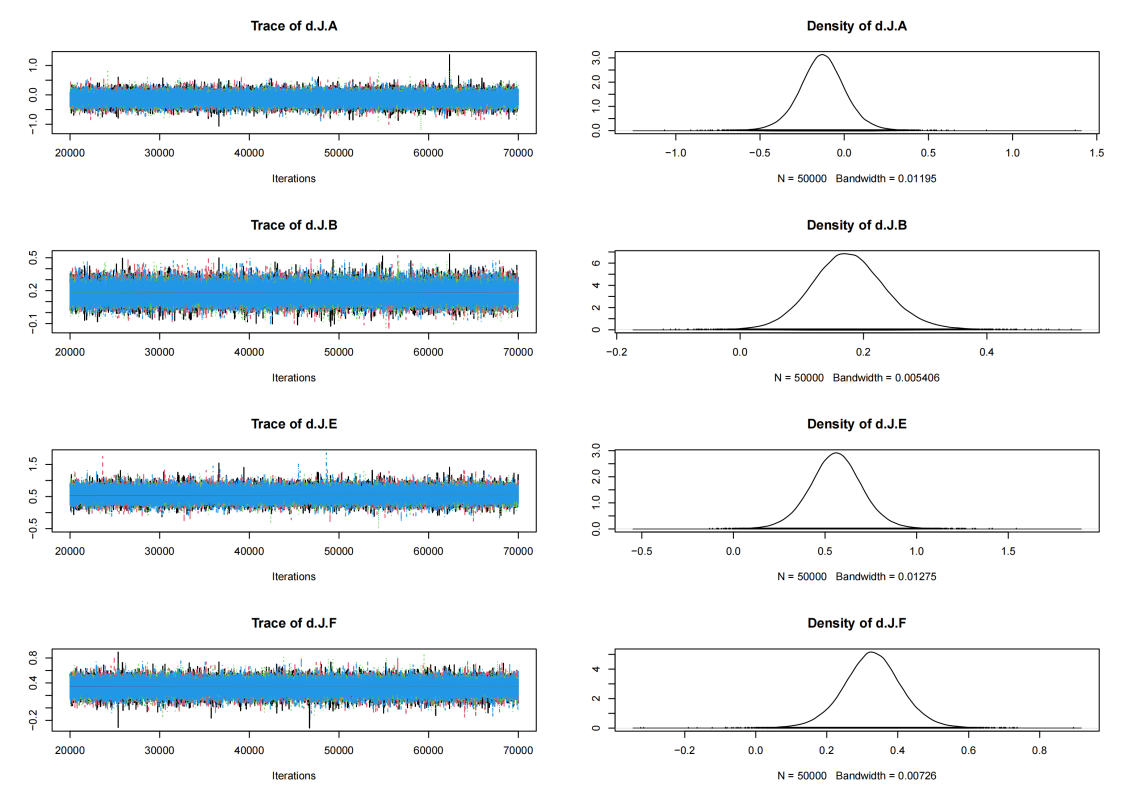

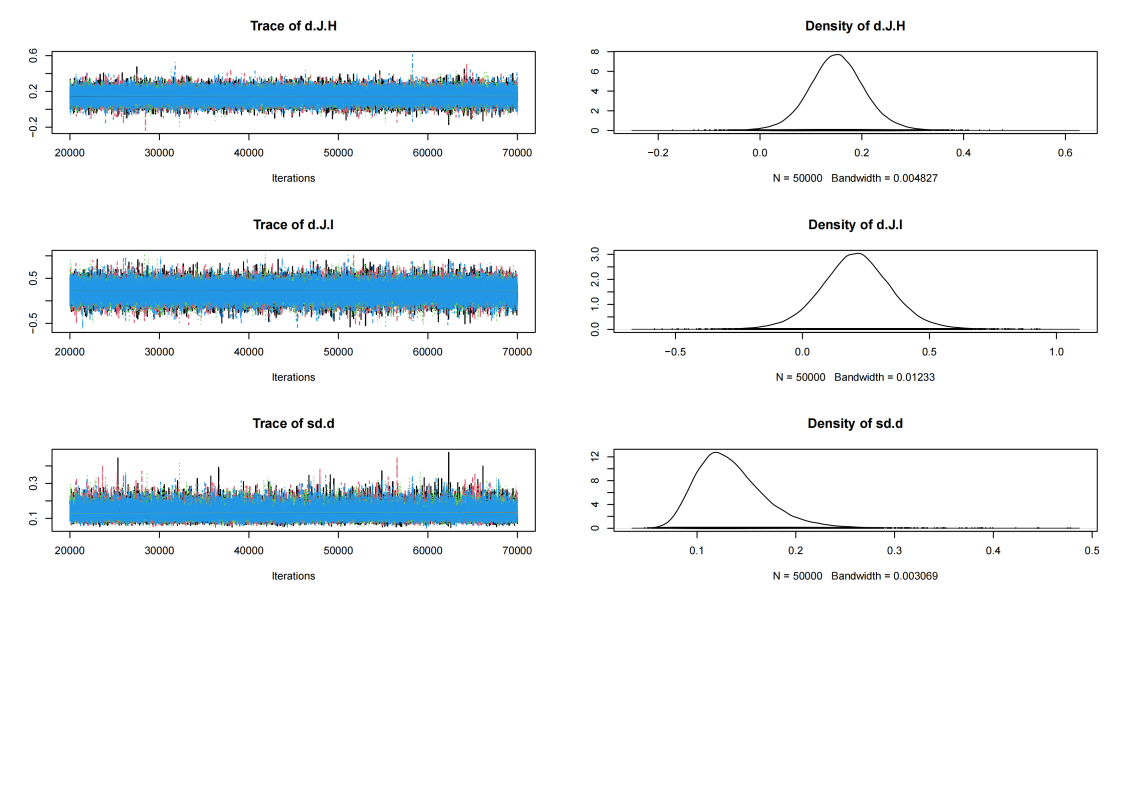
**

**Figure S6.6:** Density plots and Trajectory plots of of **FEV1/FVC**

**
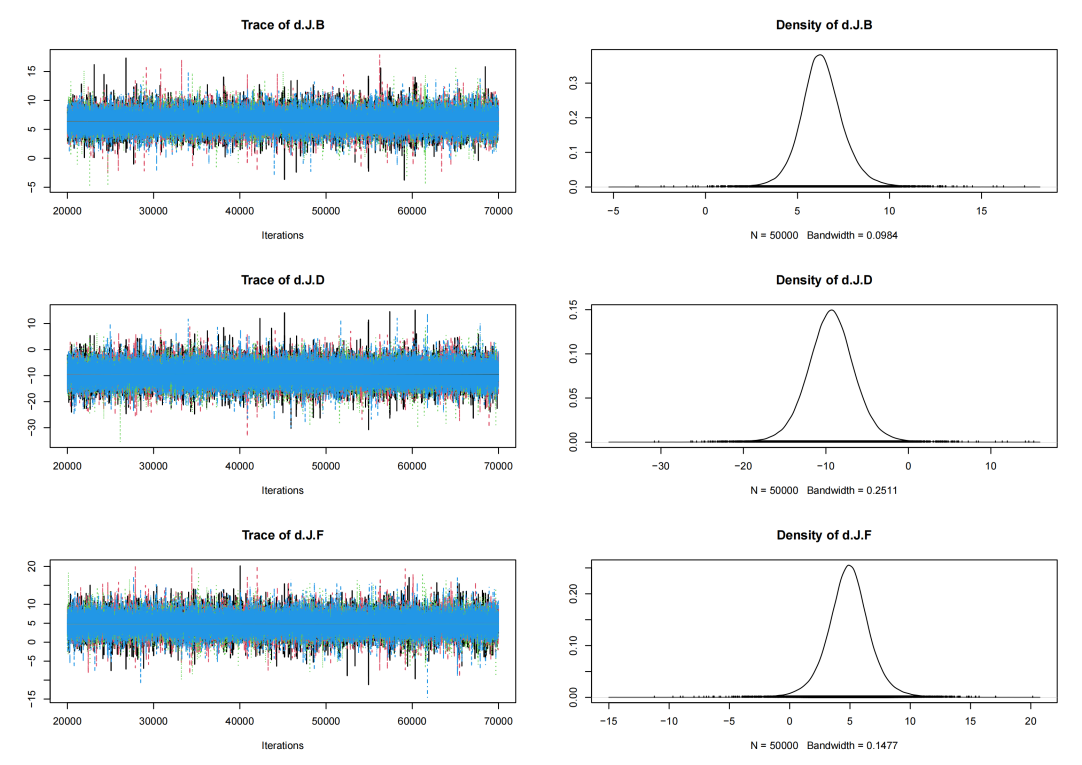

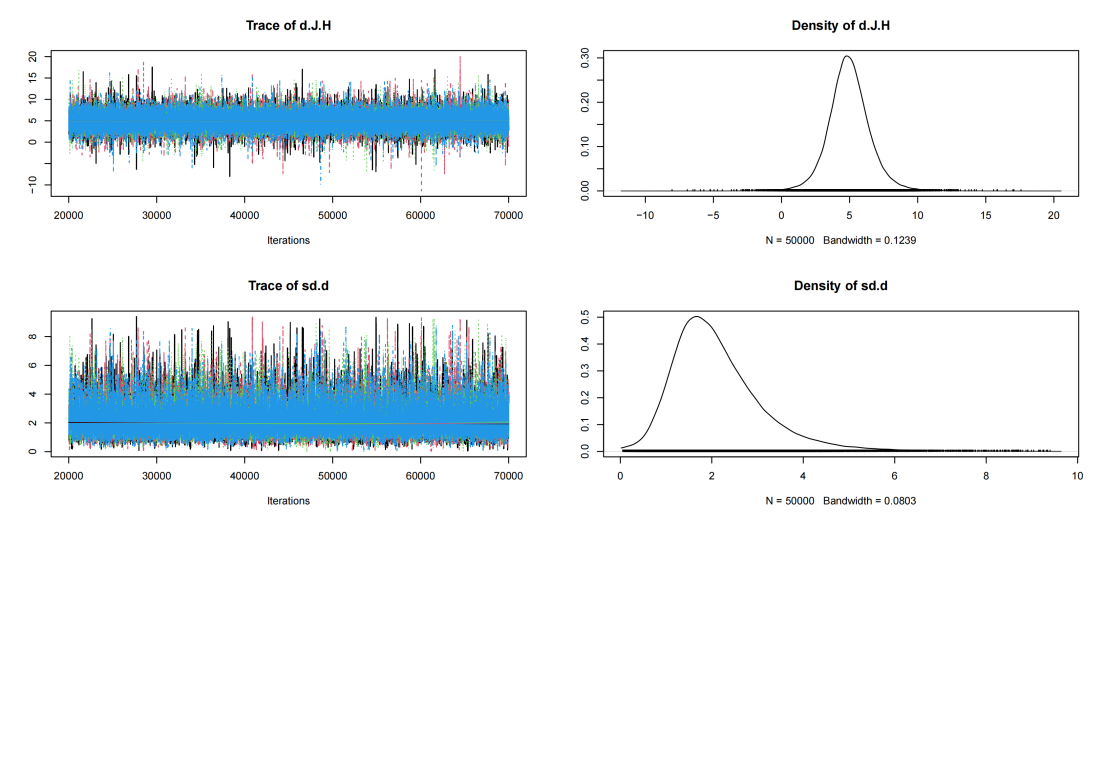
**

**Figure S6.7:** Density plots and Trajectory plots of of **PO2**

**
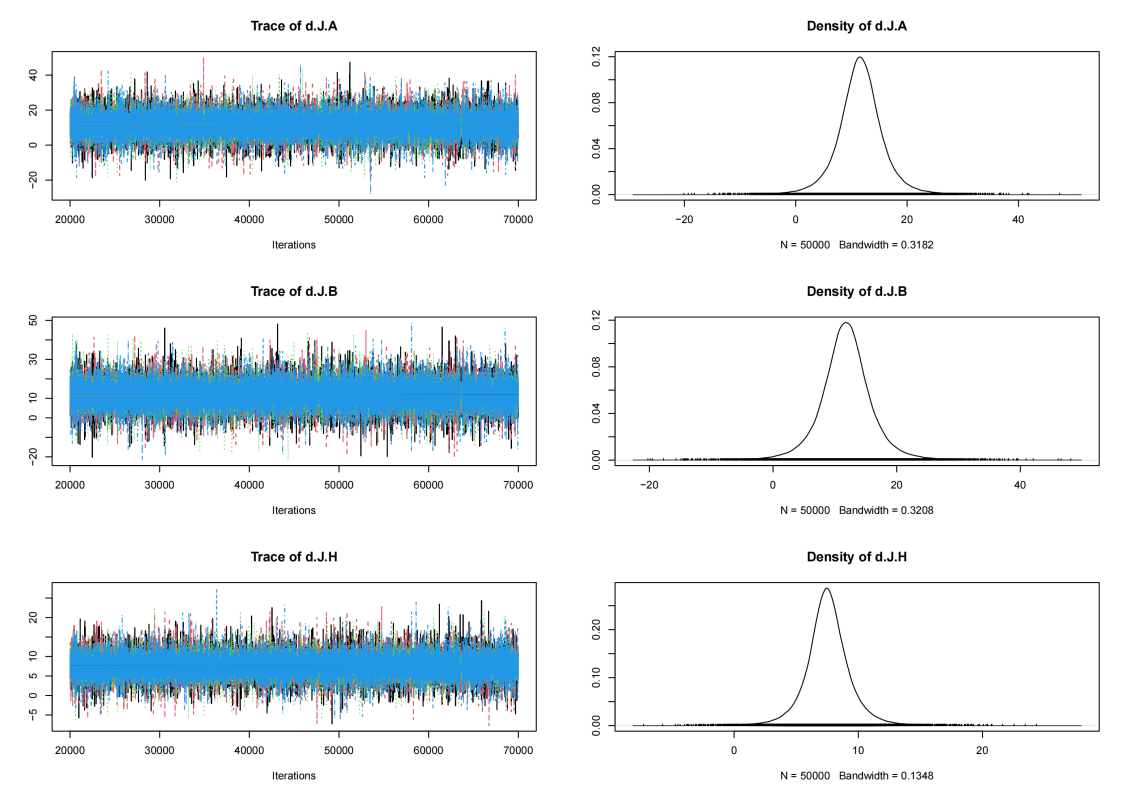

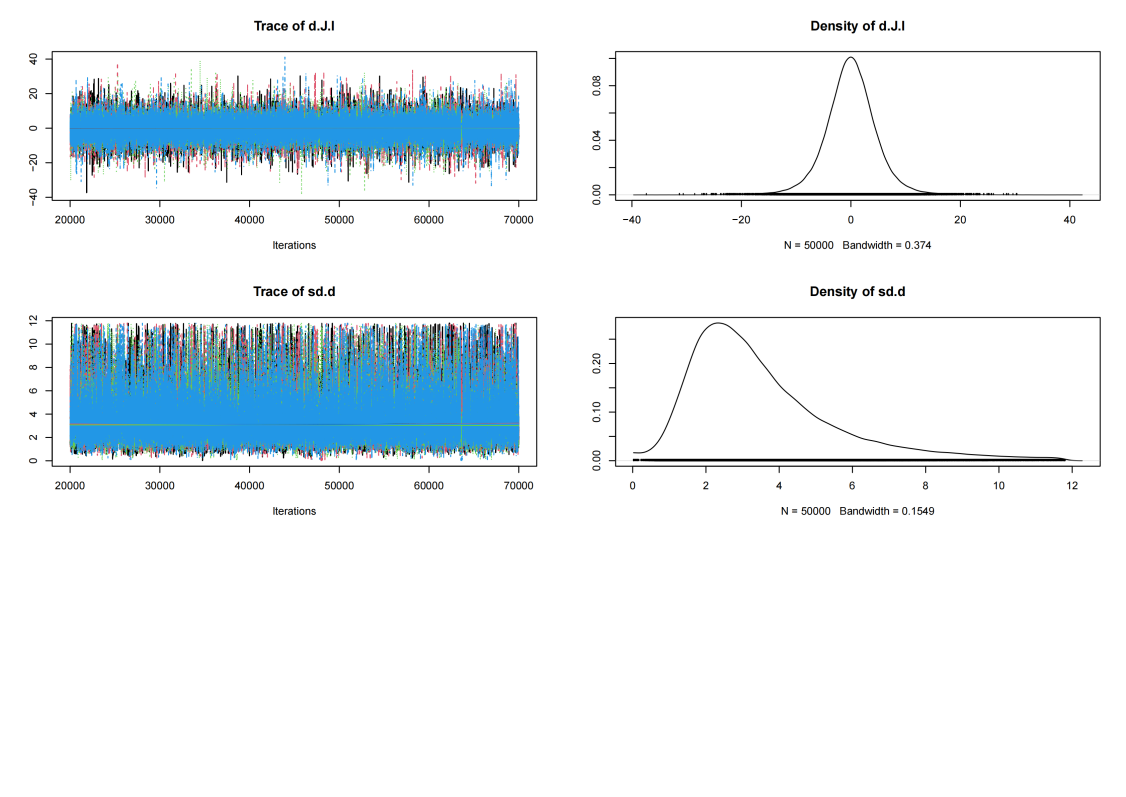
**

**Figure S6.8:** Density plots and Trajectory plots of of **PCO2**

**
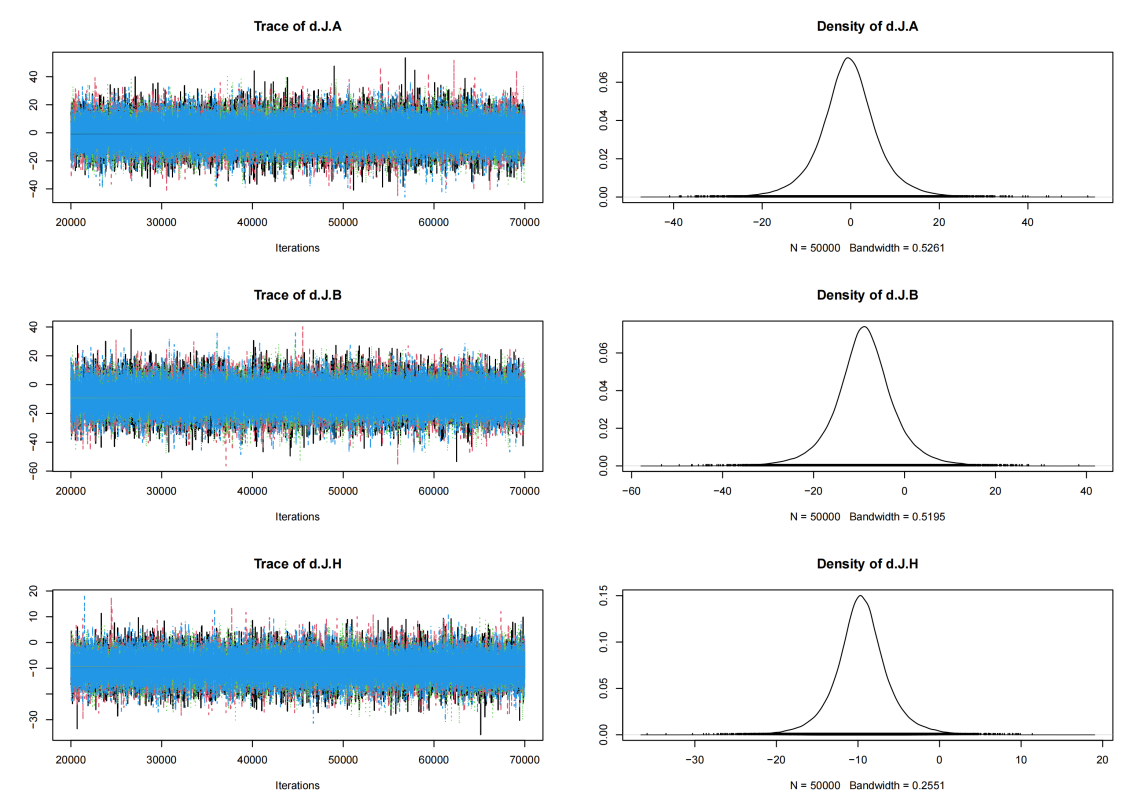

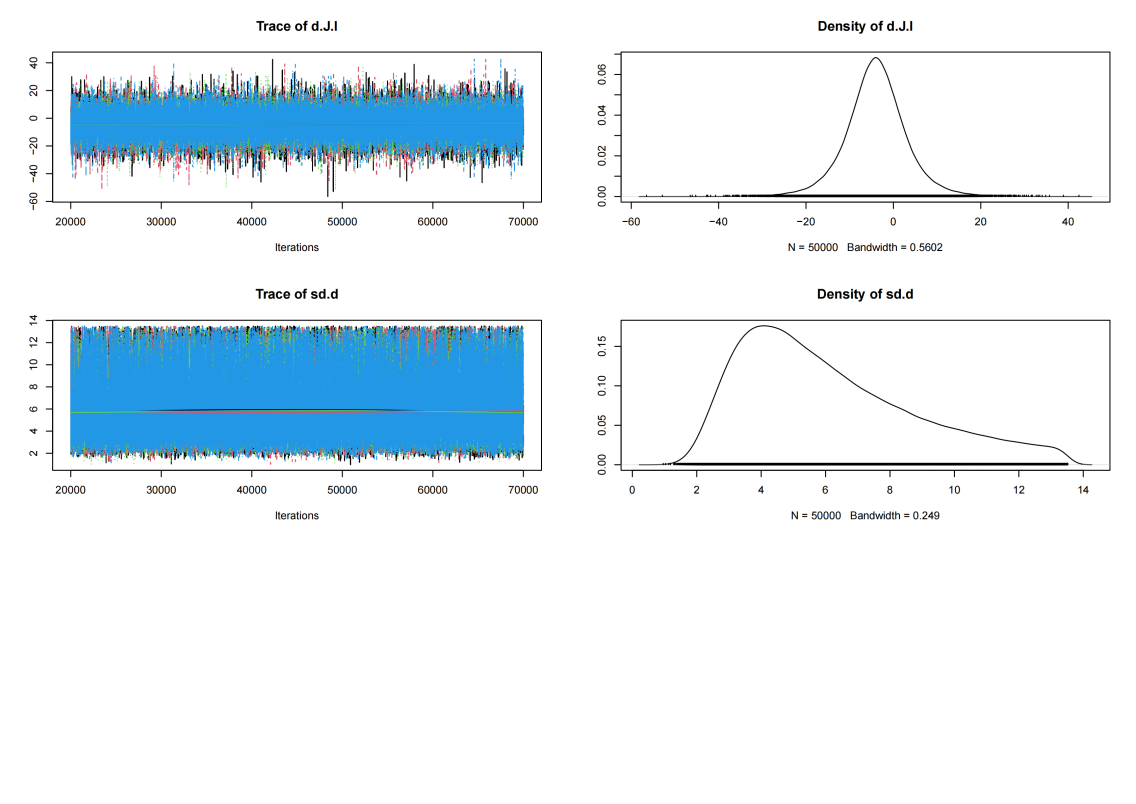
**

**Figure S6.9:** Density plots and Trajectory plots of of **TNF-α**

**
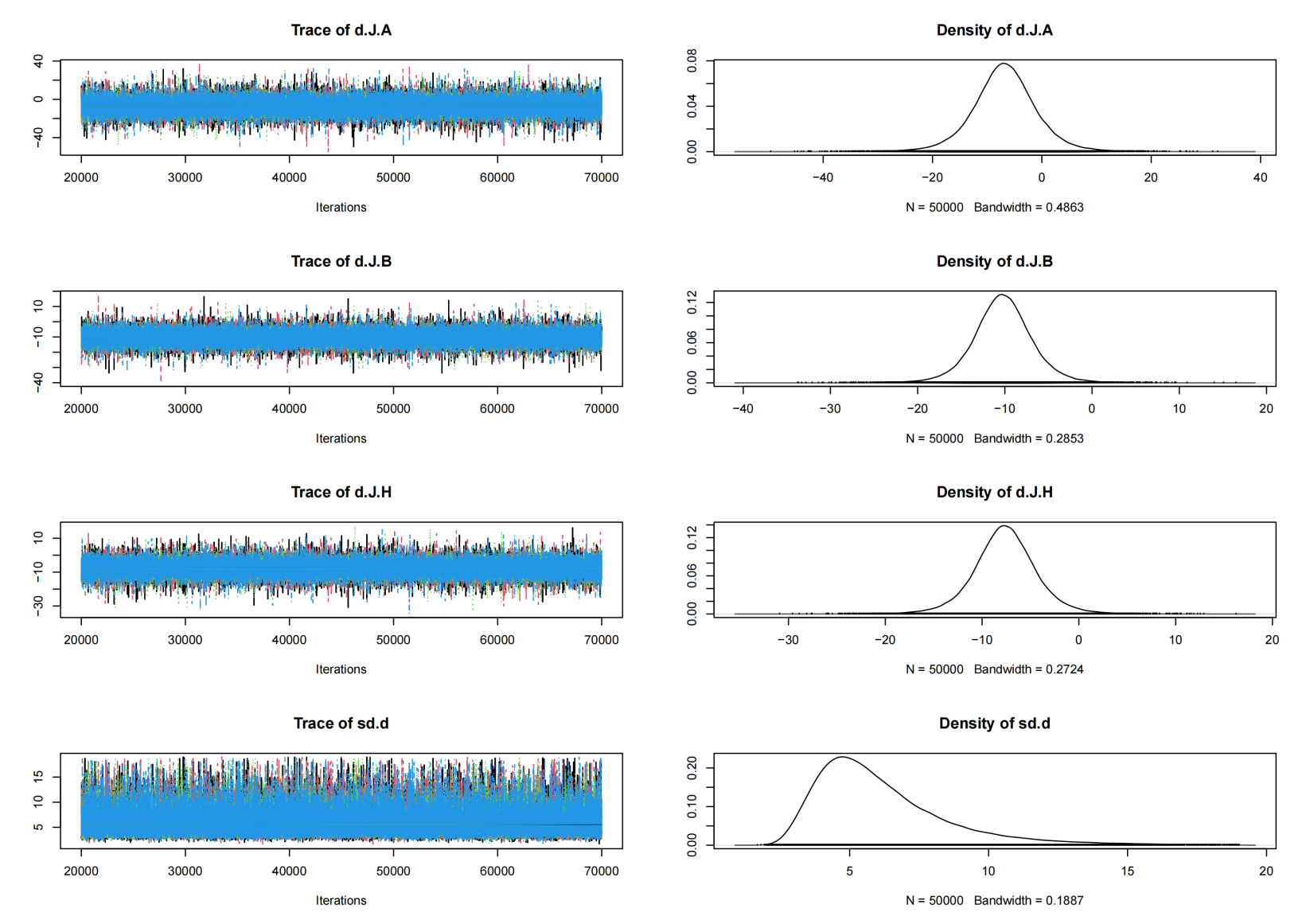
**

**Figure S6.10:** Density plots and Trajectory plots of of **hs-CRP**

**
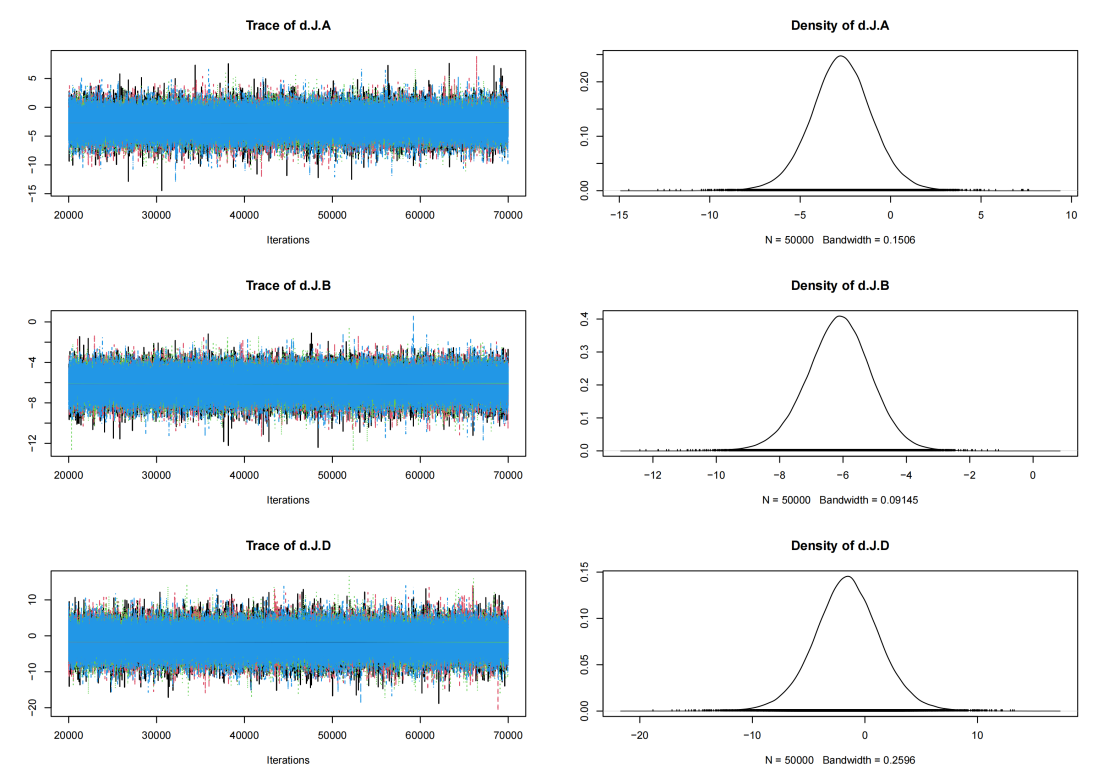

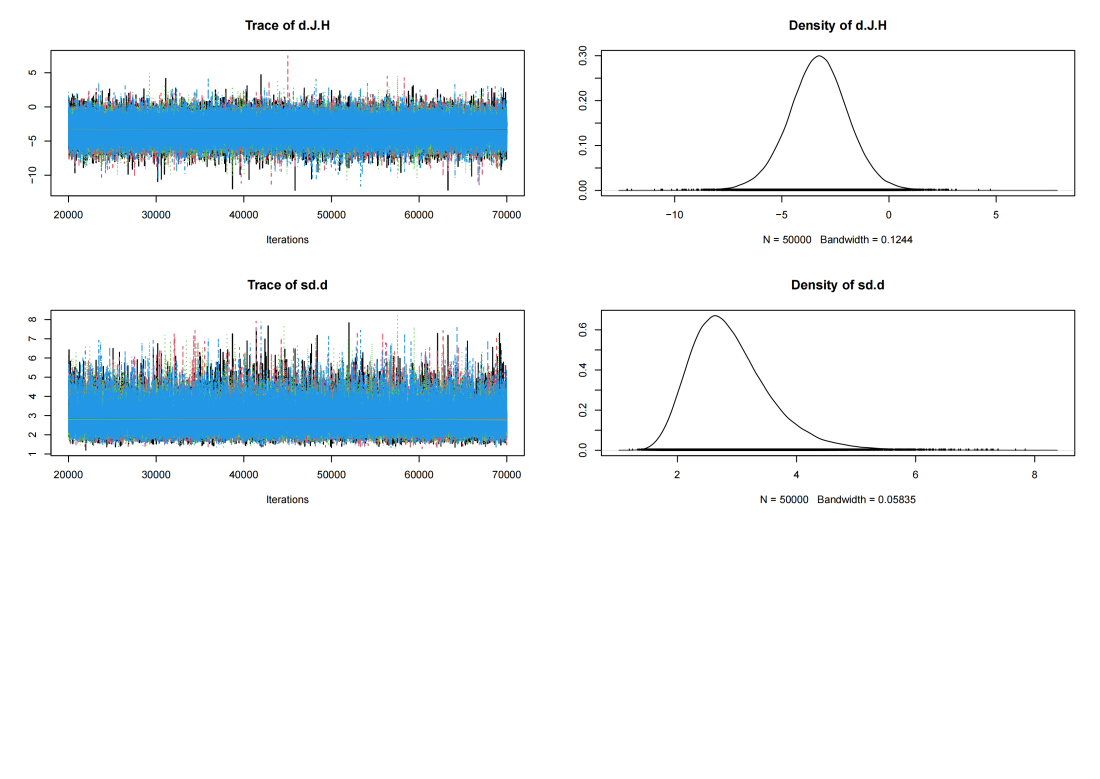
**

**Figure S6.11:** Density plots and Trajectory plots of of **IL-6**

**
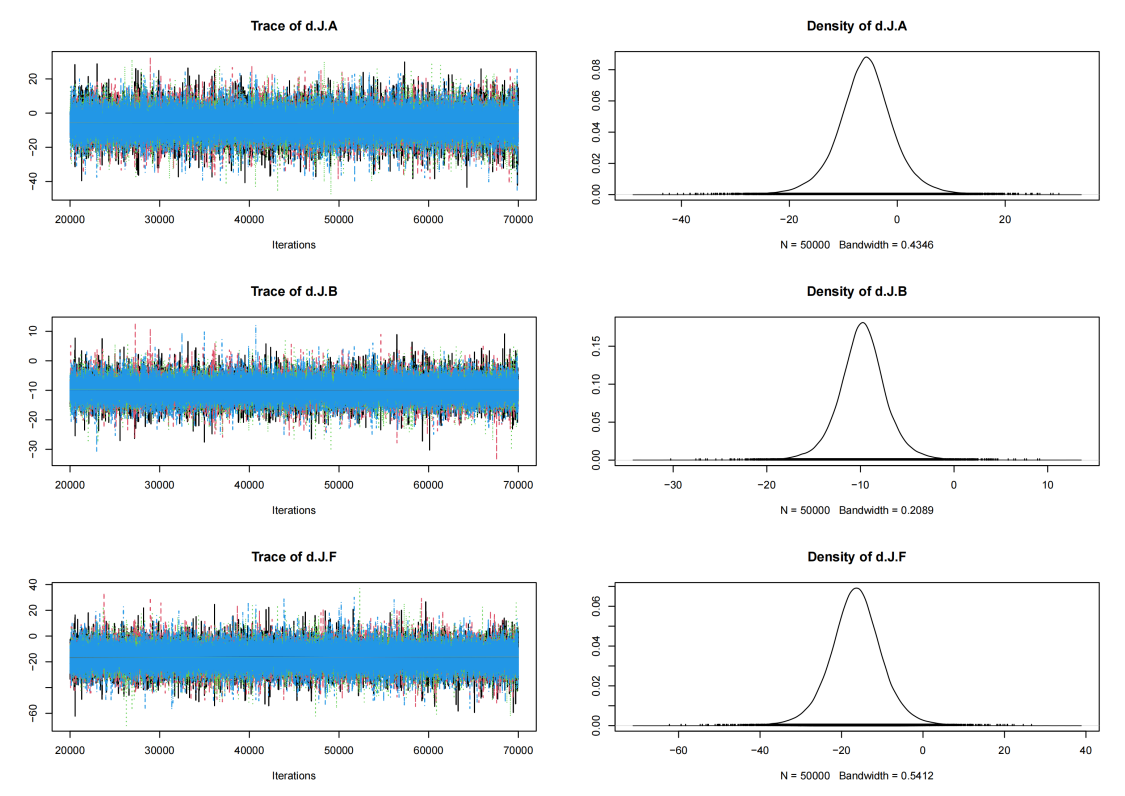

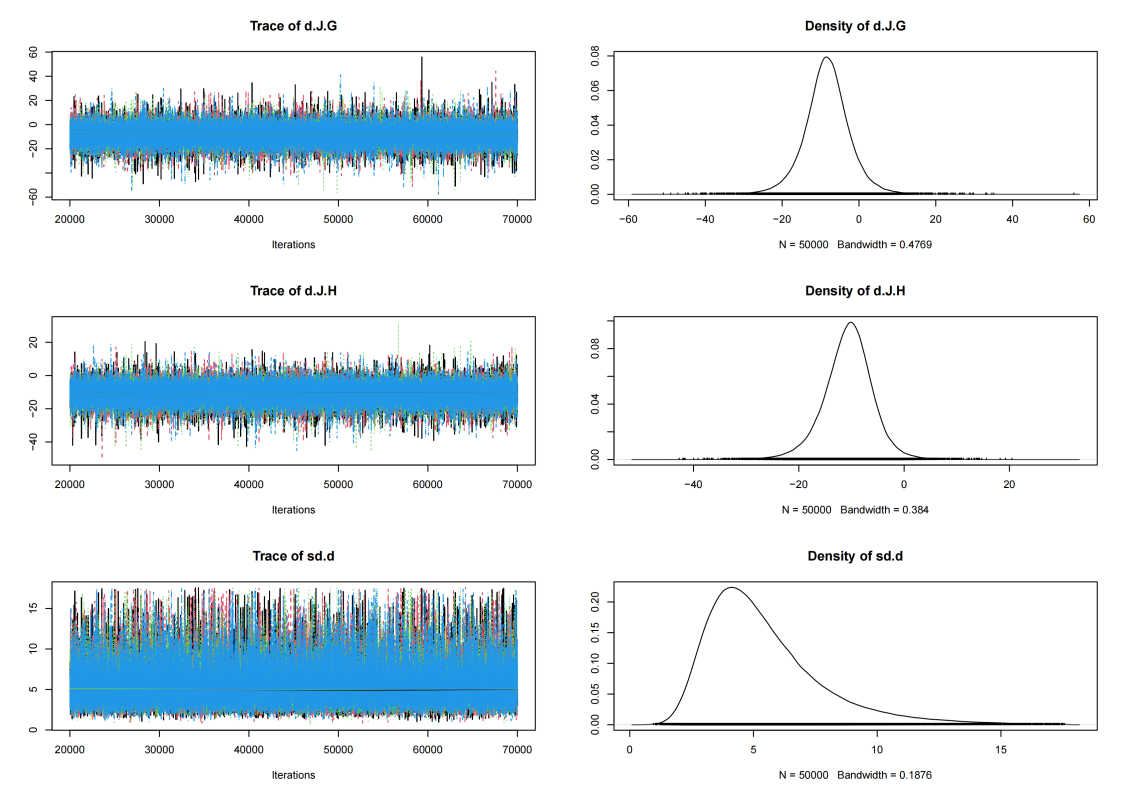
**

**Figure S6.12:** Density plots and Trajectory plots of of **NO**

**
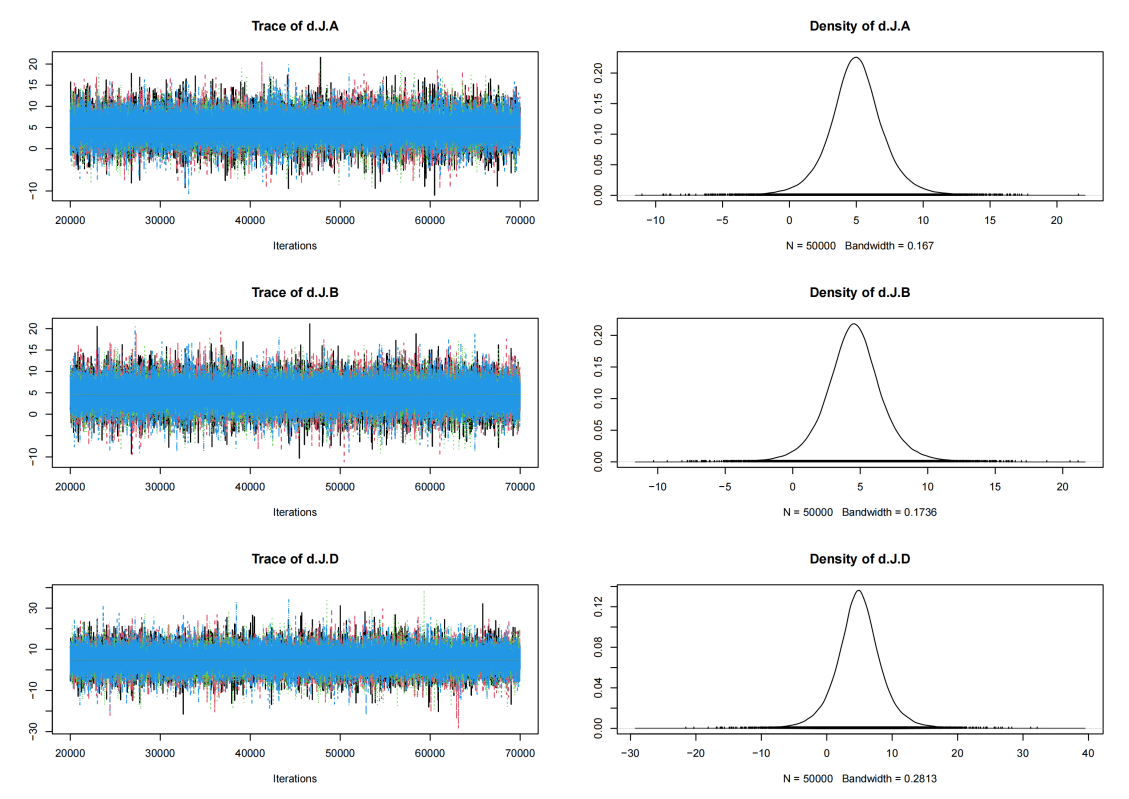

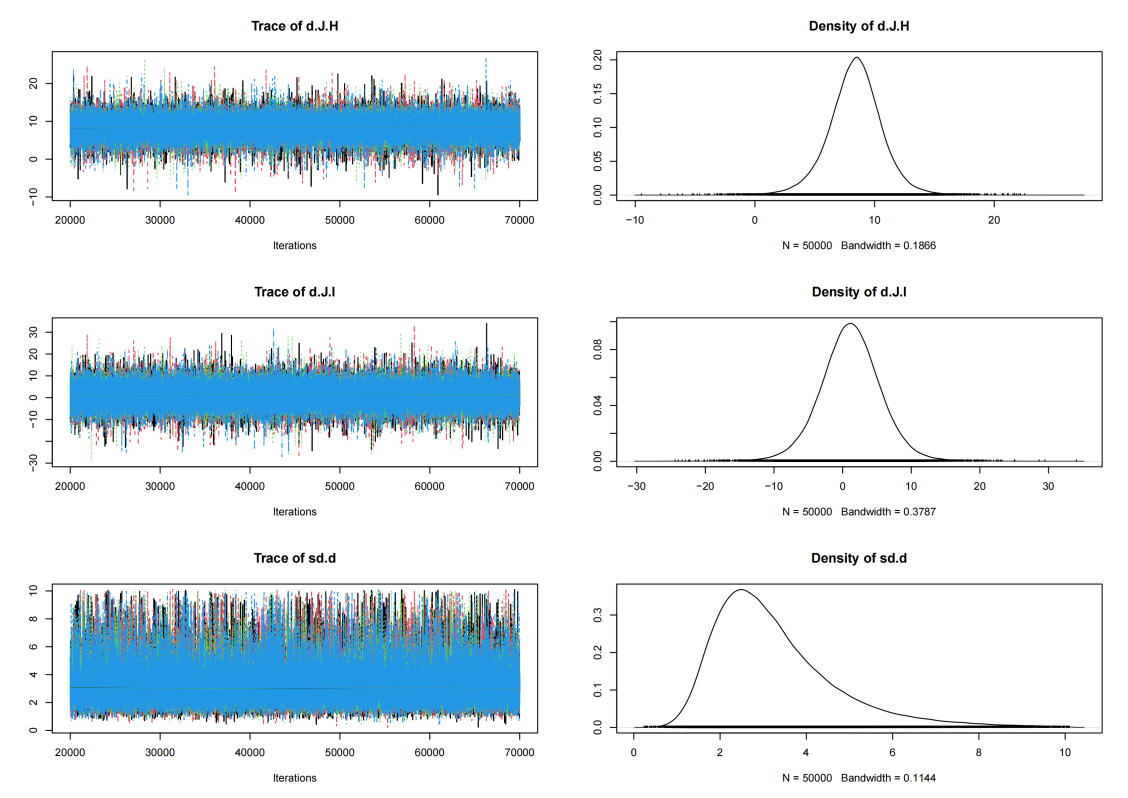
**

**Figure S6.13:** Density plots and Trajectory plots of of **ET-1**

**
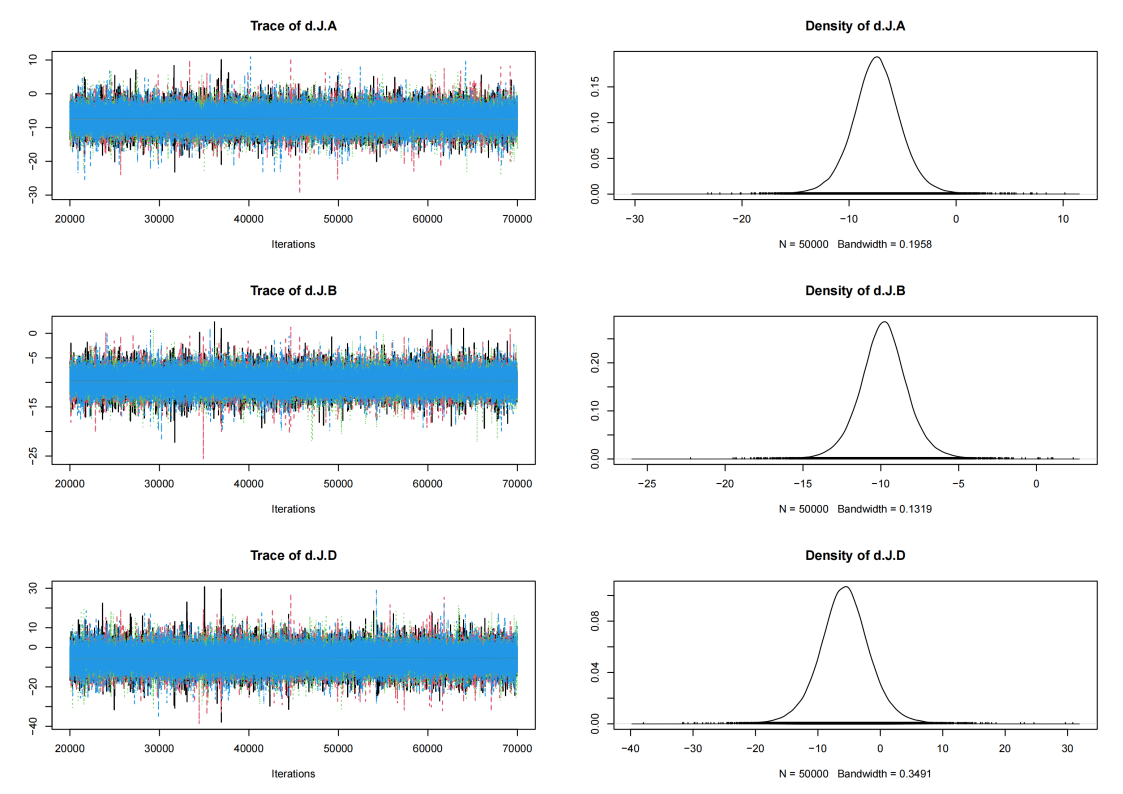

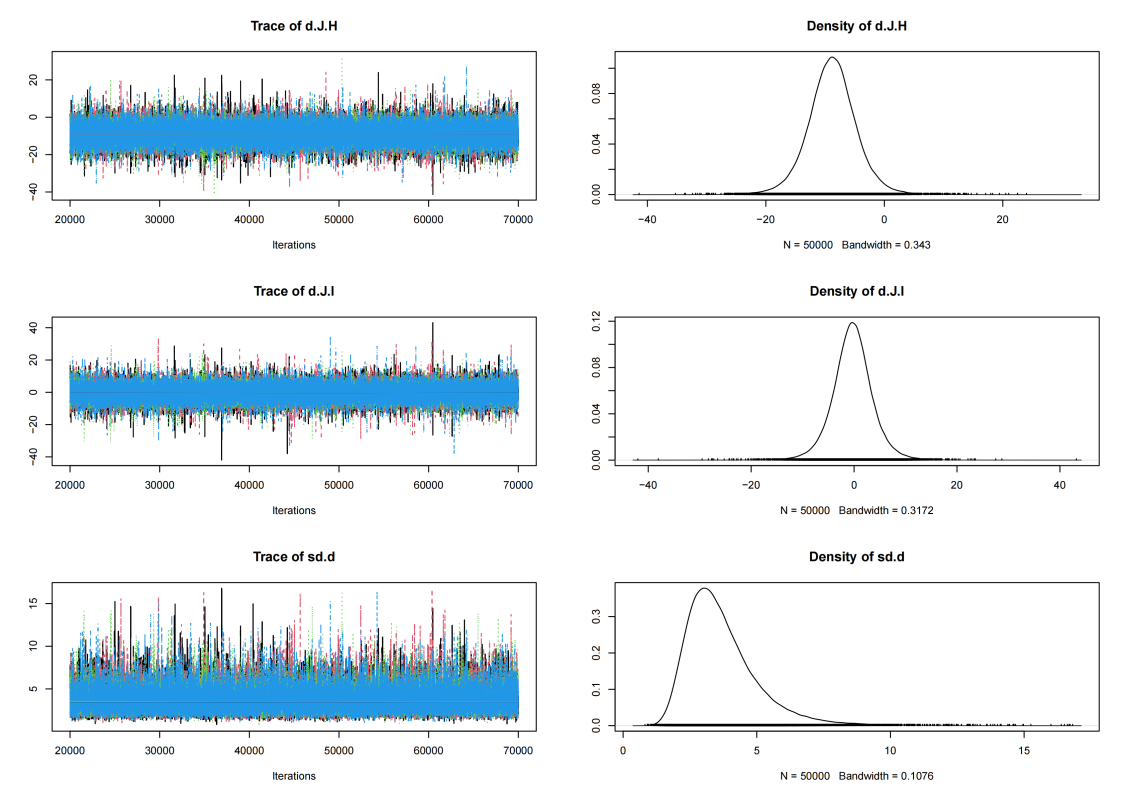
**

**Figure S6.14:** Density plots and Trajectory plots of of **Adverse** **events**

**
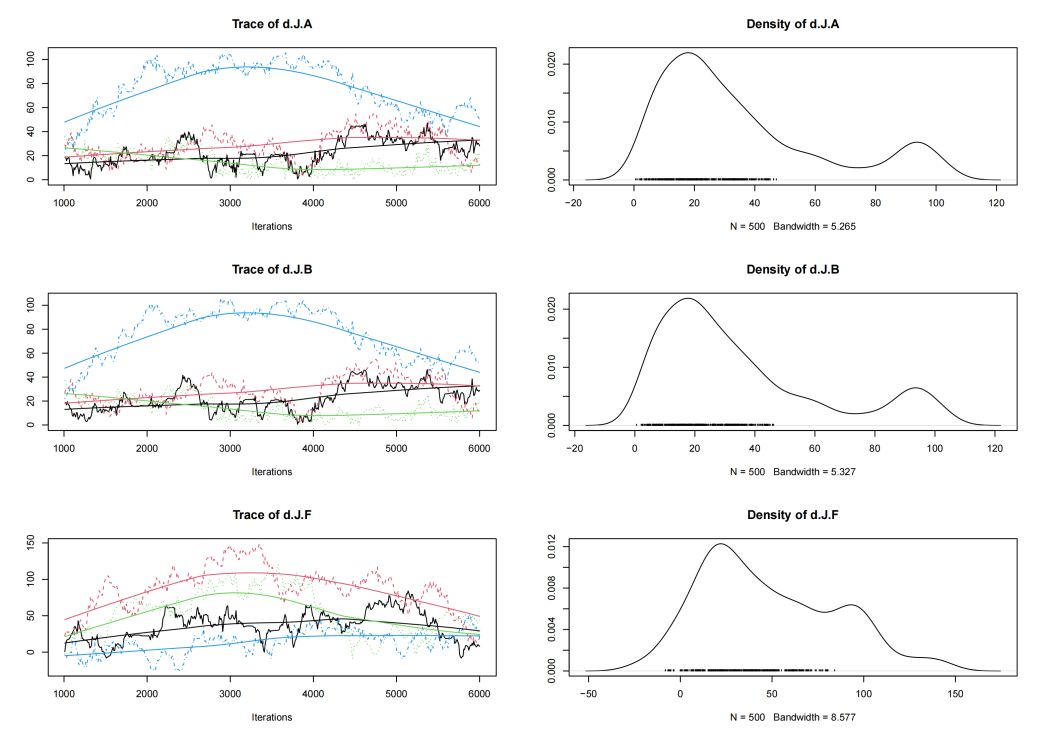

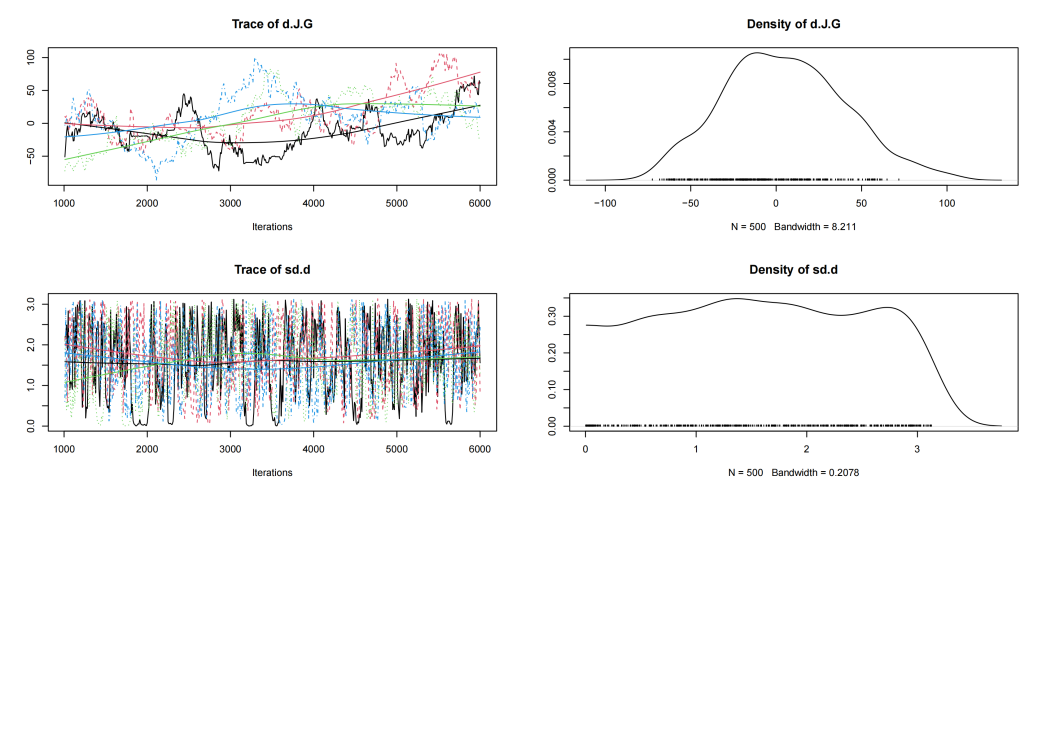
**

**Appendix 7:** Convergence Diagnostic Plots of comparisons of each outcome

**Figure S7.1:** Convergence Diagnostic Plots of **sPAP**

**
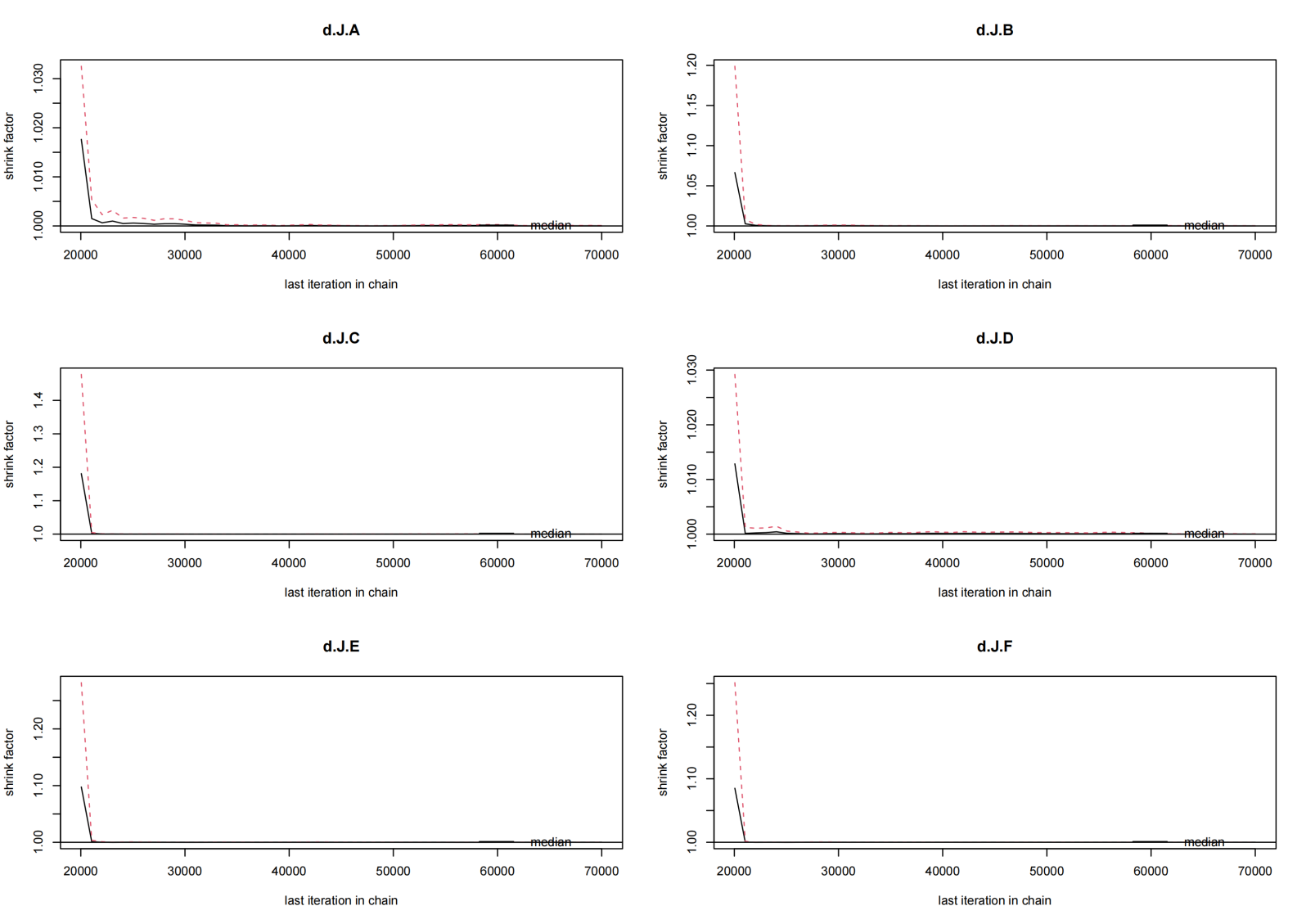
**

**
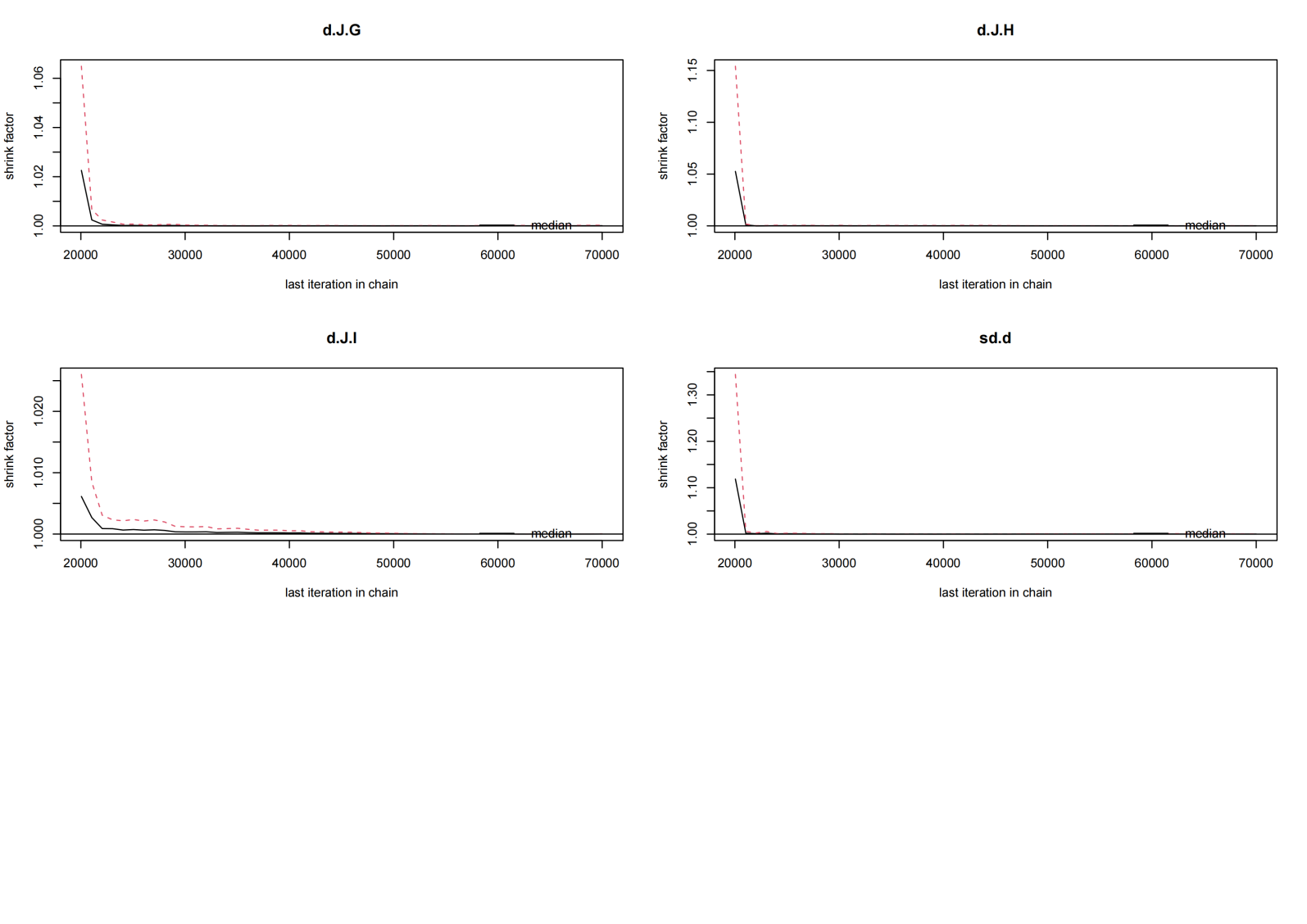
**

**Figure S7.2:** Convergence Diagnostic Plots of **mPAP**

**
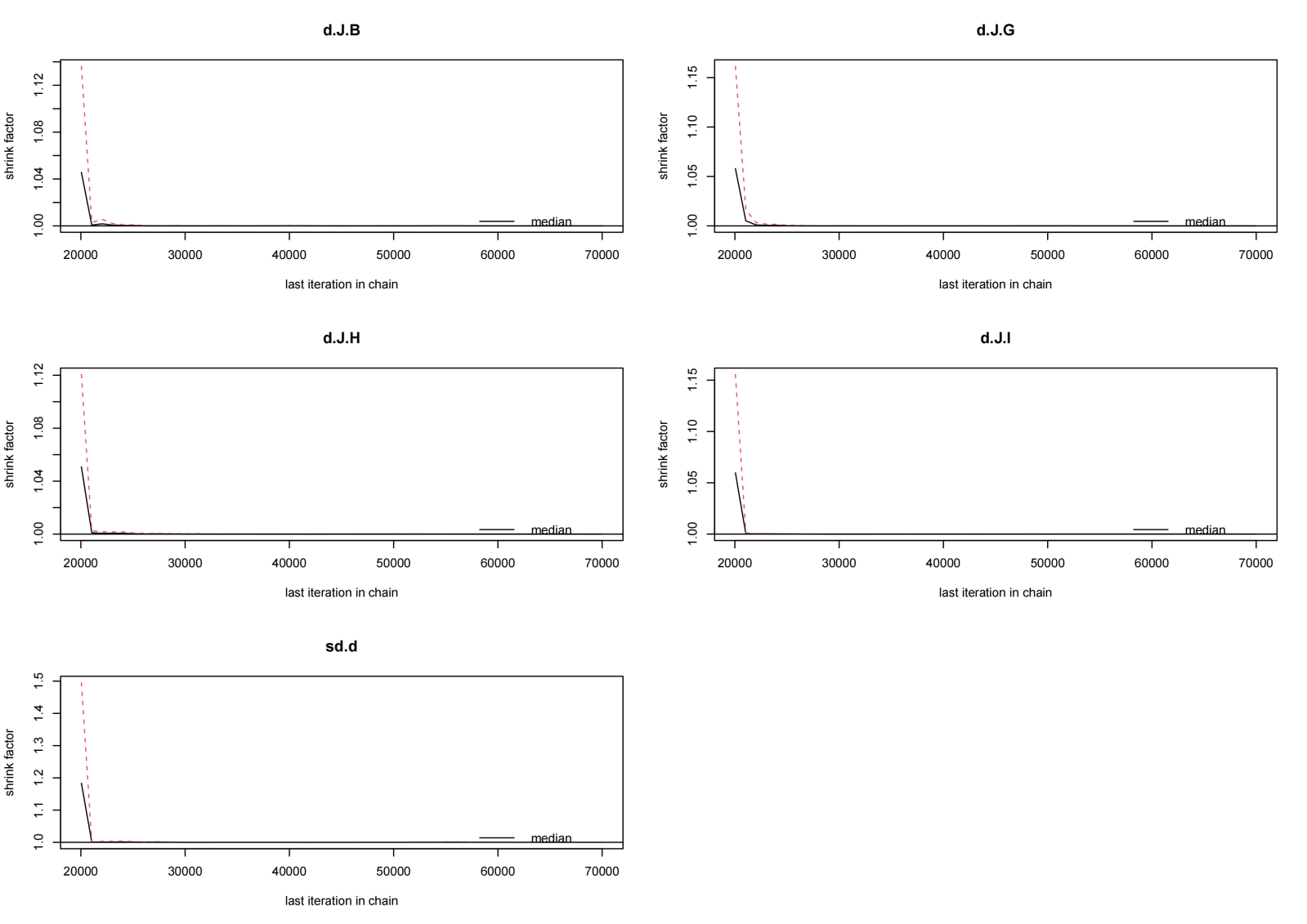
**

**Figure S7.3:** Convergence Diagnostic Plots of **6MWD**

**
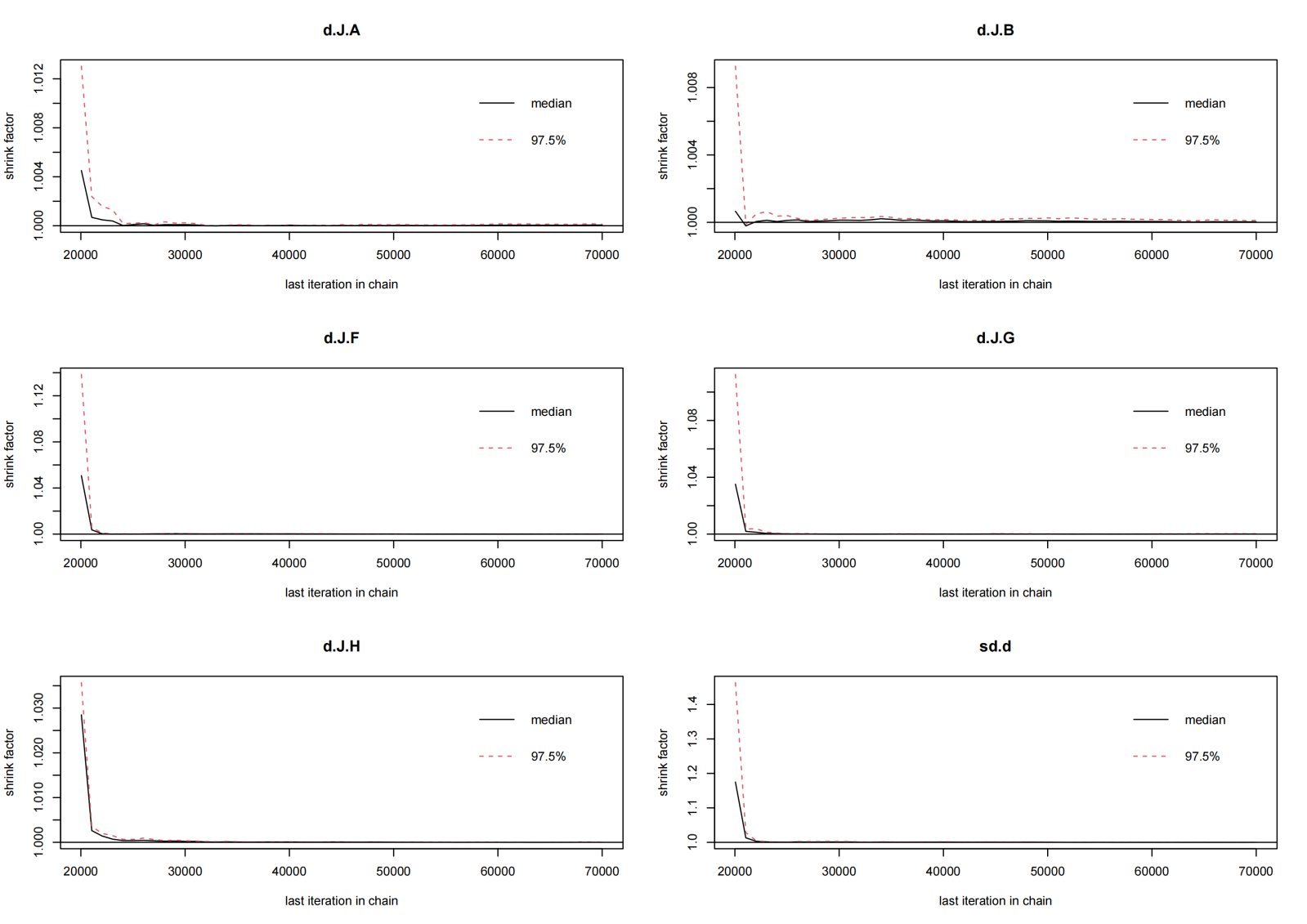
**

**Figure S7.4:** Convergence Diagnostic Plots of **FVC**

**
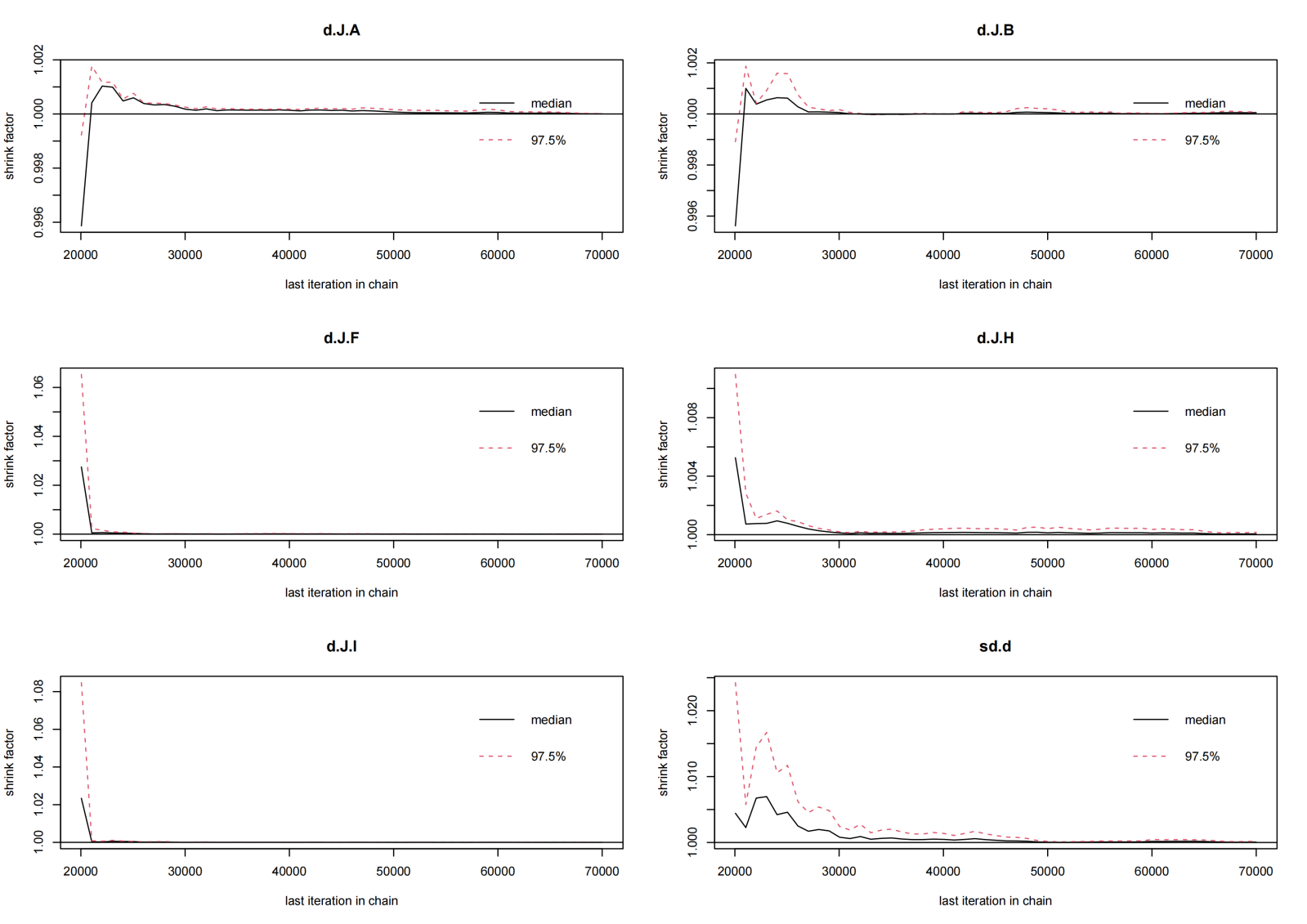
**

**Figure S7.5:** Convergence Diagnostic Plots of **FEV1**

**
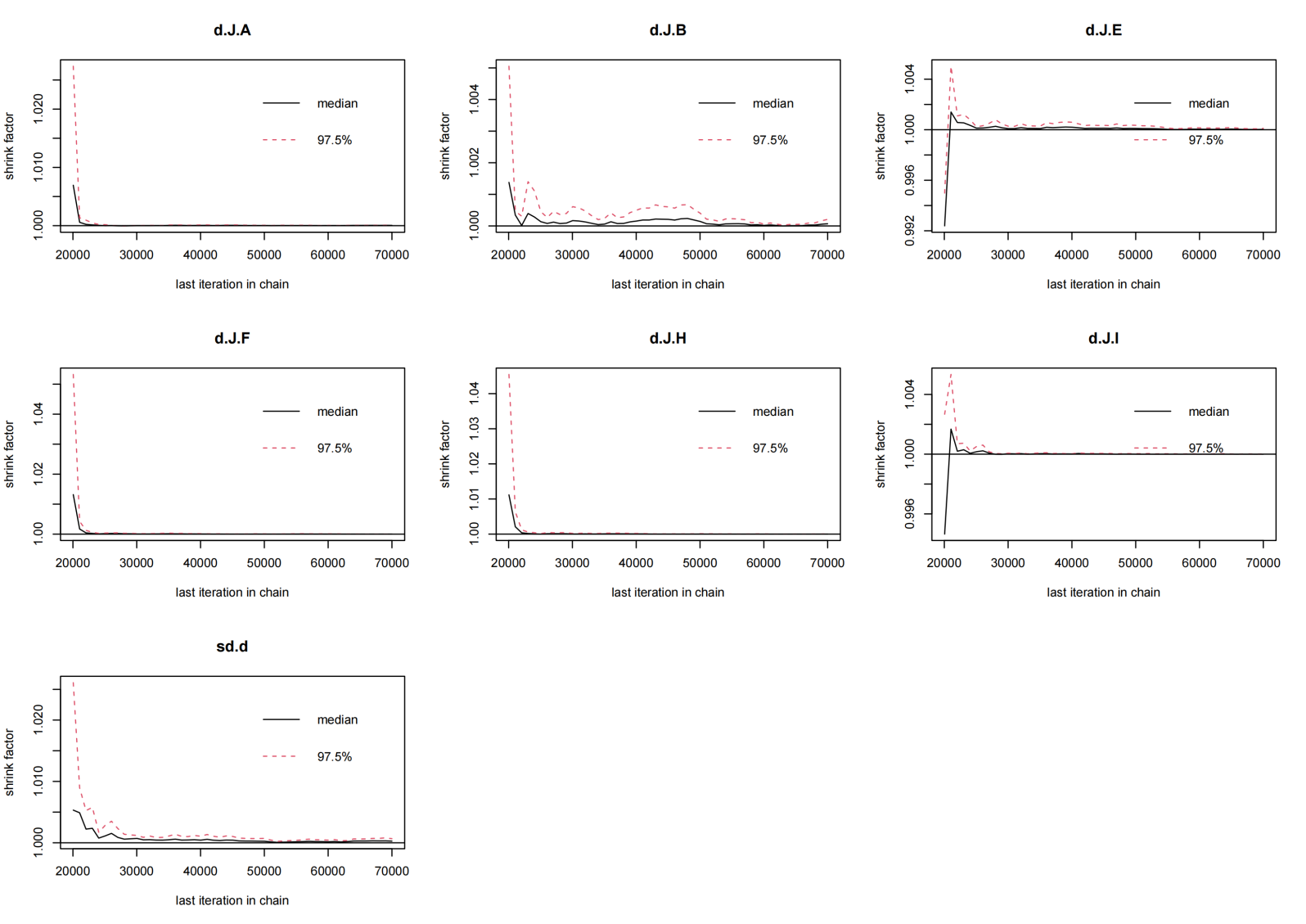
**

**Figure S7.6:** Convergence Diagnostic Plots of **FEV1/FVC**

**
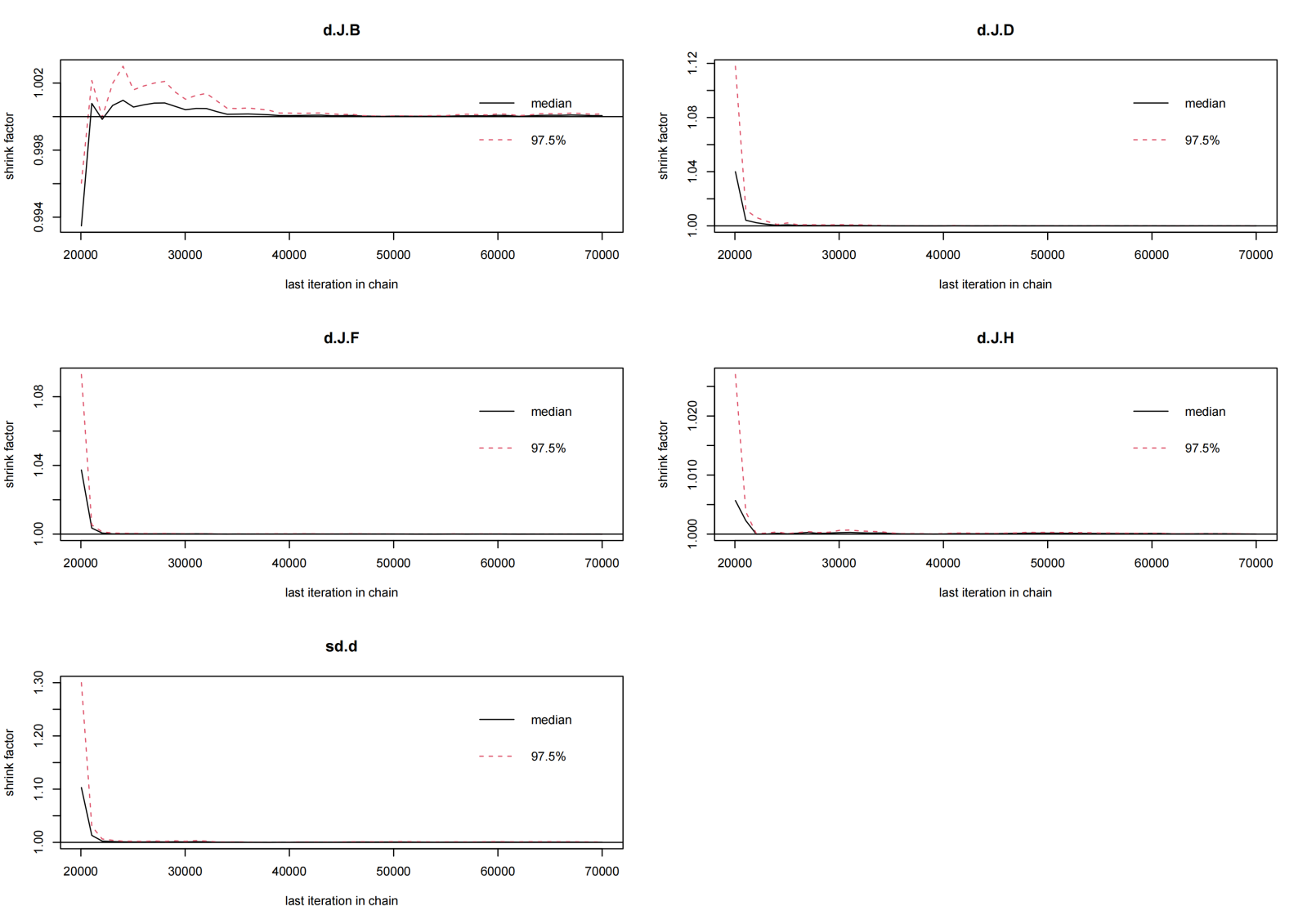
**

**Figure S7.7:** Convergence Diagnostic Plots of **PO2**

**
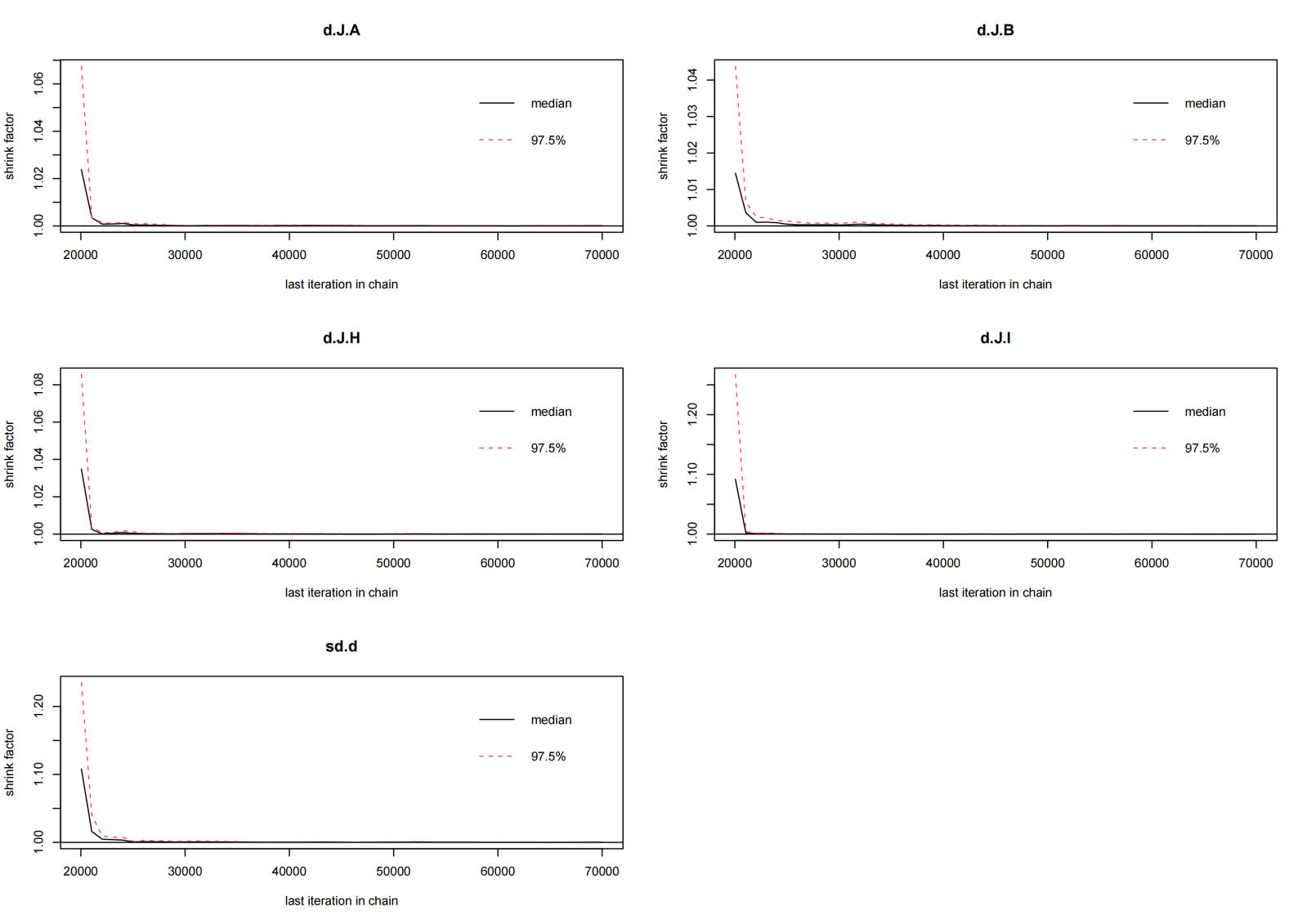
**

**Figure S7.8:** Convergence Diagnostic Plots of **PCO2**

**
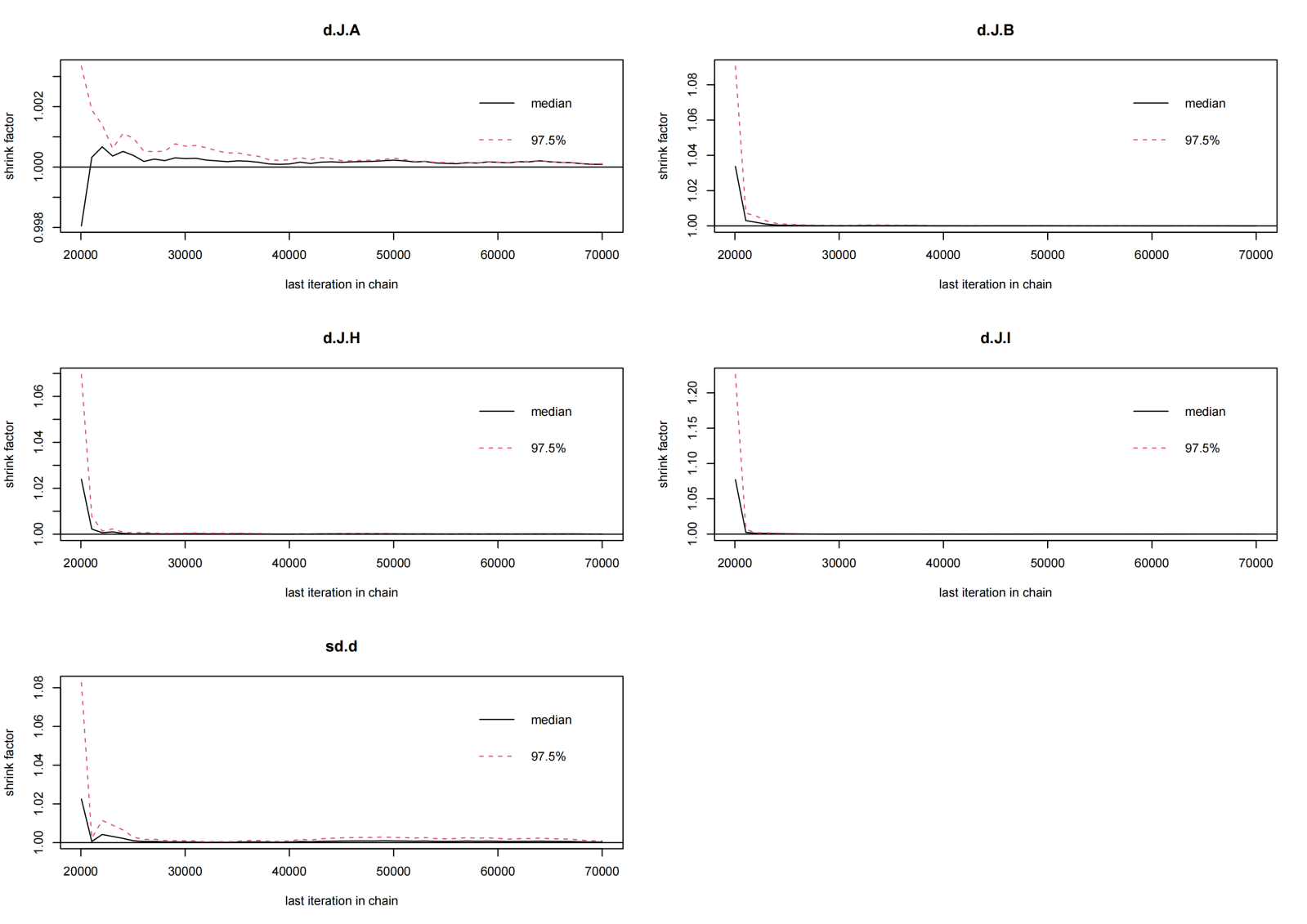
**

**Figure S7.9:** Convergence Diagnostic Plots of **TNF-α**

**
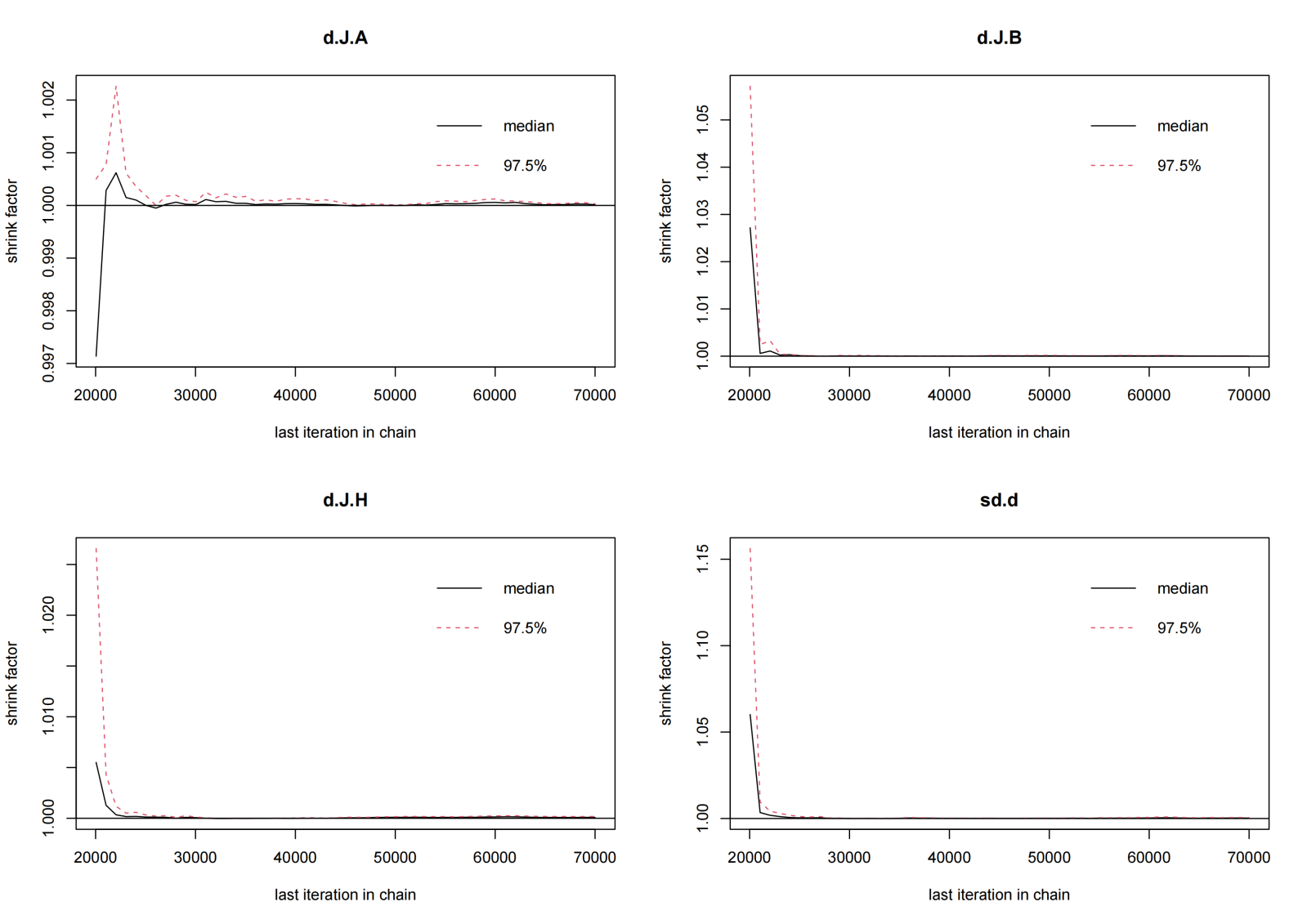
**

**Figure S7.10:** Convergence Diagnostic Plots of **hs-CRP**

**
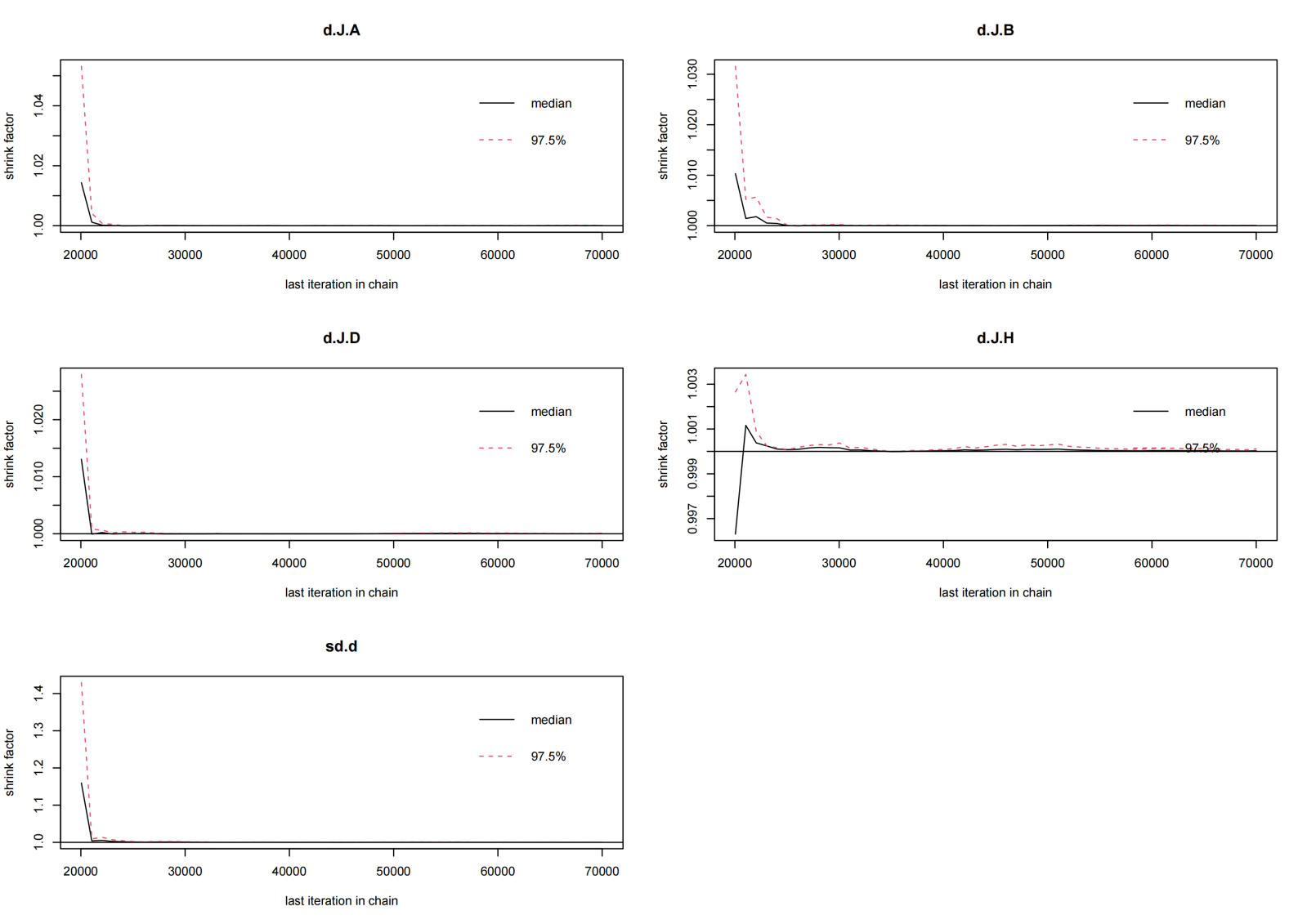
**

**Figure S7.11:** Convergence Diagnostic Plots of **IL-6**

**
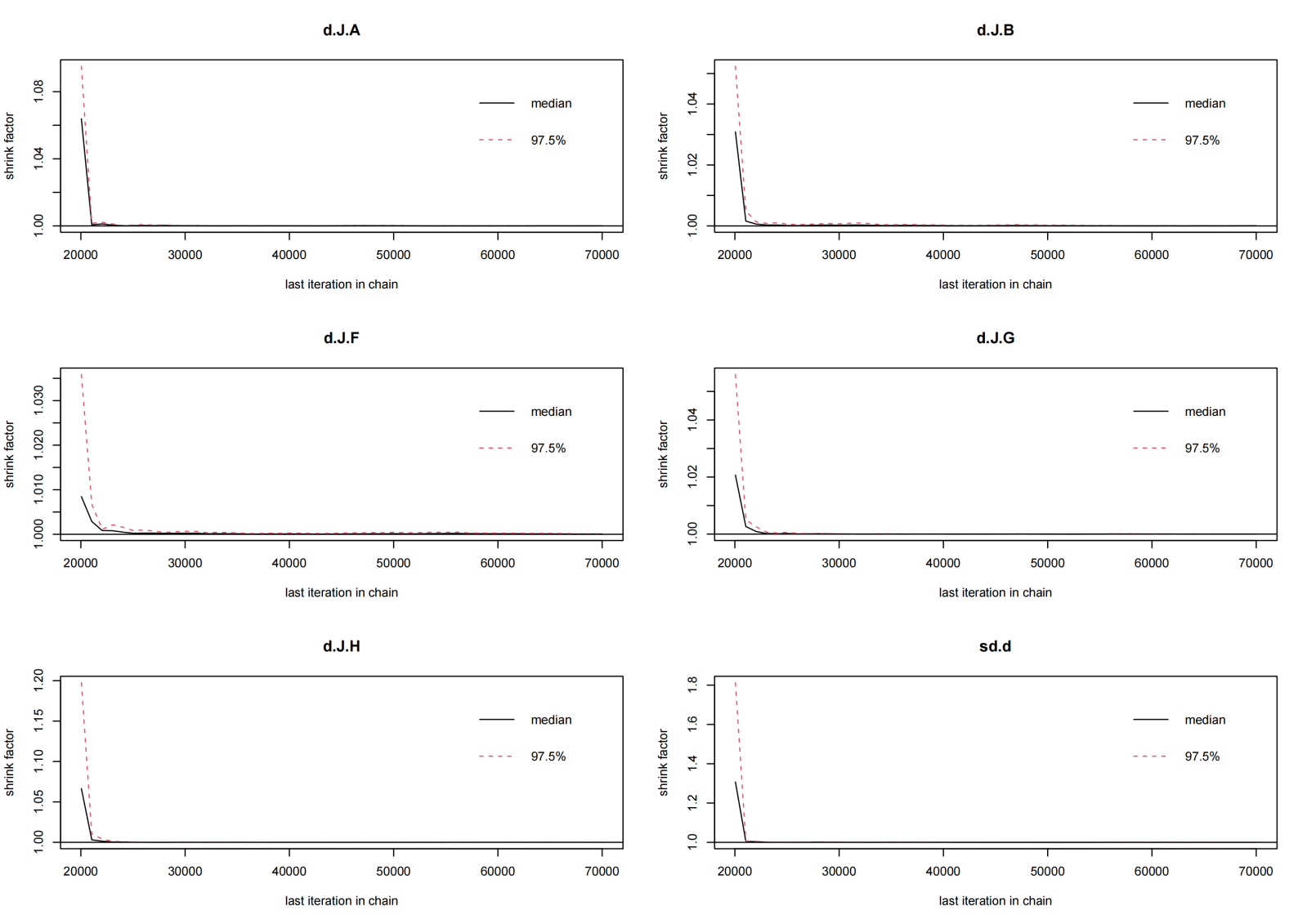
**

**Figure S7.12:** Convergence Diagnostic Plots of **NO**

**
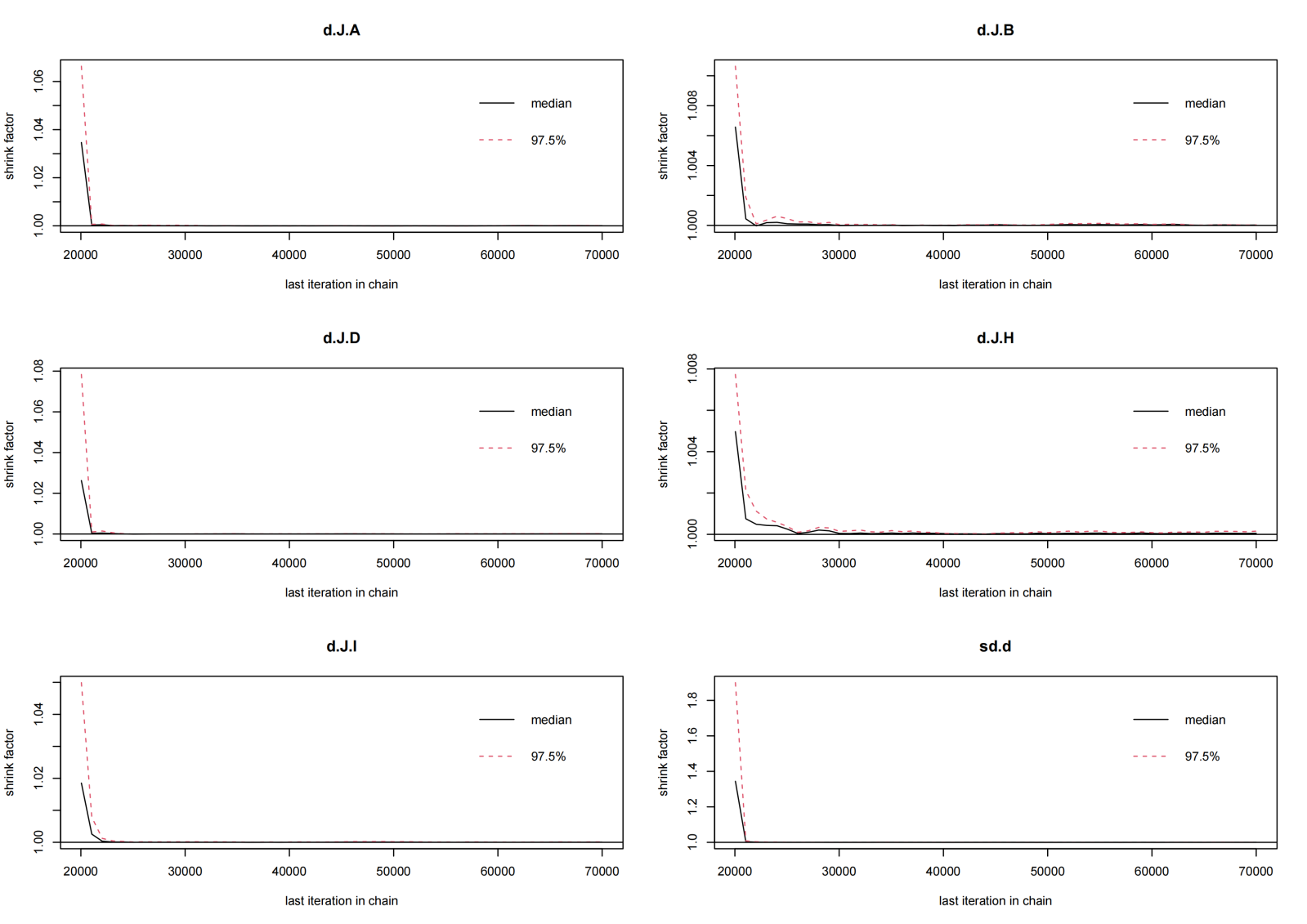
**

**Figure S7.13:** Convergence Diagnostic Plots of **ET-1**

**
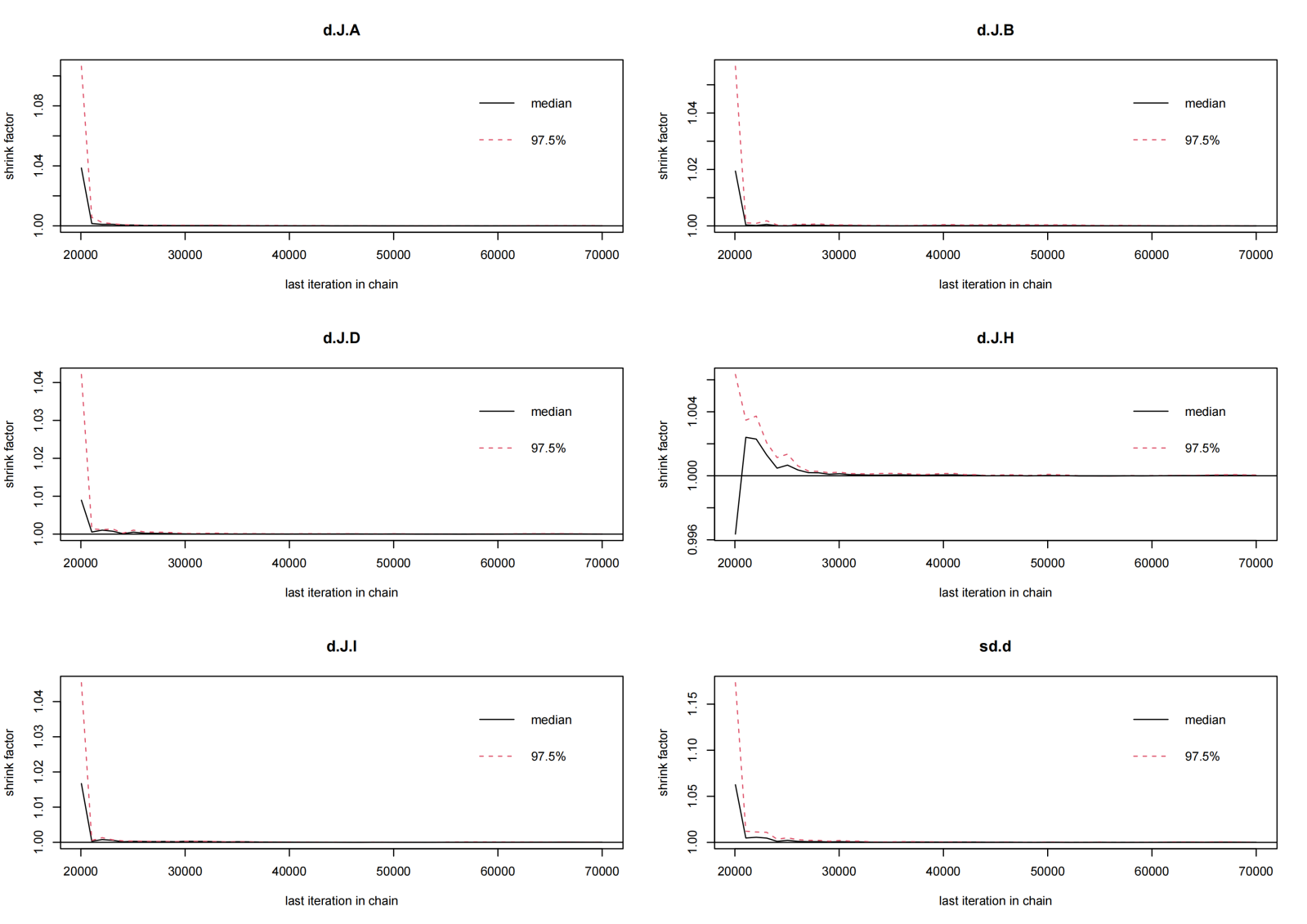
**

**Figure S7.14:** Convergence Diagnostic Plots of **Adverse** **events**

**
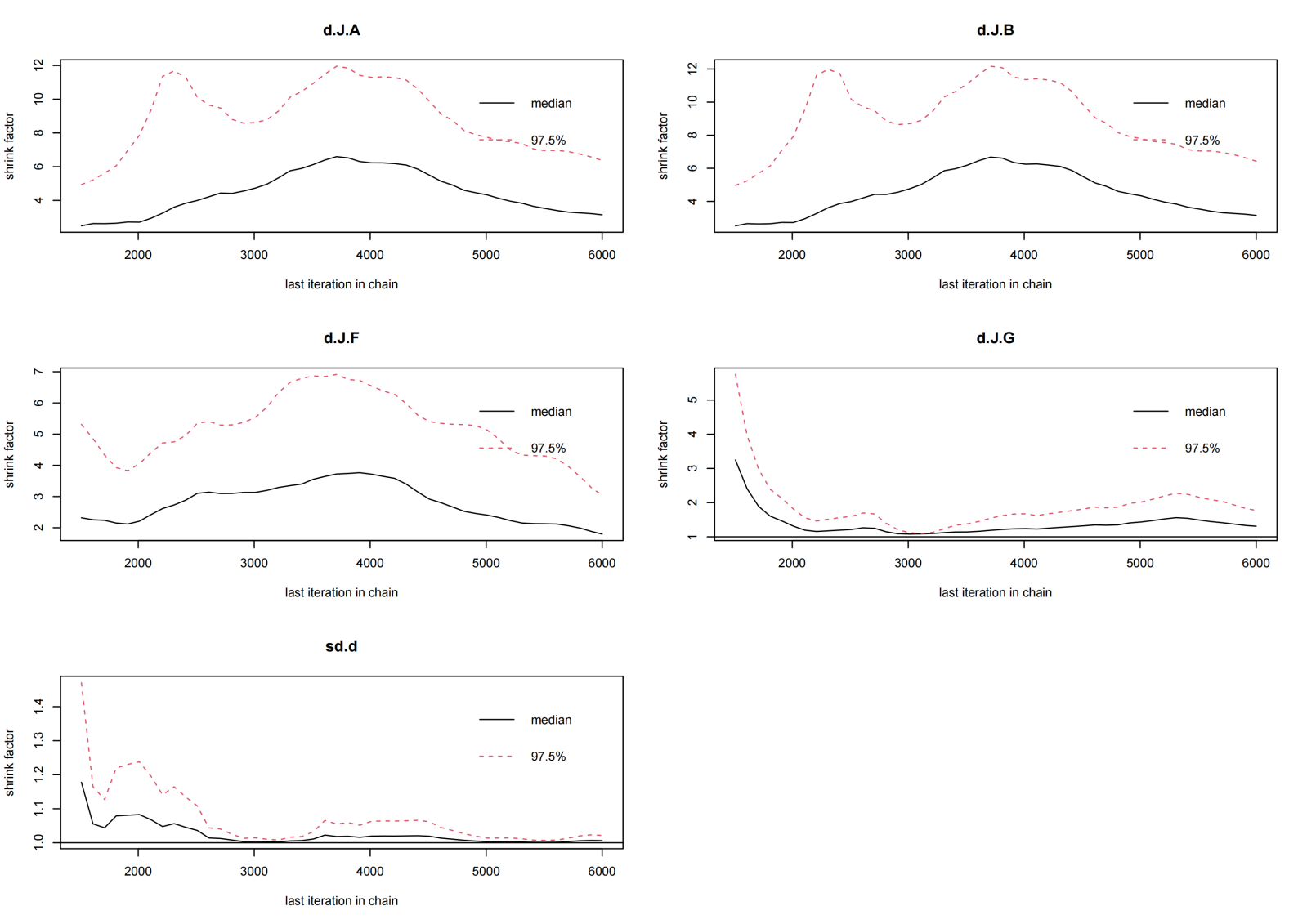
**

**Appendix 8: CINeMA Assessment**

We use the CINeMA framework to evidence certainty, assessing it for each network estimate based on the following criteria:

- **Within study bias:** We classified the overall risk of bias for each study as low risk of bias, the risk of bias as moderate when none of the four assessed risk of bias items were rated as high risk, and the risk of bias as high when one or both items were rated as high risk. See **Appendix 4** for the bias assessment. The risk of bias for a pairwise comparison of each drug is shown in **figure S8.1-8.2.**
- **Reporting bias:** We judged it visually by a funnel plot **(Appendix 9)**.
- **Indirectness:** Transferability assumptions were assessed by reporting the baseline sPAP in the included study population and by comparing age at baseline concordance between groups.
- **Imprecision:** We use the CINeMA website to grade the accuracy of each comparison.
- **Heterogeneity:** We assessed the degree of worry by comparing clinical reasoning based on 95% confidence intervals (CIs) while applying the same clinical reasoning framework as for inaccuracy. In particular, we judged the consistency of our findings based on the confidence and prediction intervals associated with clinically important effect sizes. And we used the same thresholds of clinical significance as described above and followed the recommendations automatically provided by CINeMA (https://cinema.ispm.unibe.ch/).

**Figure S8.1:** Risk of bias contribution by intervention group in **sPAP**


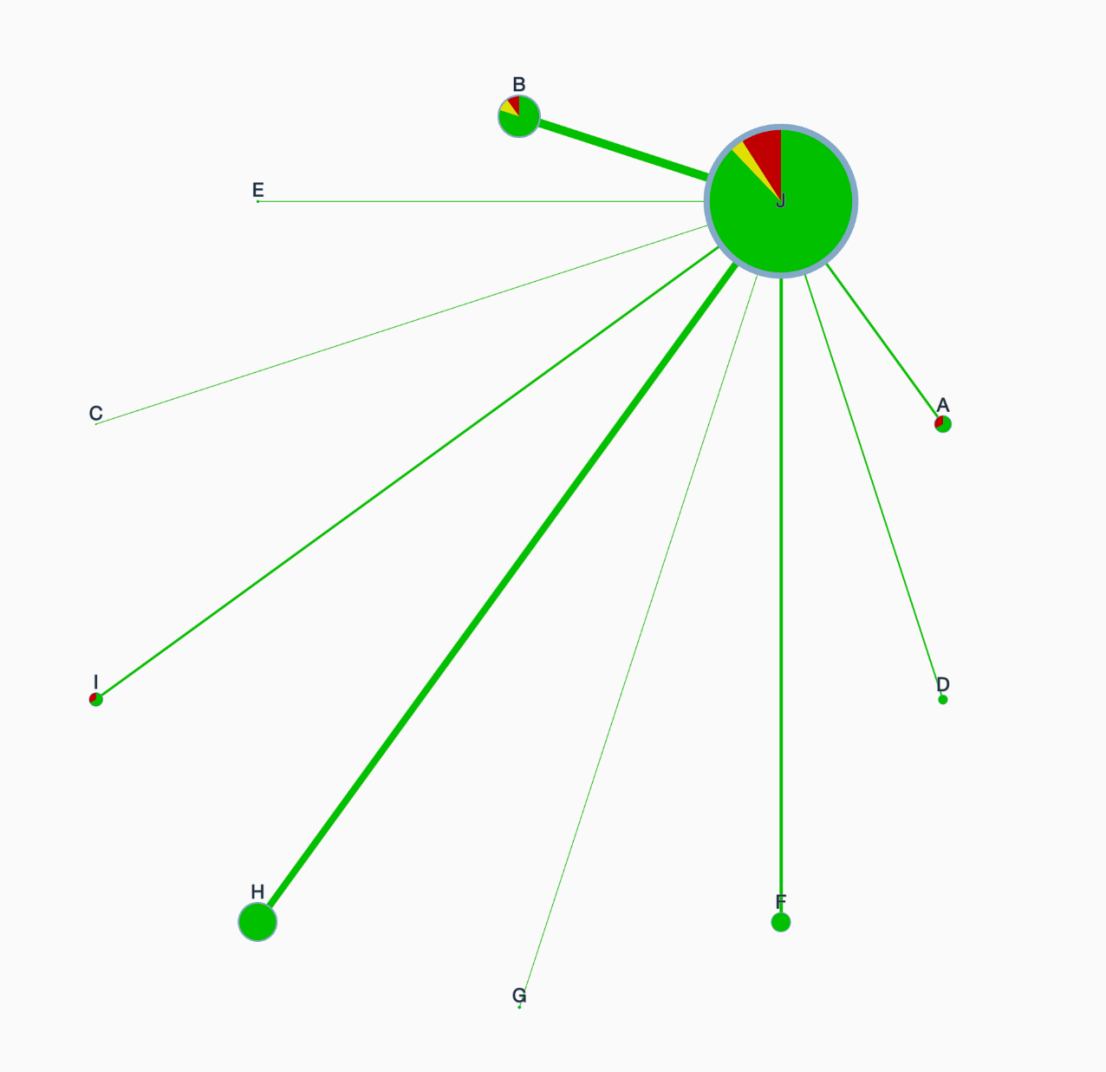


A, Atorvastatin 10mg+ST; B, Atorvastatin 20mg+ST; C, Atorvastatin 40mg+ST; D, Fluvastatin 40mg+ST; E, Pravastatin 40mg; F, Rosuvastatin 10mg+ST; G, Rosuvastatin 20mg+ST; H, Simvastatin 20mg+ST; I, Simvastatin 40mg+ST; J, ST.

**Figure S8.2:** Overall risk of bias by treatment comparison in **sPAP**


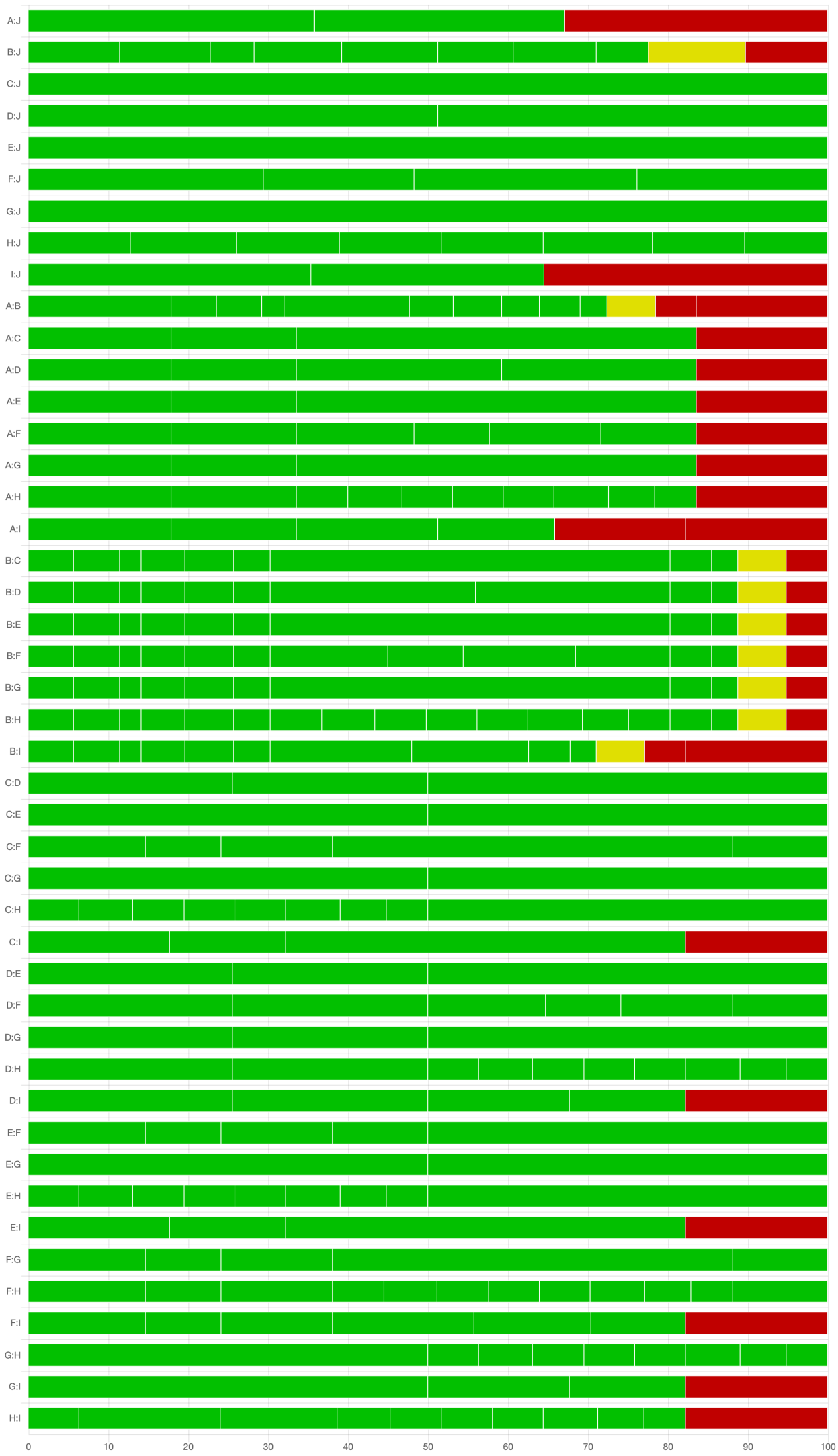


**Table S8.1:** Transitivity (Indirectness) Assessment

| Study | Design | Baseline variable | | | Randomised treatments |
| --- | --- | --- | --- | --- | --- |
|  |  | Gender ratio | Age years | PH/sPAP |  |
| Cao 2018 | RCT | 17 : 13 | 70.46 ± 5.02 | 57.69 ± 8.02 | Atorvastatin+ST：30 |
|  |  | 17 : 13 | 71.03 ± 4.91 | 56.79 ± 7.64 | ST：30 |
| Qu 2012 | RCT | 42 : 8 | 70.52 ± 9.63 | 58.33 ± 8.95 | Atorvastatin+ST：50 |
|  |  | 44 : 6 | 71.29 ± 11.14 | 56.04 ± 8.51 | ST：50 |
| He 2019 | RCT | 25 : 18 | 68.52 ± 6.21 | NA | Atorvastatin+ST：43 |
|  |  | 27 : 16 | 69.48 ± 5.52 |  | ST：43 |
| Yu 2012 | RCT | 57 : 21 | 65.154 ± 6.6 | 50.3 ± 8.4 | Atorvastatin+ST：78 |
|  |  | 61 : 17 | 64.895 ± 6.7 | 51.1 ± 8.2 | ST：78 |
| Luo 2013 | RCT | 21 : 9 | 72 ± 11 | 32.2 ± 6.4 | Atorvastatin+ST：30 |
|  |  | 20 : 10 | 73 ± 10 | 31.3 ± 5.8 | ST：30 |
| Wang 2015 | RCT | 27 : 12 | 66.4 ± 6.3 | 56.46 ± 4.23 | Atorvastatin+ST：39 |
|  |  | 29 : 10 | 66.0 ± 6.1 | 55.68 ± 7.70 | ST：39 |
| Yan 2018 | RCT | 21 : 11 | 64.9 ± 4.3 | 52.78 ± 5.42 | Atorvastatin+ST：32 |
|  |  | 19 : 13 | 63.3 ± 3.9 | 53.12 ± 5.67 | ST：32 |
| Mao 2017 | RCT | 32 : 21 | 51.28 ± 3.12 | >36 | Atorvastatin+ST：53 |
|  |  | 30 : 23 | 52.42 ± 3.29 |  | ST：53 |
| Zhang 2019 | RCT | 32 : 28 | 46.3 ± 15.1 | 63.37 ± 23.27 | Atorvastatin+ST：60 |
|  |  | 29 : 31 | 45.6 ± 14.9 | 62.43 ± 22.95 | ST：60 |
| Zhang 2013 | RCT | NA | 52.41 ± 7.75 | >36 | Atorvastatin+ST：49 |
|  |  |  | 51.47 ± 7.63 |  | ST：49 |
| Wu 2014 | RCT | NA | NA | NA | Atorvastatin+ST：30 |
|  |  |  |  |  | Atorvastatin+ST：30 |
|  |  |  |  |  | ST：30 |
| Deng 2015 | RCT | 25 : 15 | 63.13 ± 3.1 | >30 | Atorvastatin+ST：40 |
|  |  | 24 : 16 | 62.33 ± 3.1 |  | ST：40 |
| Liu 2016 | RCT | 19 : 21 | 71.07 ± 6.78 | NA | Atorvastatin+ST：40 |
|  |  | 23 : 17 | 70.07 ± 8.78 |  | ST：40 |
| Li 2012 | RCT | NA | NA | NA | Atorvastatin+ST：35 |
|  |  |  |  |  | ST：35 |
| Sun 2020 | RCT | 29 : 21 | 56.5 ± 3.3 | NA | Atorvastatin+ST：35 |
|  |  | 27 : 23 | 57.2 ± 4.4 |  | ST：35 |
| Chen 2016 | RCT | 30 : 5 | 66 ± 4.0 | 50.2 ± 8.6 | Atorvastatin+ST：35 |
|  |  | 37 : 4 | 67 ± 4.8 | 51.8 ± 6.2 | ST：41 |
| Jiang 2015 | RCT | 38 : 26 | 58.9 ± 8.7 | 52.3 ± 7.6 | Atorvastatin+ST：35 |
|  |  | 40 : 24 | 58.7 ± 8.5 | 51.2 ± 7.9 | ST：41 |
| Niu 2015 | RCT | 26 : 24 | 45.34 ± 14.46 | 52.26 ± 12.16 | Atorvastatin+ST：50 |
|  |  | 27 : 23 | 45.22 ± 14.29 | 51.32 ± 11.84 | ST：50 |
| Wang 2011 | RCT | 24 : 11 | 64 ± 3.5 | 53.2 ± 4.8 | Fluvastatin+ST：35 |
|  |  | 22 : 13 | 63 ± 5.2 | 52.8 ± 4.6 | ST：35 |
| Wang 2012 | RCT | 32 : 24 | 62.4 ± 7.3 | 48.9 ± 8.4 | Fluvastatin+ST：56 |
|  |  | 33 : 23 | 68.4 ± 8.5 | 48.2 ± 7.6 | ST：56 |
| Xu 2020 | RCT | 28 : 24 | 65.42 ± 6.14 | 52.19 ± 6.18 | Rosuvastatin+ST：52 |
|  |  | 30 : 26 | 65.31 ± 6.23 | 52.04 ± 6.23 | ST：56 |
| Tang 2018 | RCT | 14 : 16 | 69.84 ± 7.07 | 52.08 ± 11.7 | Rosuvastatin+ST：30 |
|  |  | 13 : 17 | 68.12 ± 7.19 | 50.41 ± 10.6 | ST：30 |
| Ren 2018 | RCT | 38 : 32 | 57.42 ± 11.31 | 68.63 ± 8.26 | Rosuvastatin+ST：70 |
|  |  | 39 : 31 | 57.23 ± 11.29 | 68.39 ± 8.52 | ST：70 |
| Nan 2016 | RCT | NA | NA | 55 ± 7 | Rosuvastatin+ST：70 |
|  |  |  |  | 57 ± 6 | ST：70 |
| Ye 2015 | RCT | 23 : 7 | 58.5 ± 7.9 | 37.91 ± 4.36 | Simvastatin+ST：30 |
|  |  | 25 : 5 | 59.4 ± 6.8 | 37.98 ± 4.45 | ST：30 |
| Rang 2013 | RCT | 17 : 13 | 73 ± 10 | 36.2 ± 0.8 | Simvastatin+ST：30 |
|  |  | 18 : 12 | 72 ± 8 | 36.3 ± 0.8 | ST：30 |
| Xia 2013 | RCT | 84 : 16 | 69.0 ± 8.0 | 55.3 ± 8.6 | Simvastatin+ST：50 |
|  |  |  |  | 54.7 ± 7.3 | ST：50 |
| Chen 2017 | RCT | 25 : 25 | 63.35 ± 4.26 | 31.73 ± 4.80 | Simvastatin+ST：50 |
|  |  | 28 : 22 | 63.20 ± 4.89 | 32.76 ± 4.83 | ST：50 |
| Ding 2016 | RCT | 28 : 22 | 70.29 ± 7.86 | 65.46 ± 5.81 | Simvastatin+ST：50 |
|  |  | 27 : 23 | 70.15 ± 7.62 | 65.35 ± 5.87 | ST：50 |
| Tang 2017 | RCT | 26 : 17 | 61.7 ± 9.4 | 65.15 ± 5.12 | Simvastatin+ST：43 |
|  |  | 28 : 15 | 60.8 ± 8.9 | 64.74 ± 4.86 | ST：43 |
| Zhang 2015 | RCT | 31 : 14 | 69.88 ± 6.84 | >40 | Simvastatin+ST：45 |
|  |  | 33 : 12 | 65.48 ± 6.13 |  | ST：45 |
| Hu 2019 | RCT | 32 : 23 | 62.9 ± 5.8 | 56.19 ± 6.26 | Simvastatin+ST：55 |
|  |  | 30 : 25 | 63.3 ± 6.0 | 56.14 ± 6.29 | ST：55 |
| Tong 2016 | RCT | 40 : 25 | 64.5 ± 8.5 | 37.15 ± 4.23 | Simvastatin+ST：65 |
|  |  | 41 : 24 | 64.2 ± 8.6 | 36.25 ± 4.42 | ST：65 |
| Sun 2014 | RCT | 24 : 16 | 67.8 | 55.25 ± 8.01 | Simvastatin+ST：40 |
|  |  | 26 : 14 | 68.2 | 56.41 ± 7.54 | ST：40 |
| Yan 2012 | RCT | 31 : 21 | 68 ± 10 | 35.5 ± 0.3 | Simvastatin+ST：52 |
|  |  | 29 : 23 | 67 ± 9 | 35.5 ± 0.4 | ST：52 |
| Liu 2010 | RCT | 52 : 10 | 67 ± 4.0 | 51.1 ± 8.2 | Simvastatin+ST：52 |
|  |  |  |  | 50.3 ± 8.4 | ST：52 |
| Arian 2017 | RCT | 10 : 6 | 65.8 ± 11.5 | 47.9 ± 15.4 | Atorvastatin+ST：16 |
|  |  | 13 : 6 | 63.7 ± 7.6 | 49.2 ± 16.3 | ST：18 |
| Chogtu 2016 | RCT | NA | 61.4 ± 8.4 | NA | Rosuvastatin+ST：32 |
|  |  |  | 65.9 ± 9.7 |  | ST：30 |
| Lee 2009 | RCT | 20 : 7 | 71 ± 8 | 47 ± 8 | Pravastatin: 27 |
|  |  | 19 : 7 | 72 ± 6 | 47 ± 7 | ST: 26 |
| Liu 2013 | RCT | 20 : 13 | 66.2 ± 7.4 | 52.7 ± 8.1 | Atorvastatin+ST：33 |
|  |  | 23 : 12 | 64.9 ± 8.2 | 51.7 ± 7.9 | ST：35 |
| Moosavi 2013 | RCT | 15 : 9 | 65.0 ± 11.0 | 48.5 ± 6.9 | Atorvastatin+ST：19 |
|  |  | 13 : 8 | 68.0 ± 14.0 | 49.7 ± 1.4 | ST：17 |

**Table S8.2:** CINeMA Results of **sPAP**

| Comparison | Number of studies | Within-study bias | Reporting bias | Indirectness | Imprecision | Heterogeneity | Incoherence | Confidence rating |
| --- | --- | --- | --- | --- | --- | --- | --- | --- |
| A:J | 3 | No concerns | Low risk | No concerns | No concerns | No concerns | Major concerns | High |
| B:J | 10 | No concerns | Low risk | No concerns | No concerns | Major concerns | Major concerns | Moderate |
| C:J | 1 | No concerns | Low risk | No concerns | Major concerns | No concerns | Major concerns | Low |
| D:J | 2 | No concerns | Low risk | No concerns | No concerns | Major concerns | Major concerns | Moderate |
| E:J | 1 | No concerns | Low risk | No concerns | Major concerns | No concerns | Major concerns | Low |
| F:J | 4 | No concerns | Low risk | No concerns | No concerns | No concerns | Major concerns | High |
| G:J | 1 | No concerns | Low risk | No concerns | Major concerns | No concerns | Major concerns | Low |
| H:J | 8 | No concerns | Low risk | No concerns | No concerns | Major concerns | Major concerns | Moderate |
| I:J | 3 | No concerns | Low risk | No concerns | Major concerns | No concerns | Major concerns | Low |
| A:B | 0 | No concerns | Low risk | No concerns | Major concerns | No concerns | Major concerns | Low |
| A:C | 0 | No concerns | Low risk | No concerns | Major concerns | No concerns | Major concerns | Low |
| A:D | 0 | No concerns | Low risk | No concerns | Major concerns | No concerns | Major concerns | Low |
| A:E | 0 | No concerns | Low risk | No concerns | Major concerns | No concerns | Major concerns | Low |
| A:F | 0 | No concerns | Low risk | No concerns | Major concerns | No concerns | Major concerns | Low |
| A:G | 0 | No concerns | Low risk | No concerns | Major concerns | No concerns | Major concerns | Low |
| A:H | 0 | No concerns | Low risk | No concerns | Major concerns | No concerns | Major concerns | Low |
| A:I | 0 | No concerns | Low risk | No concerns | Major concerns | No concerns | Major concerns | Low |
| B:C | 0 | No concerns | Low risk | No concerns | Major concerns | No concerns | Major concerns | Low |
| B:D | 0 | No concerns | Low risk | No concerns | Major concerns | No concerns | Major concerns | Low |
| B:E | 0 | No concerns | Low risk | No concerns | Major concerns | No concerns | Major concerns | Low |
| B:F | 0 | No concerns | Low risk | No concerns | Major concerns | No concerns | Major concerns | Low |
| B:G | 0 | No concerns | Low risk | No concerns | Major concerns | No concerns | Major concerns | Low |
| B:H | 0 | No concerns | Low risk | No concerns | Major concerns | No concerns | Major concerns | Low |
| B:I | 0 | No concerns | Low risk | No concerns | Major concerns | No concerns | Major concerns | Low |
| C:D | 0 | No concerns | Low risk | No concerns | Major concerns | No concerns | Major concerns | Low |
| C:E | 0 | No concerns | Low risk | No concerns | Major concerns | No concerns | Major concerns | Low |
| C:F | 0 | No concerns | Low risk | No concerns | Major concerns | No concerns | Major concerns | Low |
| C:G | 0 | No concerns | Low risk | No concerns | Major concerns | No concerns | Major concerns | Low |
| C:H | 0 | No concerns | Low risk | No concerns | Major concerns | No concerns | Major concerns | Low |
| C:I | 0 | No concerns | Low risk | No concerns | Major concerns | No concerns | Major concerns | Low |
| D:E | 0 | No concerns | Low risk | No concerns | Major concerns | No concerns | Major concerns | Low |
| D:F | 0 | No concerns | Low risk | No concerns | Major concerns | No concerns | Major concerns | Low |
| D:G | 0 | No concerns | Low risk | No concerns | Major concerns | No concerns | Major concerns | Low |
| D:H | 0 | No concerns | Low risk | No concerns | Major concerns | No concerns | Major concerns | Low |
| D:I | 0 | No concerns | Low risk | No concerns | Major concerns | No concerns | Major concerns | Low |
| E:F | 0 | No concerns | Low risk | No concerns | Major concerns | No concerns | Major concerns | Low |
| E:G | 0 | No concerns | Low risk | No concerns | Major concerns | No concerns | Major concerns | Low |
| E:H | 0 | No concerns | Low risk | No concerns | Major concerns | No concerns | Major concerns | Low |
| E:I | 0 | No concerns | Low risk | No concerns | Major concerns | No concerns | Major concerns | Low |
| F:G | 0 | No concerns | Low risk | No concerns | Major concerns | No concerns | Major concerns | Low |
| F:H | 0 | No concerns | Low risk | No concerns | Major concerns | No concerns | Major concerns | Low |
| F:I | 0 | No concerns | Low risk | No concerns | No concerns | Major concerns | Major concerns | Moderate |
| G:H | 0 | No concerns | Low risk | No concerns | Major concerns | No concerns | Major concerns | Low |
| G:I | 0 | No concerns | Low risk | No concerns | Major concerns | No concerns | Major concerns | Low |
| H:I | 0 | No concerns | Low risk | No concerns | Major concerns | No concerns | Major concerns | Low |

**Appendix 9: Funnel plots**

**Figure S9.1:** Funnel plot of **sPAP**

**
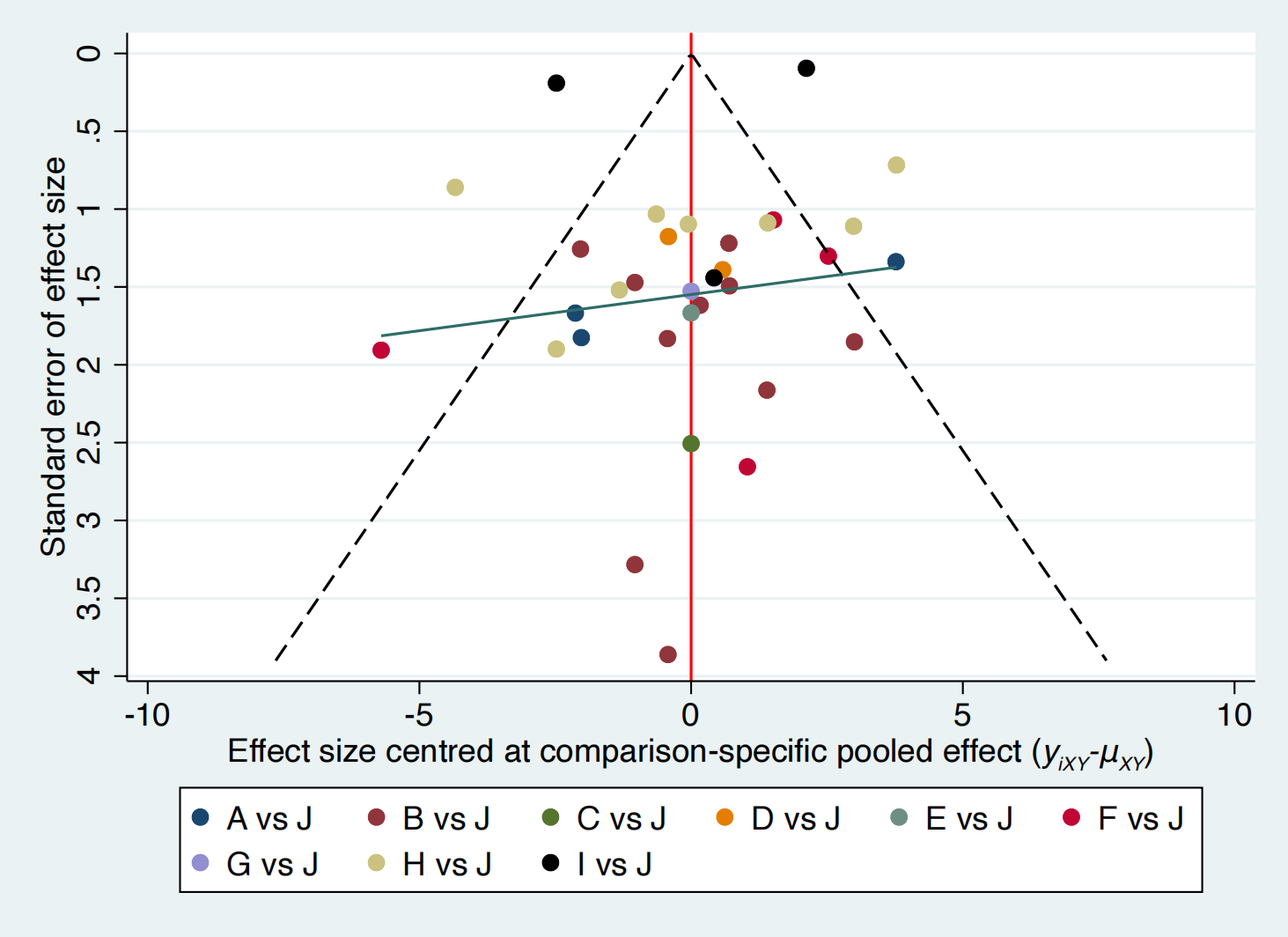
**

**Figure S9.2:** Funnel plot of **mPAP**

**
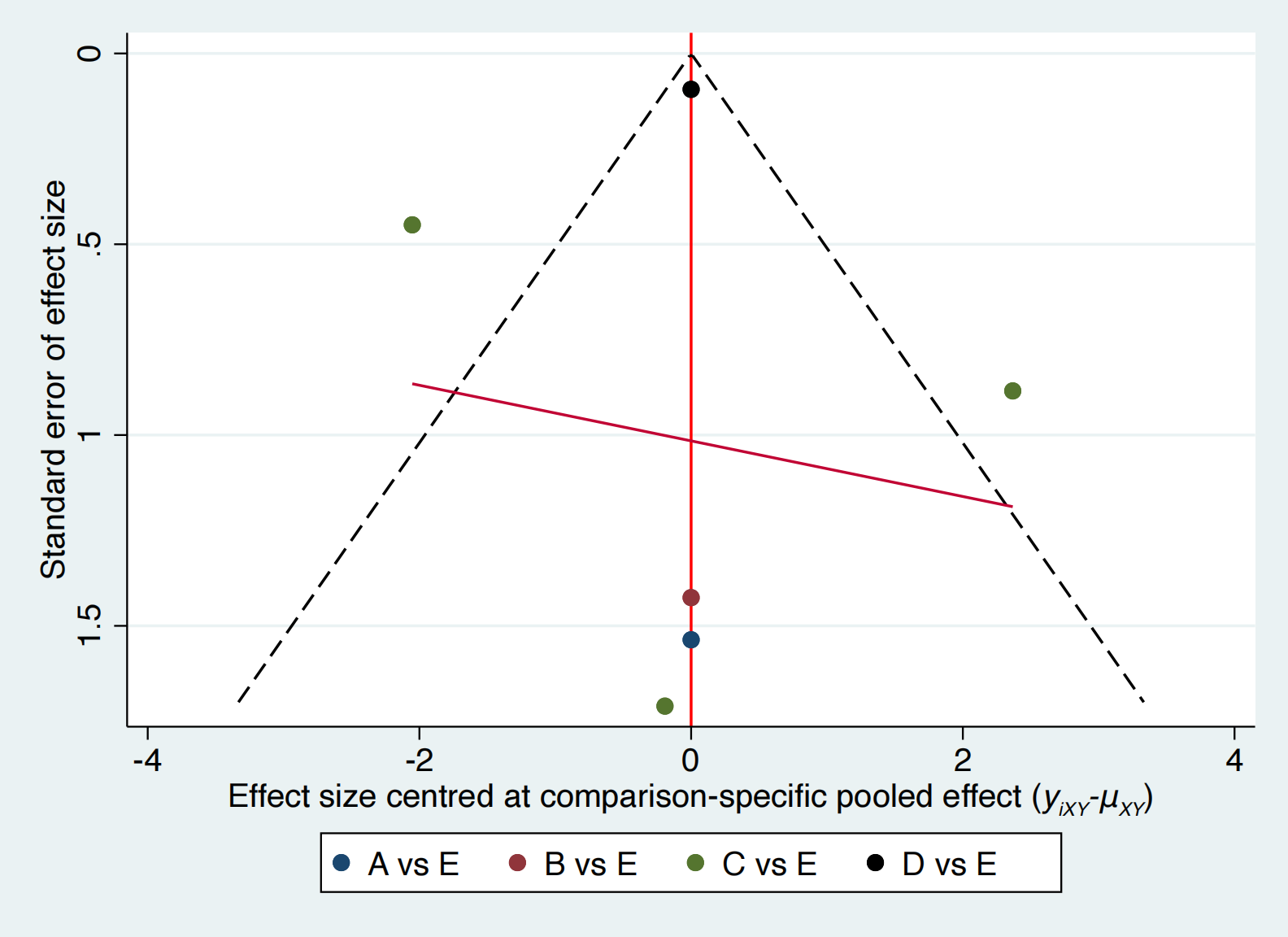
**

**Figure S9.3:** Funnel plot of **6MWD**

**
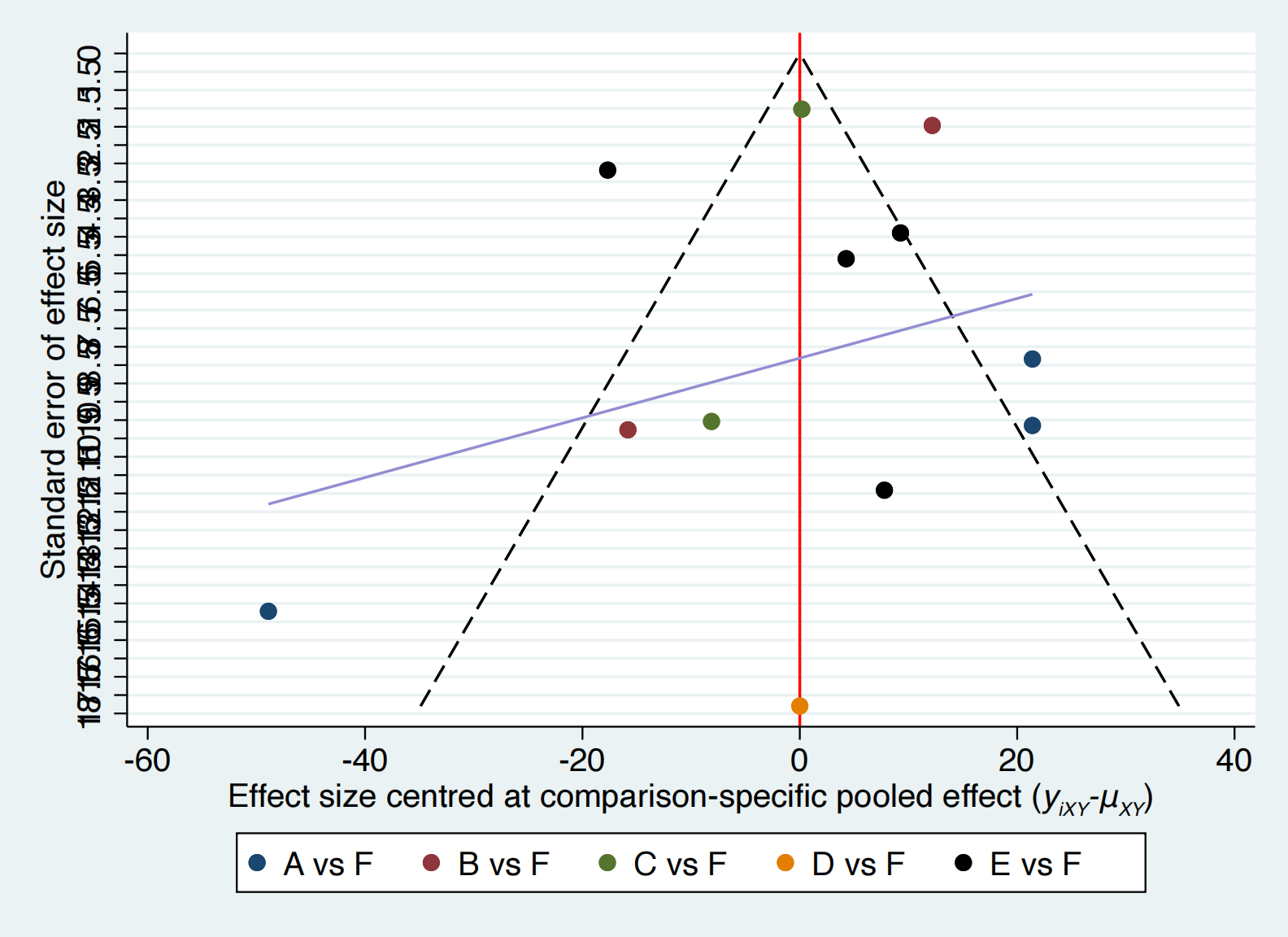
**

**Figure S9.4:** Funnel plot of **FVC**

**
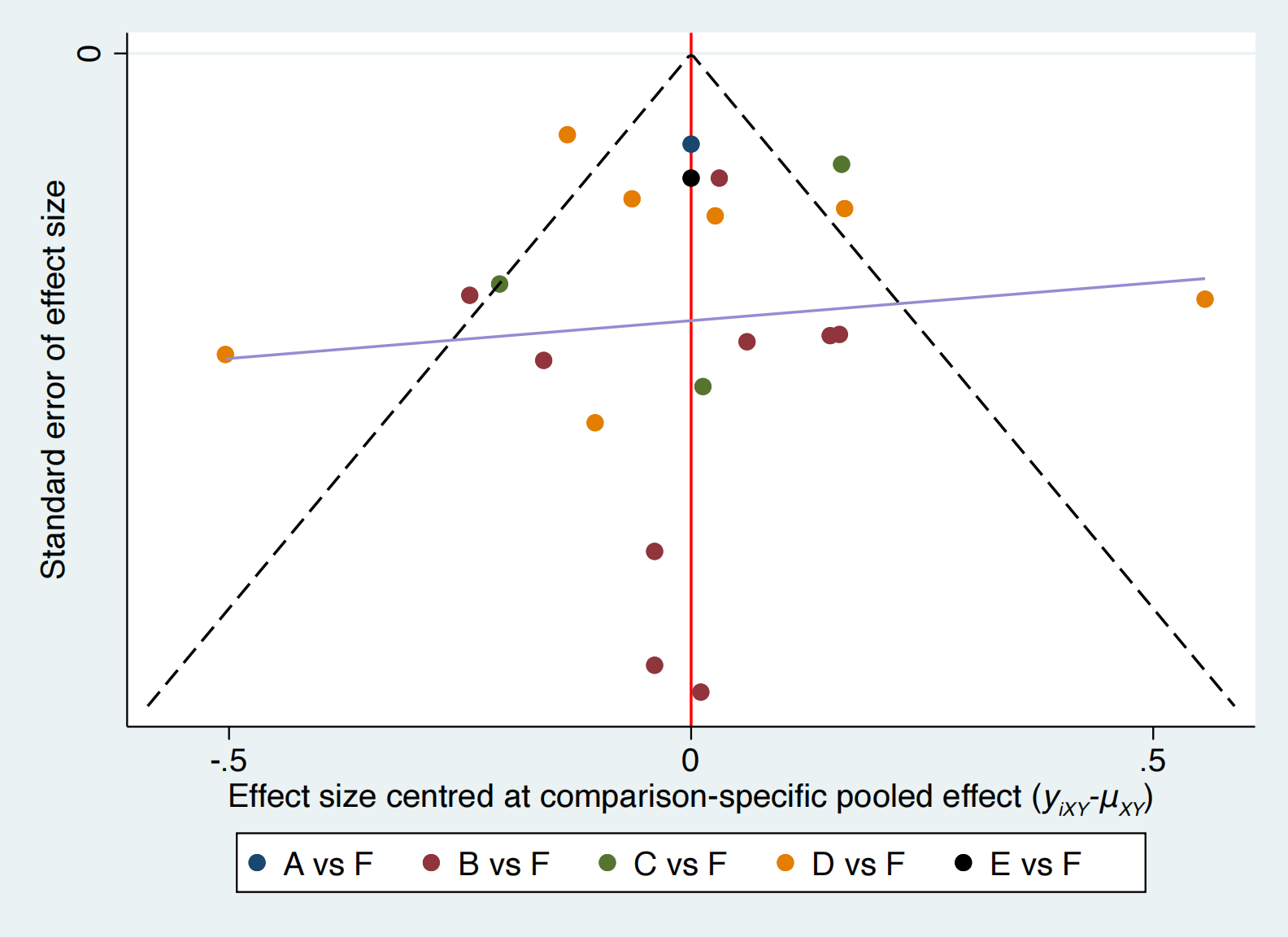
**

**Figure S9.5:** Funnel plot of **FEV1**

**
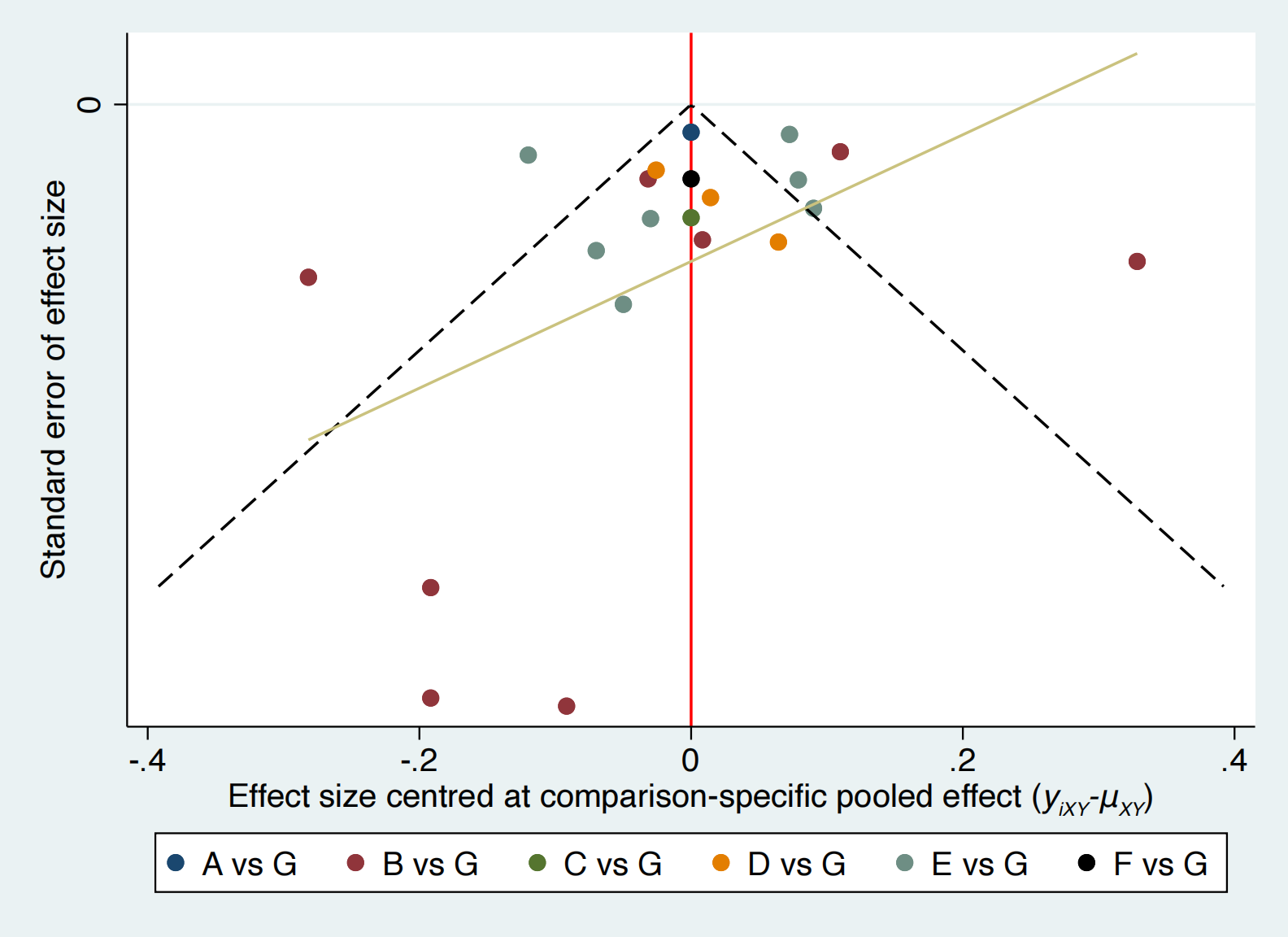
**

**Figure S9.6:** Funnel plot of **FEV1/FVC**

**
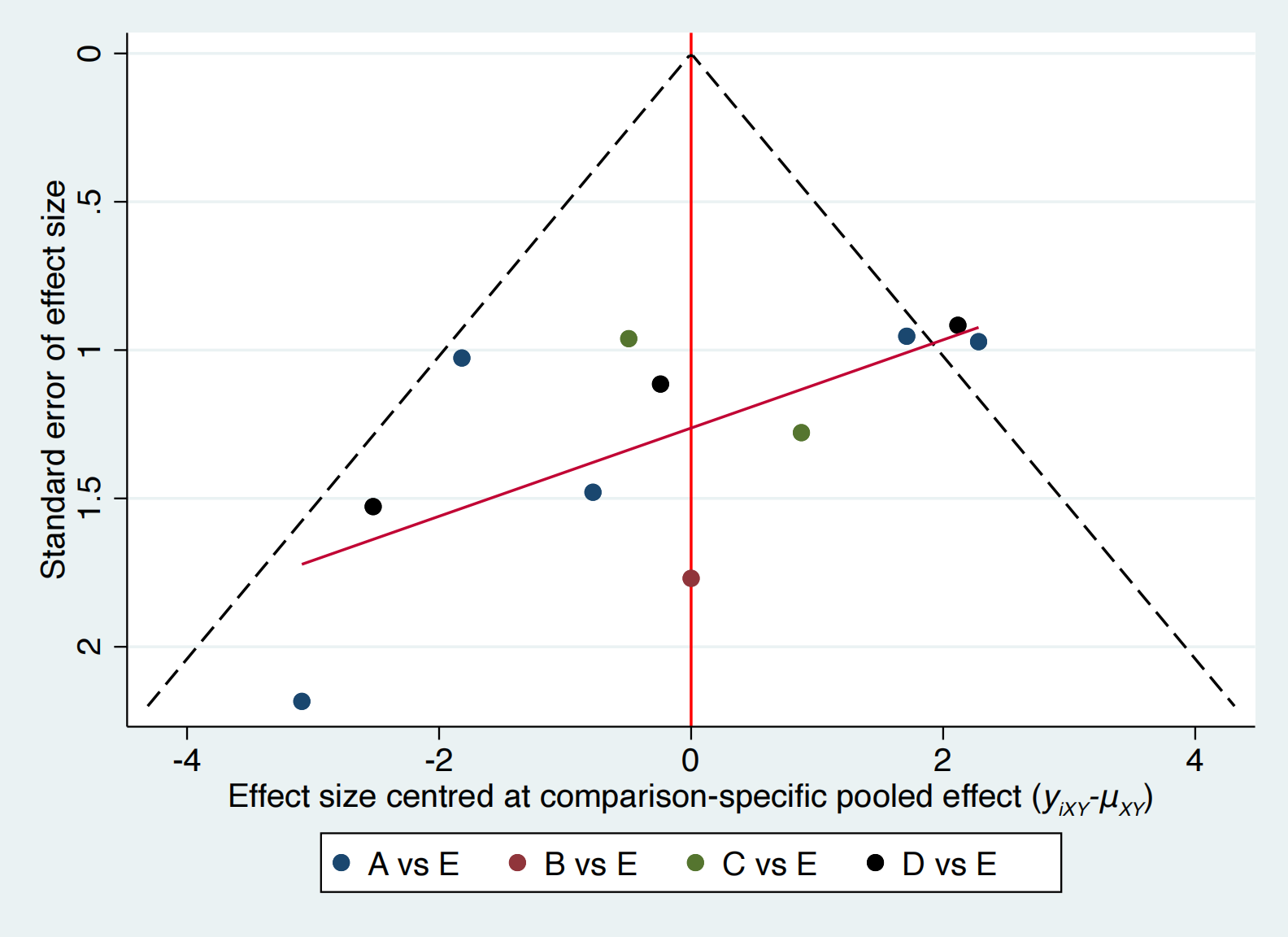
**

**Figure S9.7:** Funnel plot of **PO2**

**
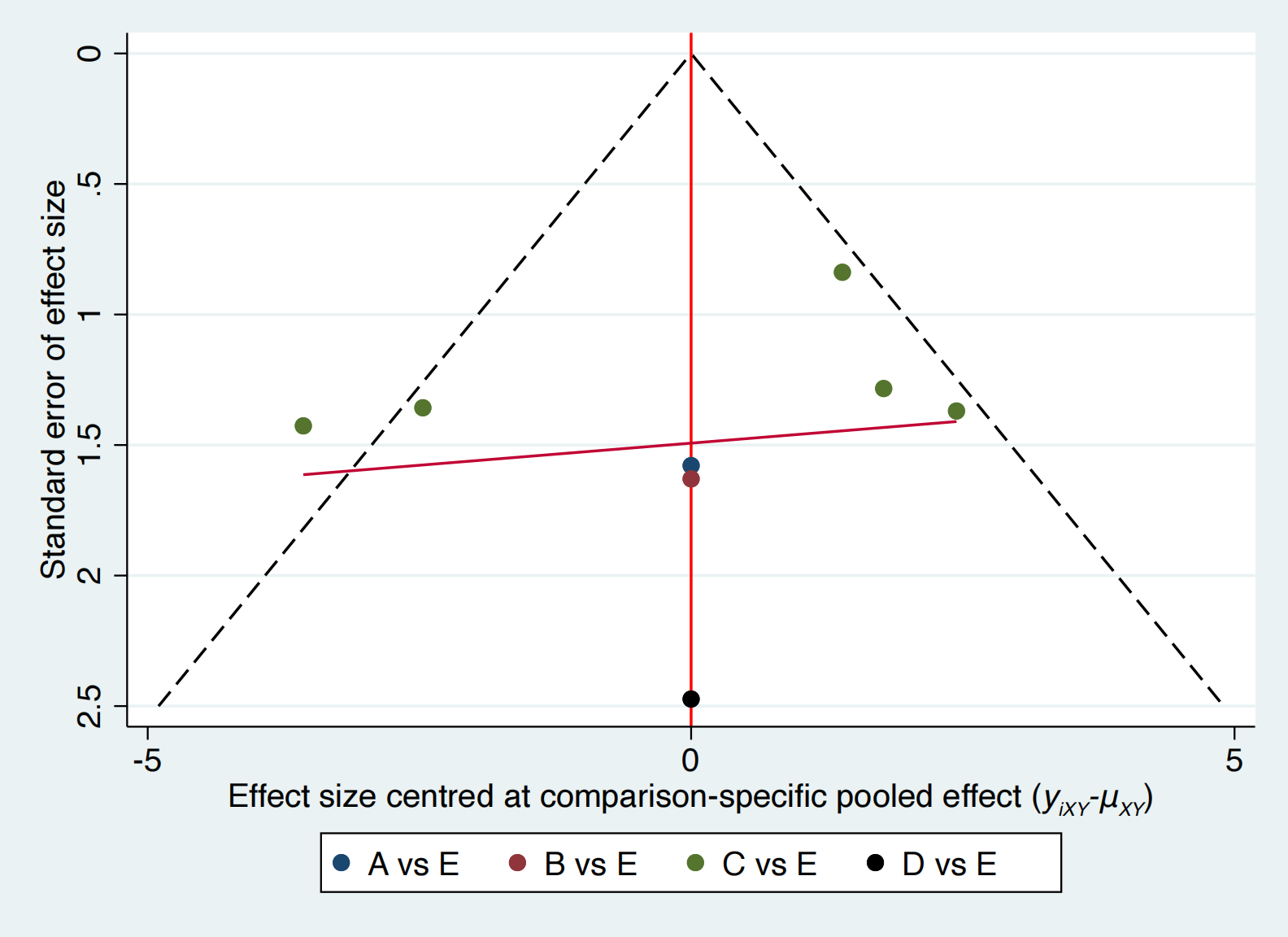
**

**Figure S9.8:** Funnel plot of **PCO2**

**
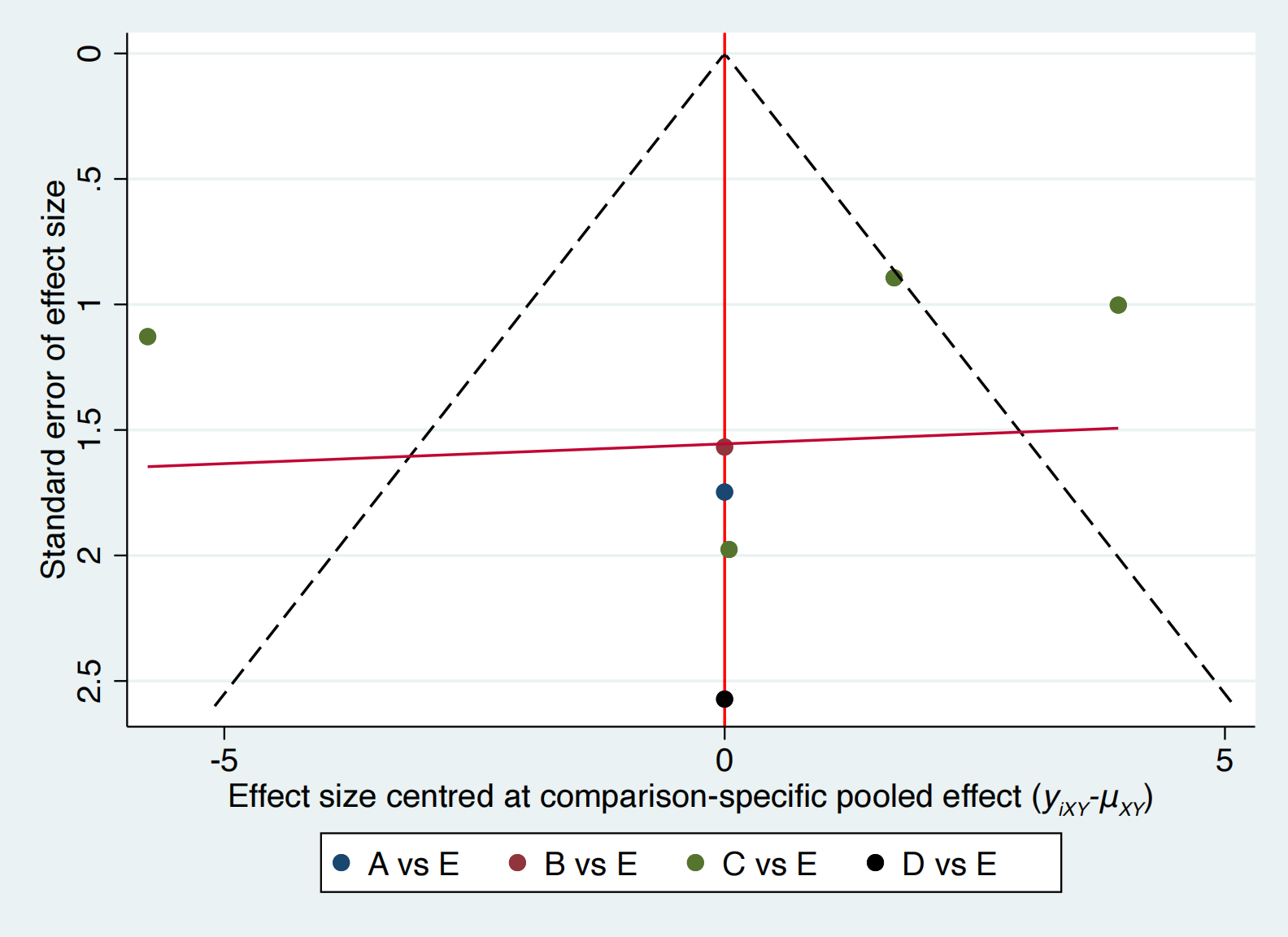
**

**Figure S9.9:** Funnel plot of **TNF-α**

**
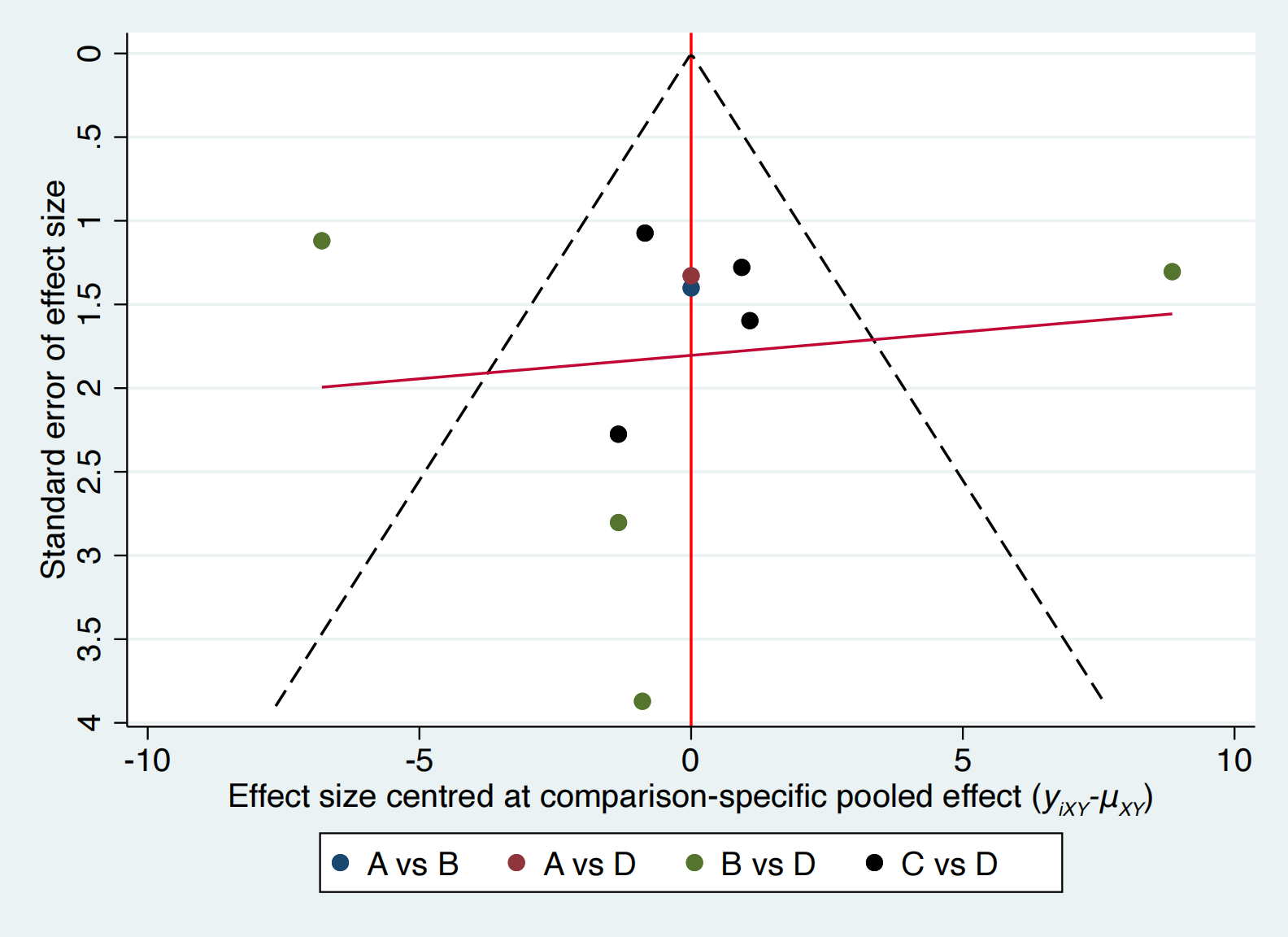
**

**Figure S9.10:** Funnel plot of **hs-CRP**

**
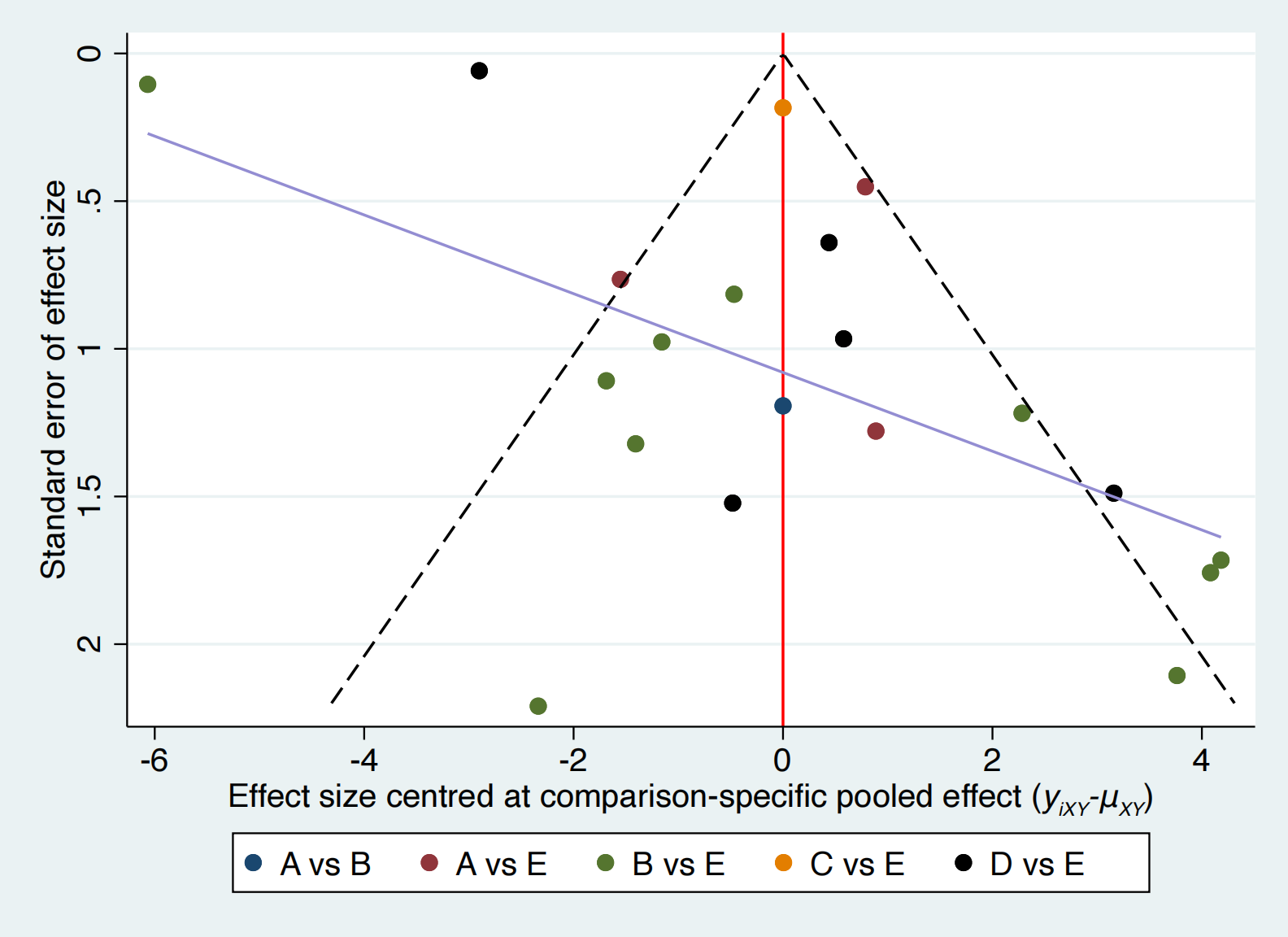
**

**Figure S9.11:** Funnel plot of **IL-6**

**
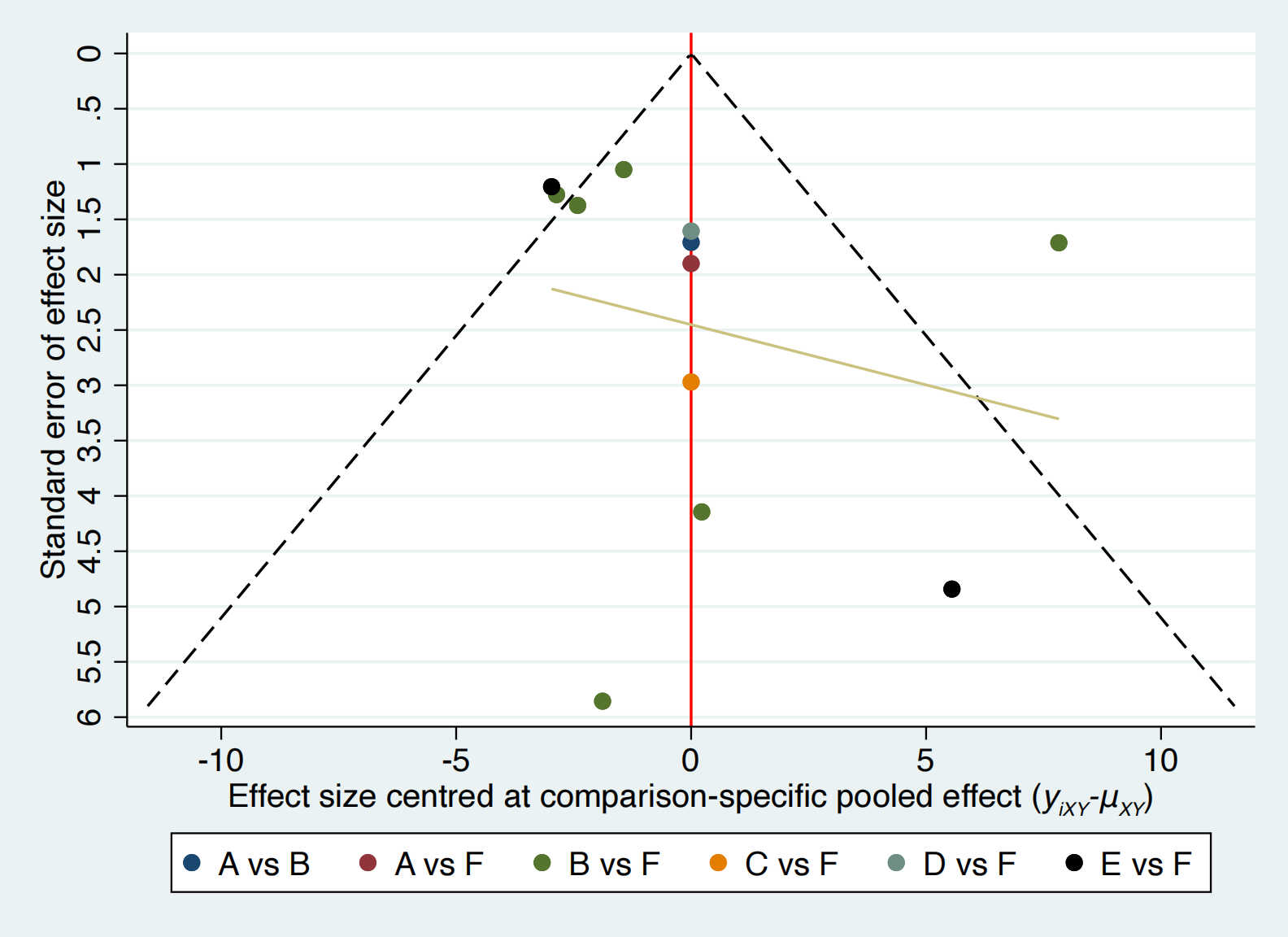
**

**Figure S9.12:** Funnel plot of **NO**

**
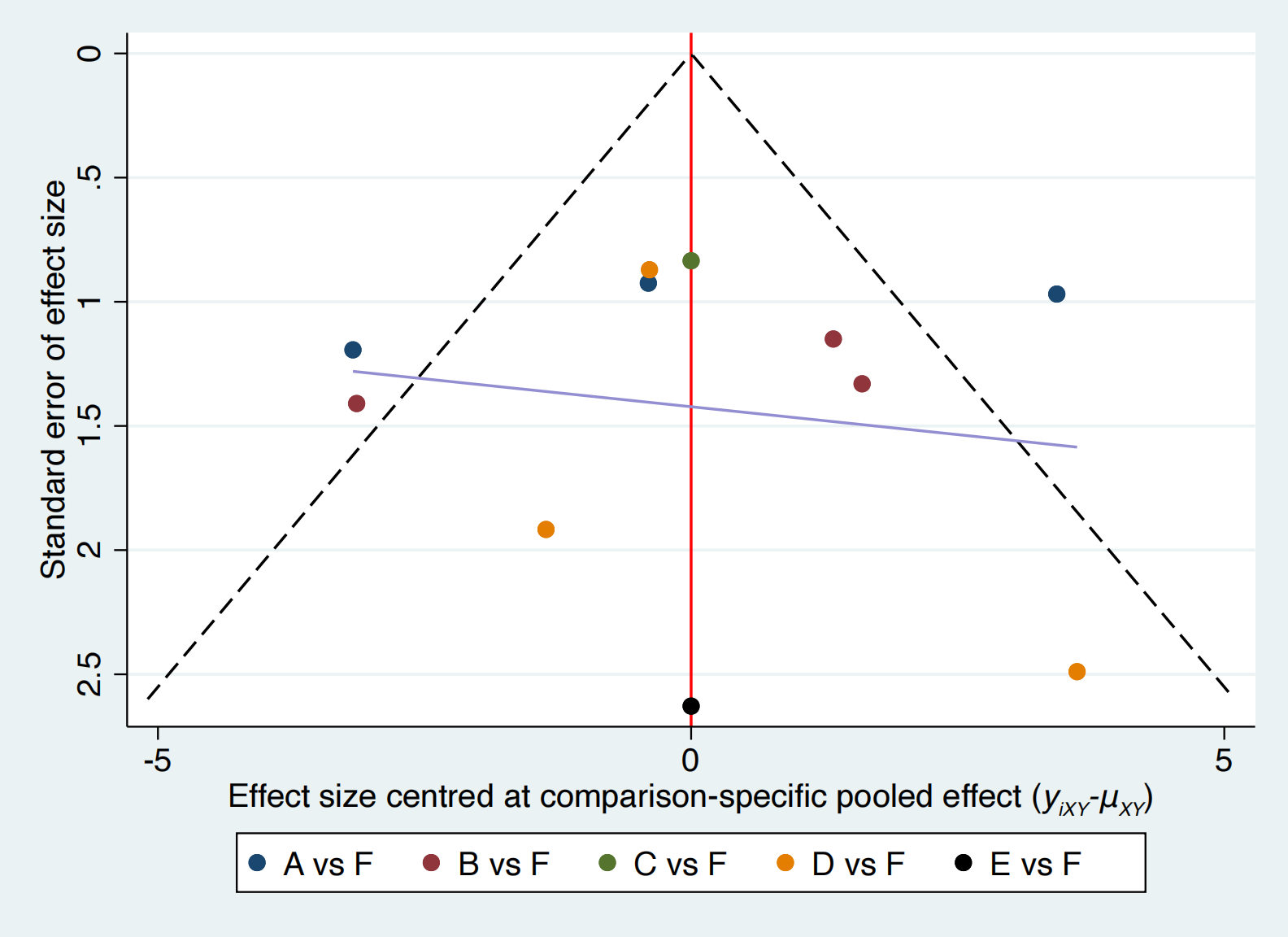
**

**Figure S9.13:** Funnel plot of **ET-1**

**
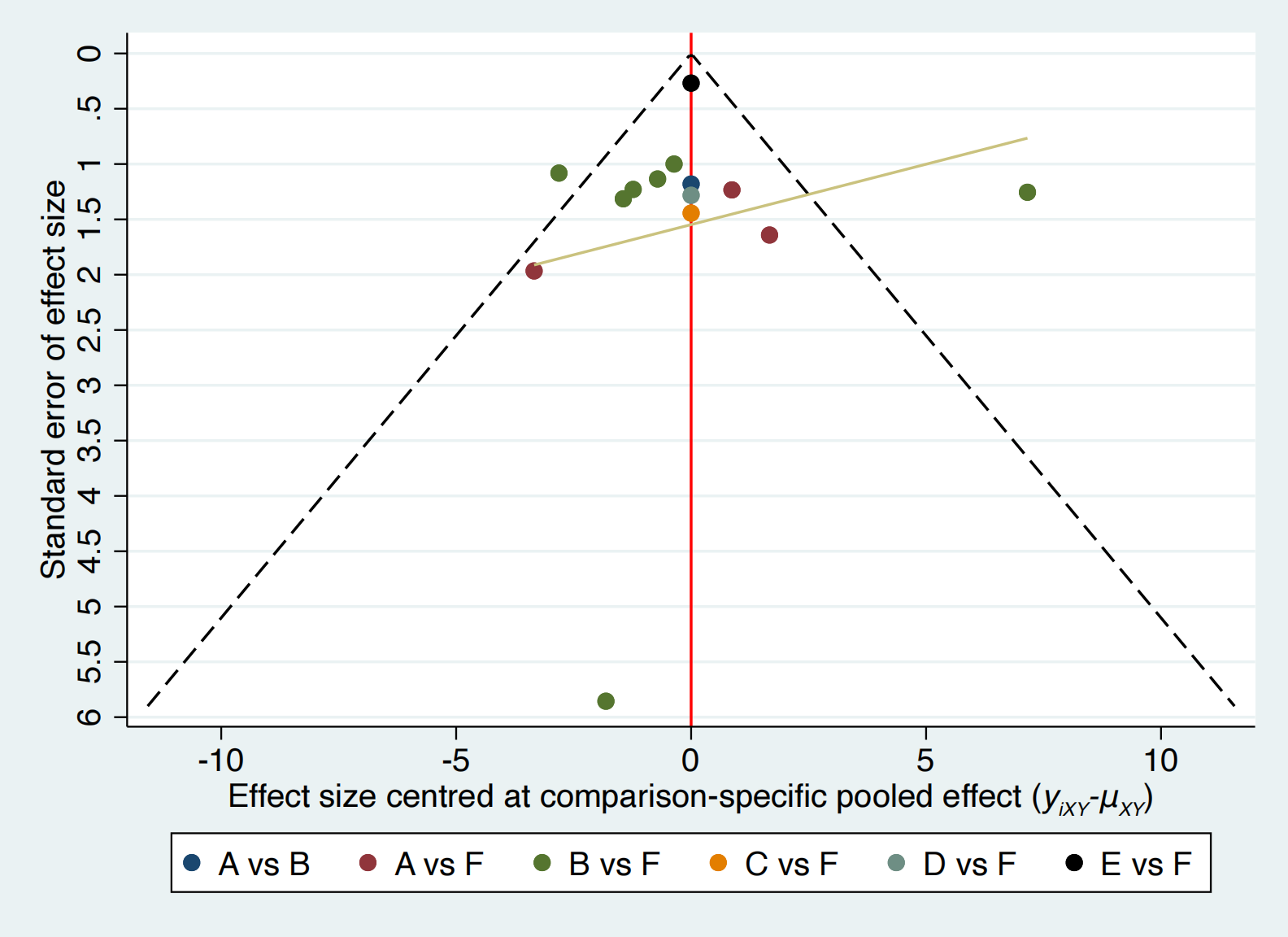
**

**Figure S9.14:** Funnel plot of **Adverse** **events**

**
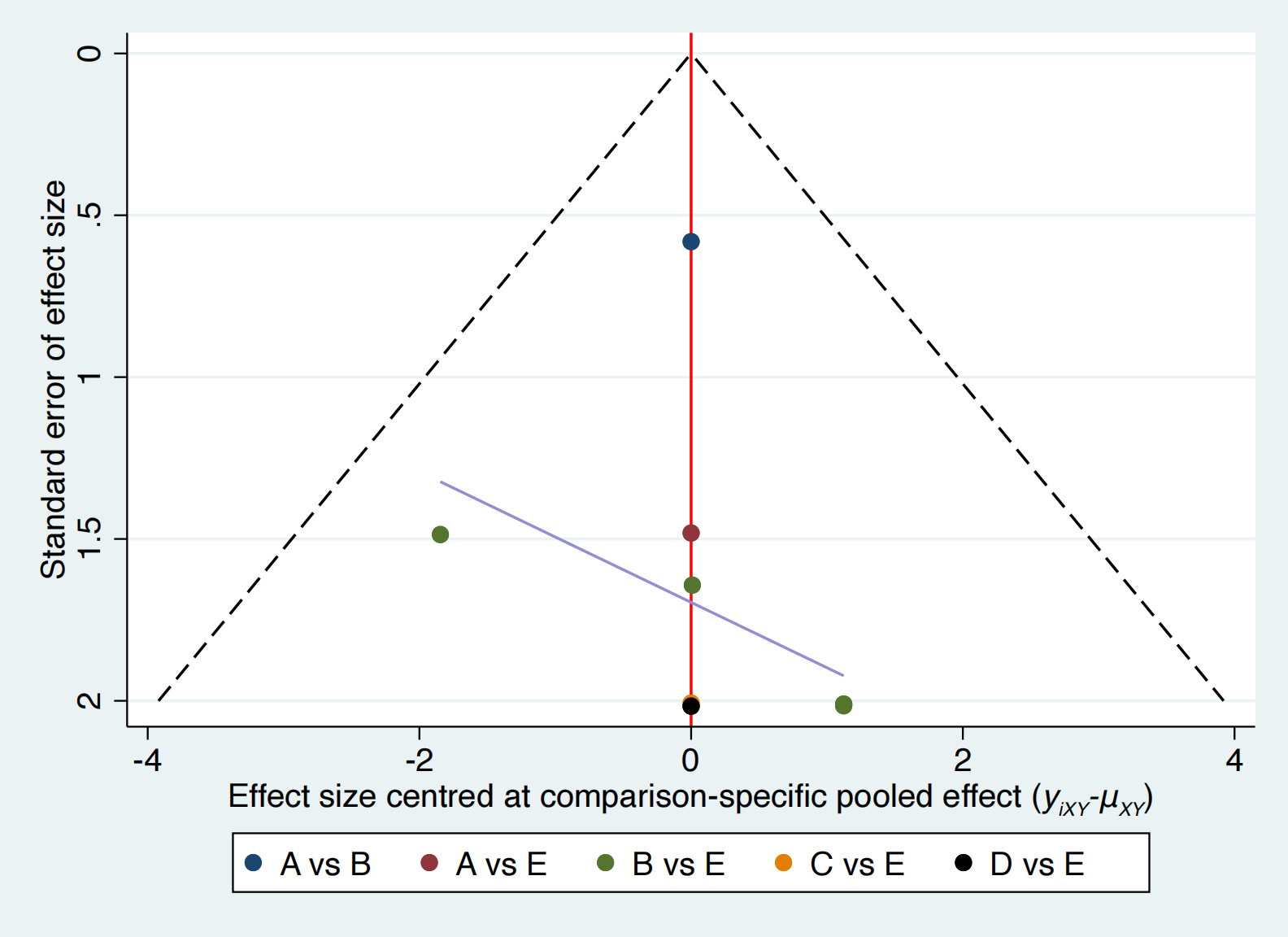
**

**Appendix 10: Network maps and forest plots**

**Figure S10.1**: Network and forest plot of the effect on **6MWD**

**
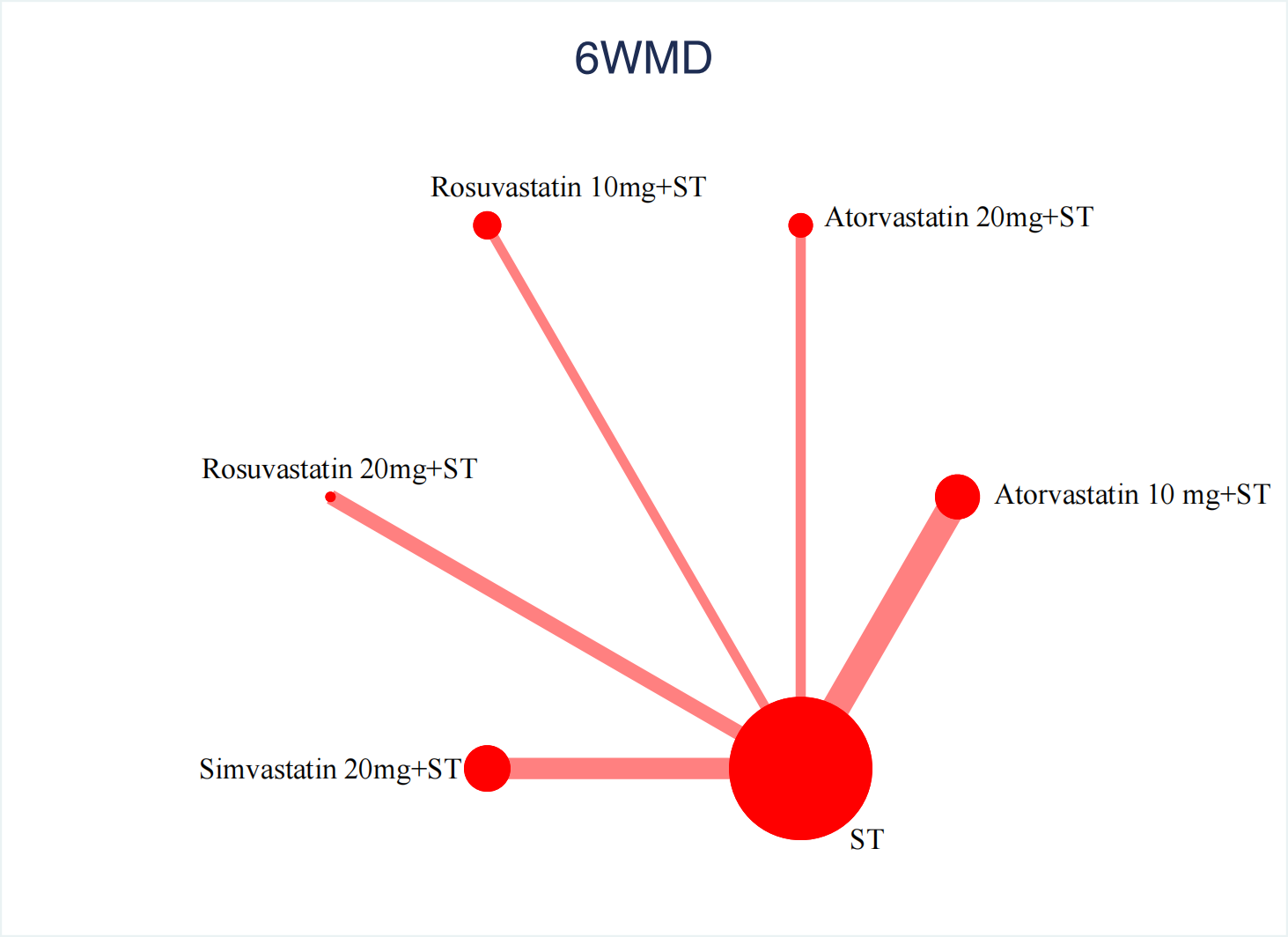
**

**Figure S10.2**: Network and forest plot of the effect on **FVC**

**
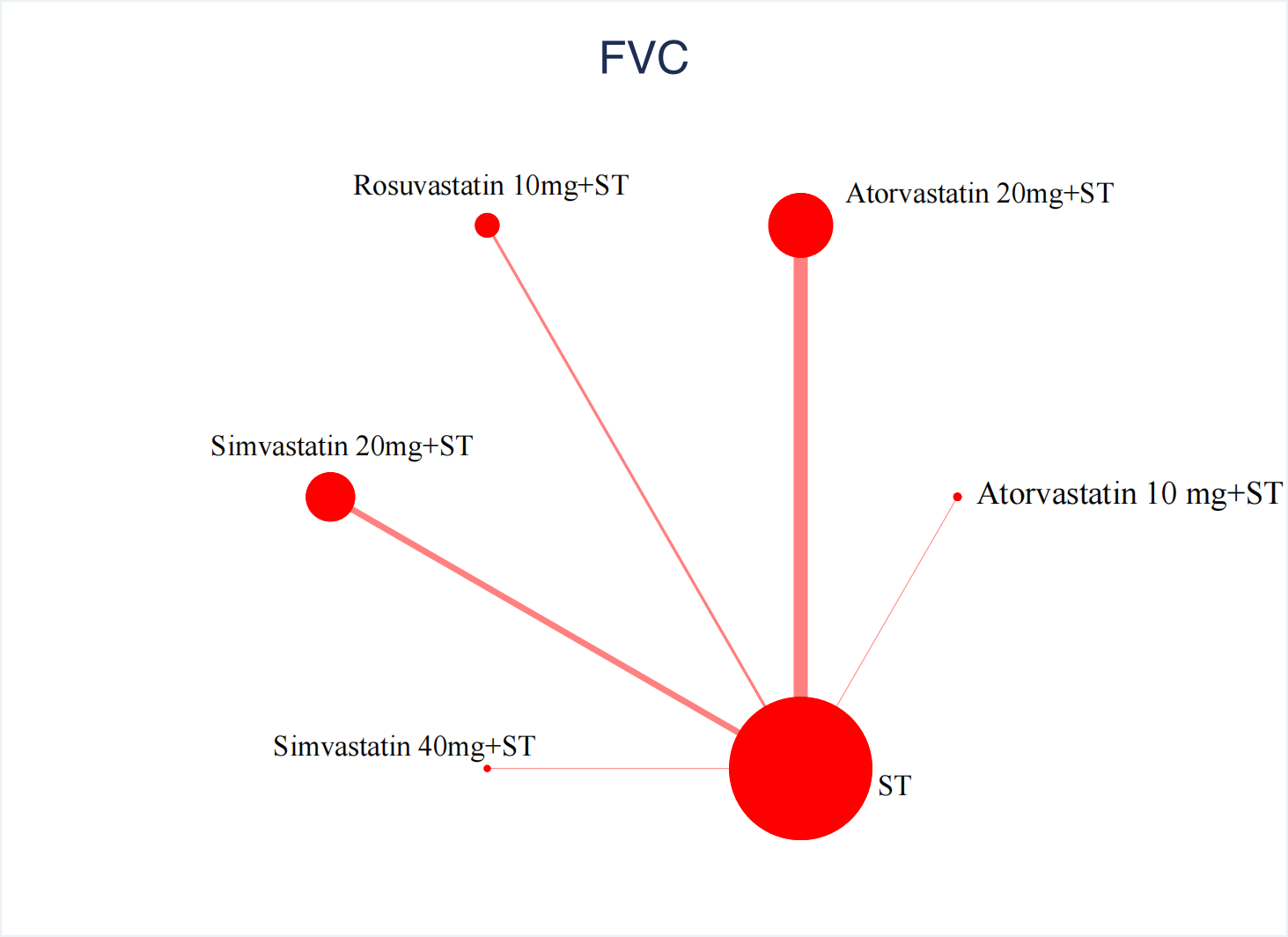
**

**
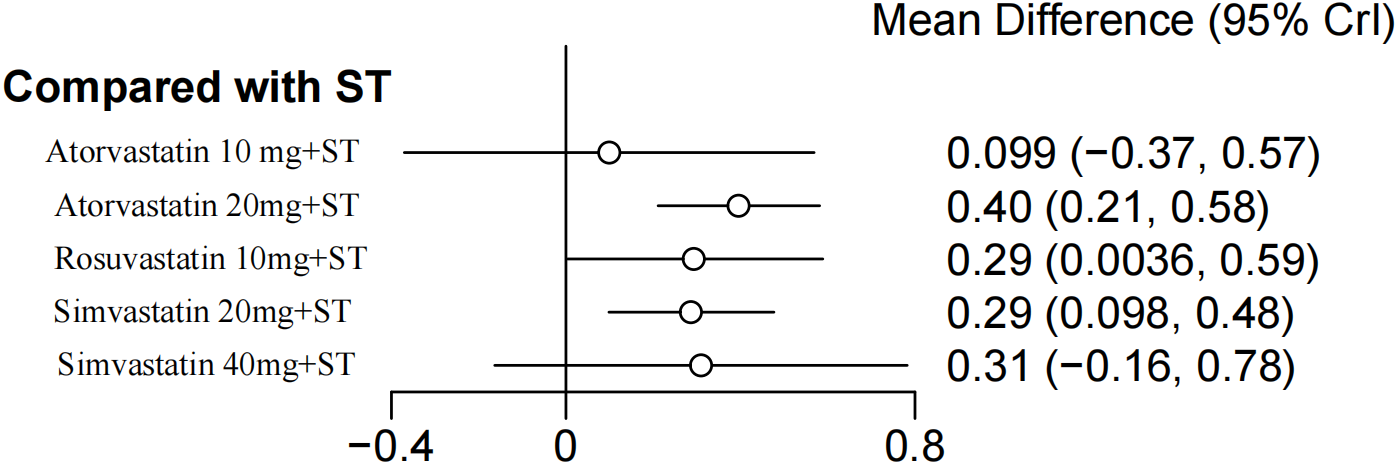
**

**Figure S10.3**: Network and forest plot of the effect on **FEV1**

**
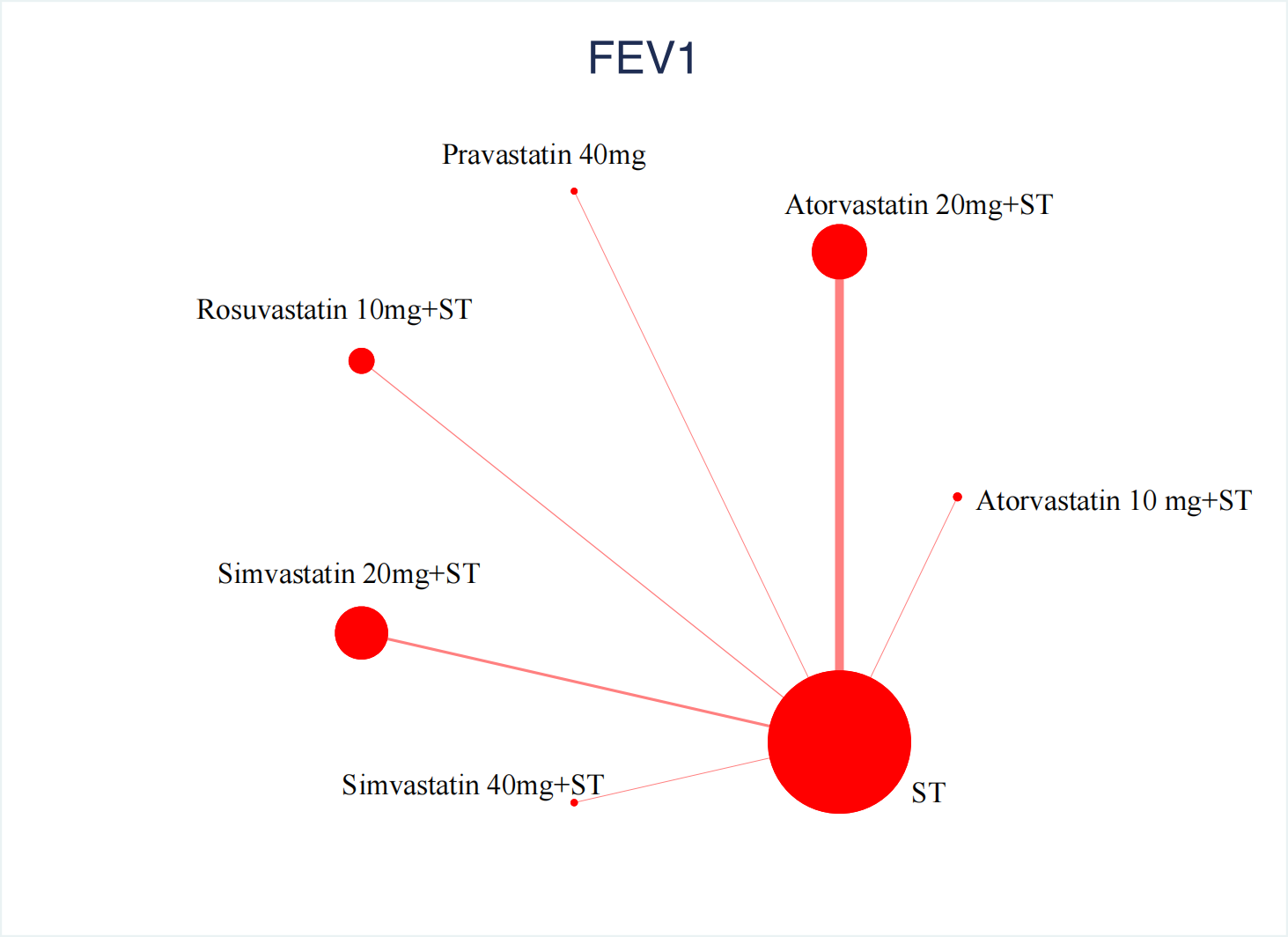
**

**
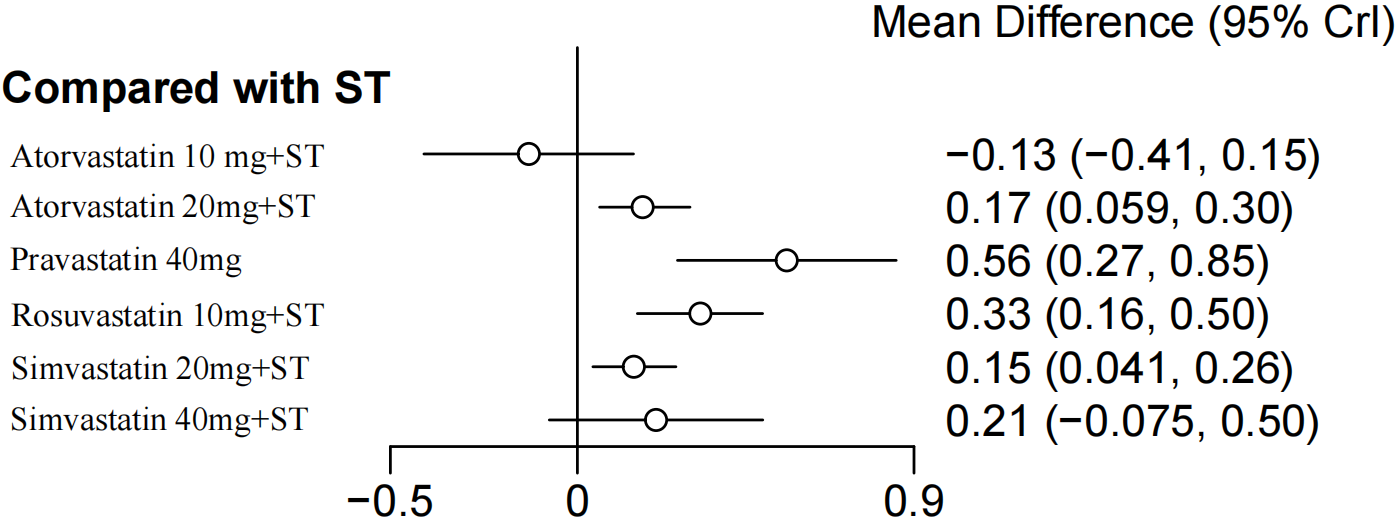
**

**Figure S10.4**: Network and forest plot of the effect on **FEV1/FVC**

**
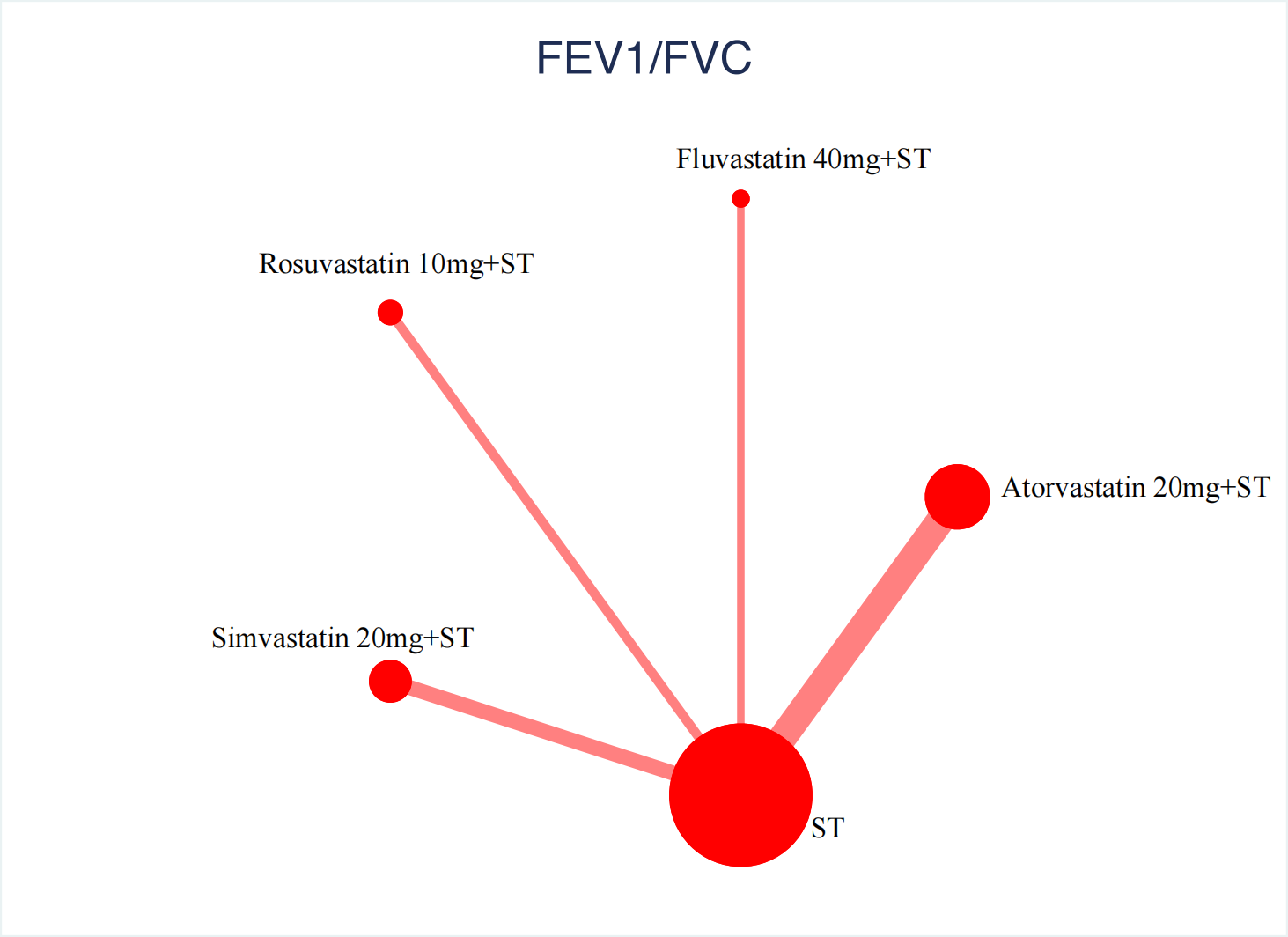
**

**
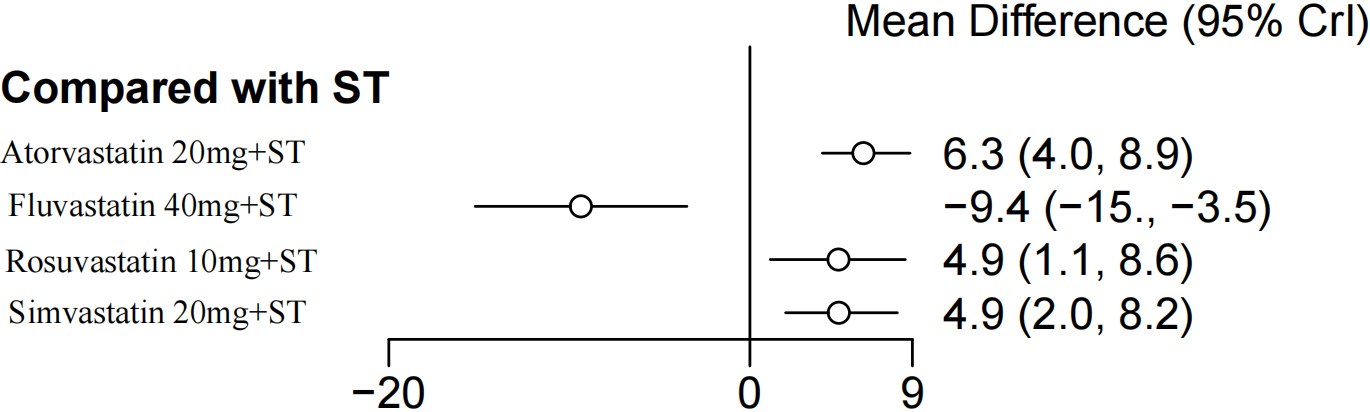
**

**Figure S10.5**: Network and forest plot of the effect on **PO2**

**
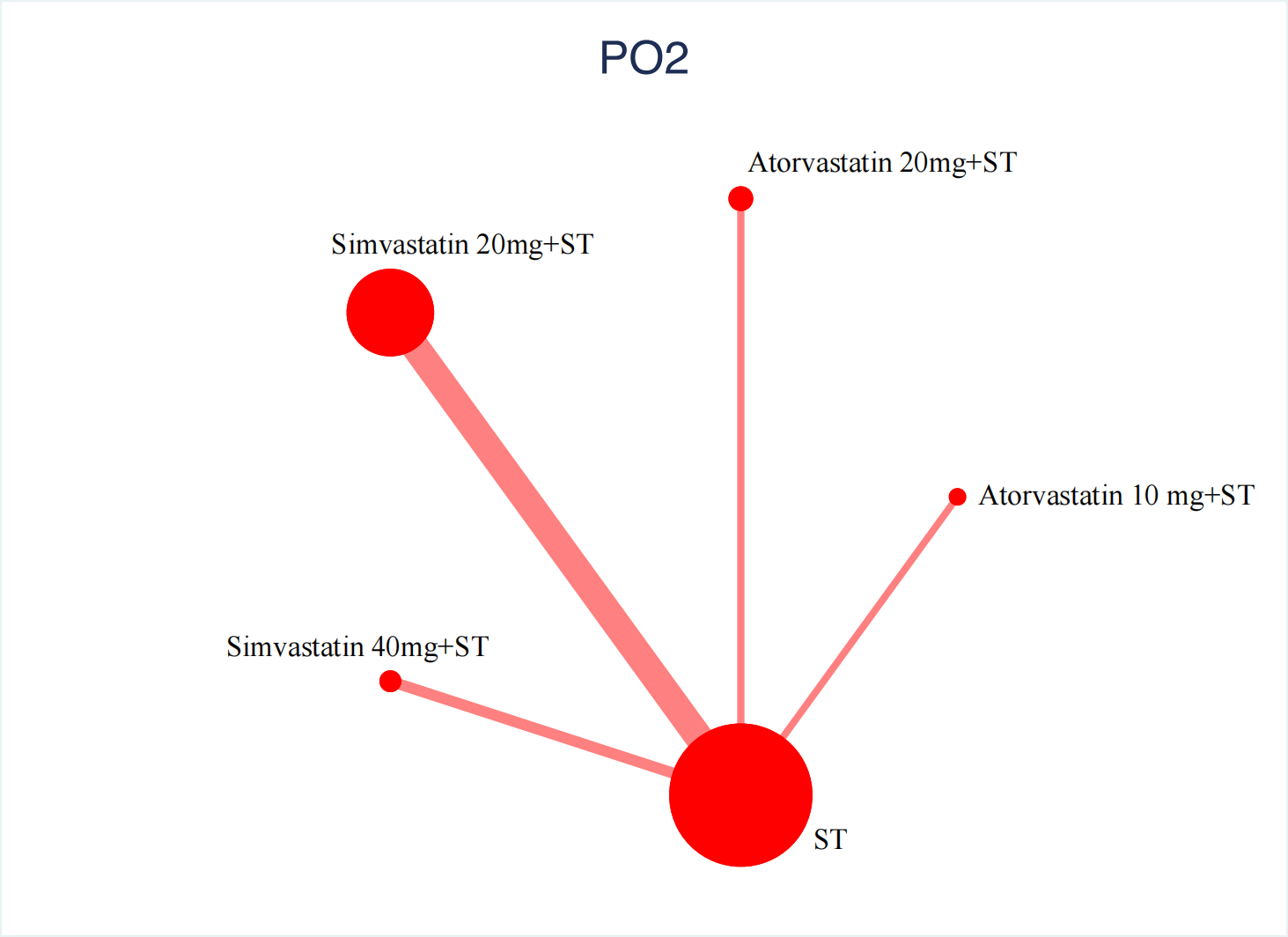
**

**
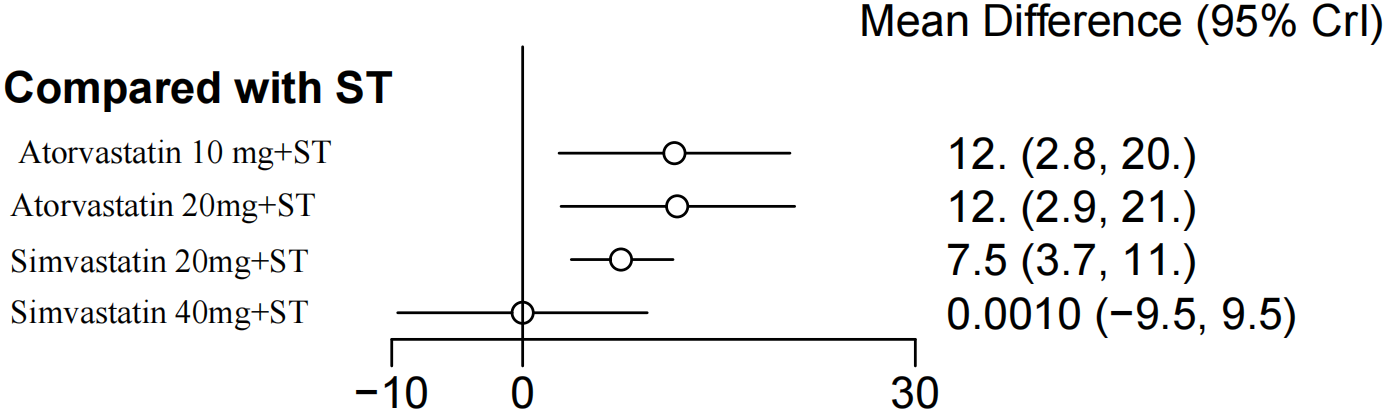
**

**Figure S10.6**: Network and forest plot of the effect on **PCO2**

**
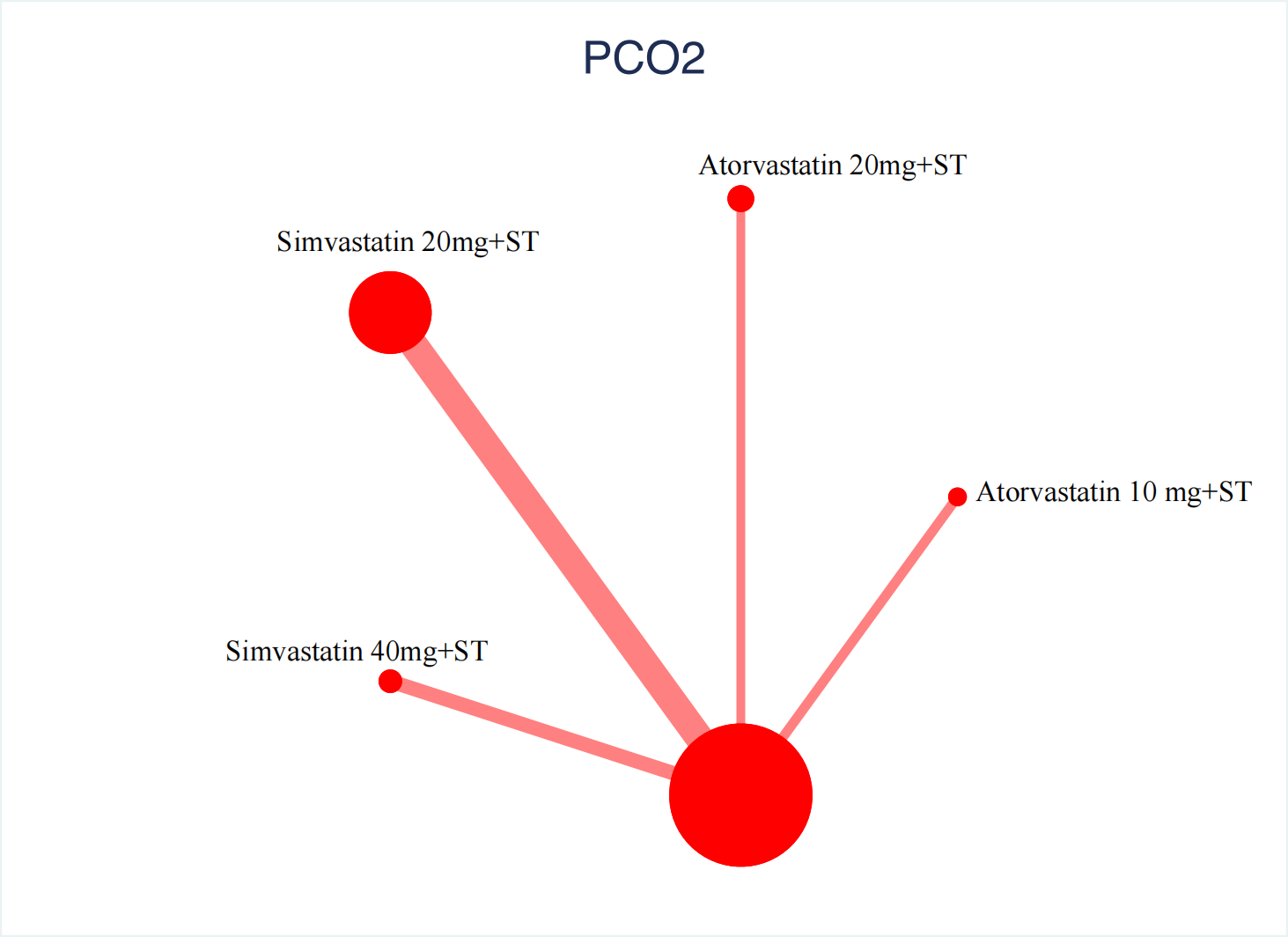
**

**
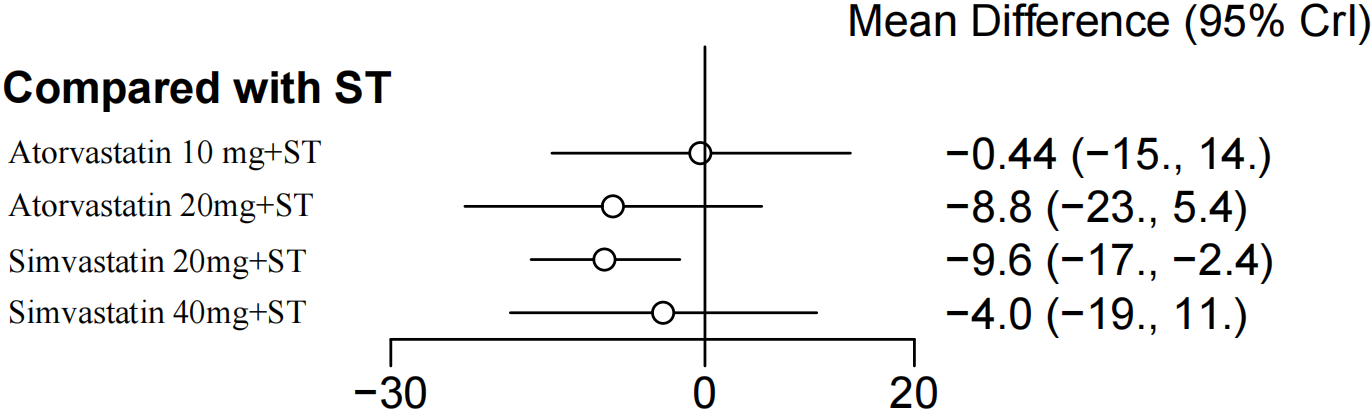
**

**Figure S10.7**: Network and forest plot of the effect on **TNF-α**

**
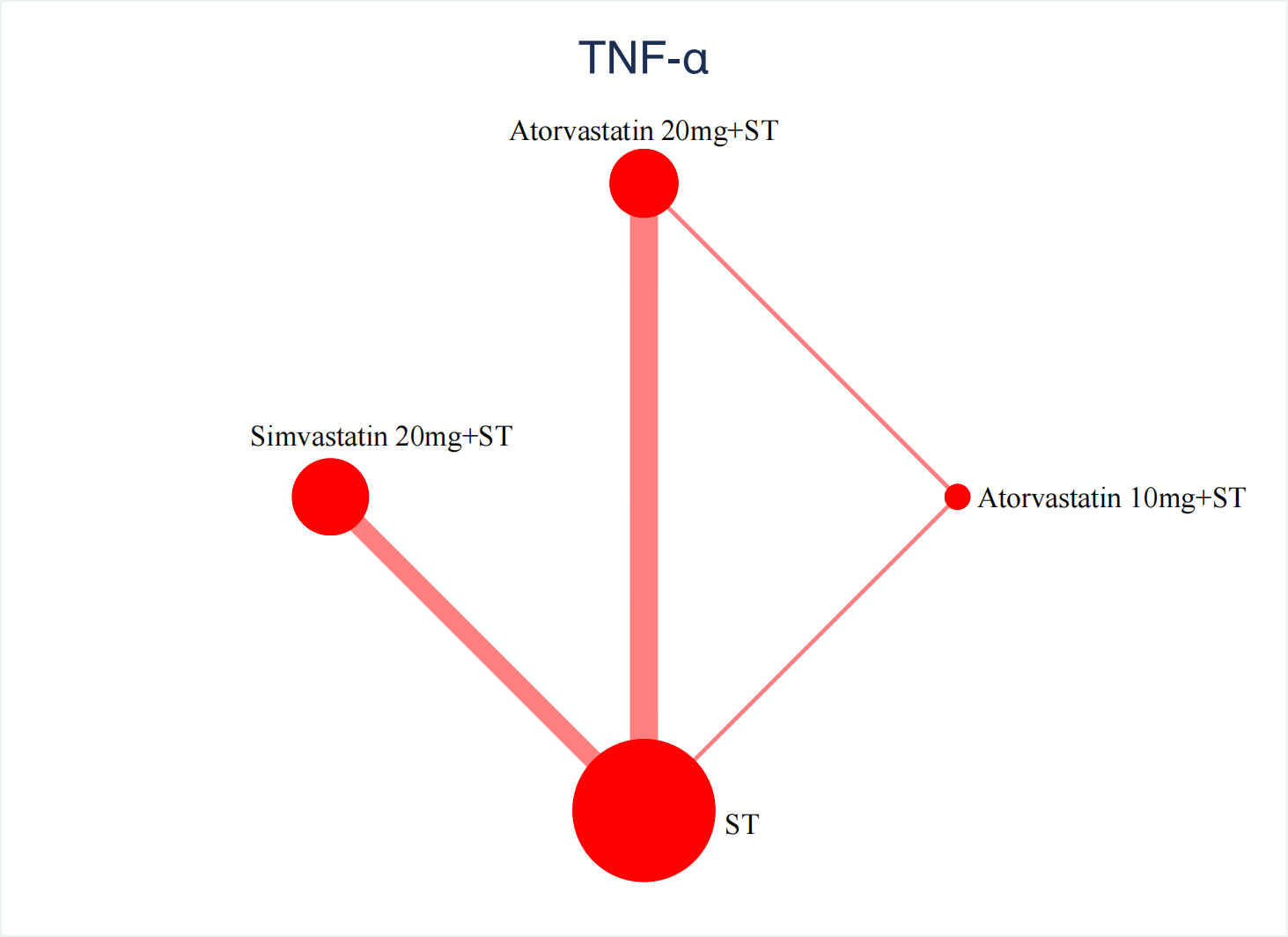
**

**
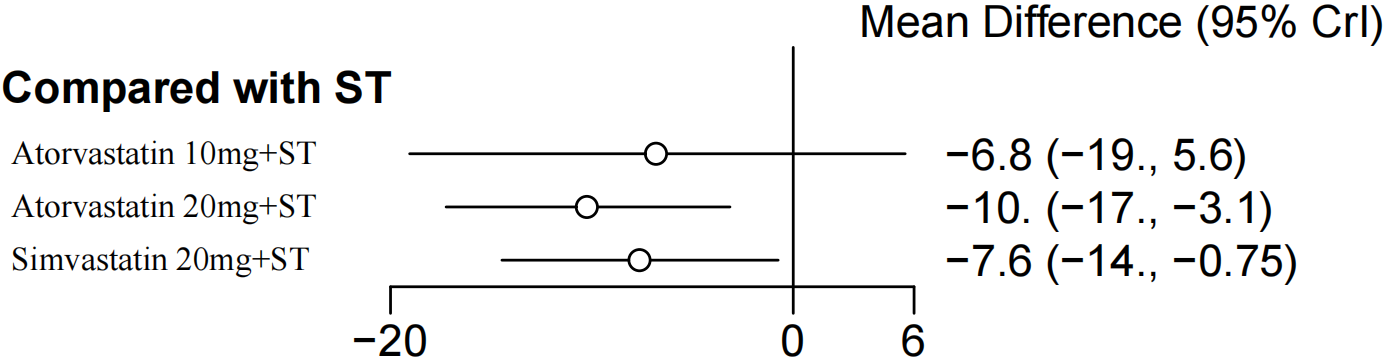
**

**Figure S10.8**: Network and forest plot of the effect on **hs-CRP**

**
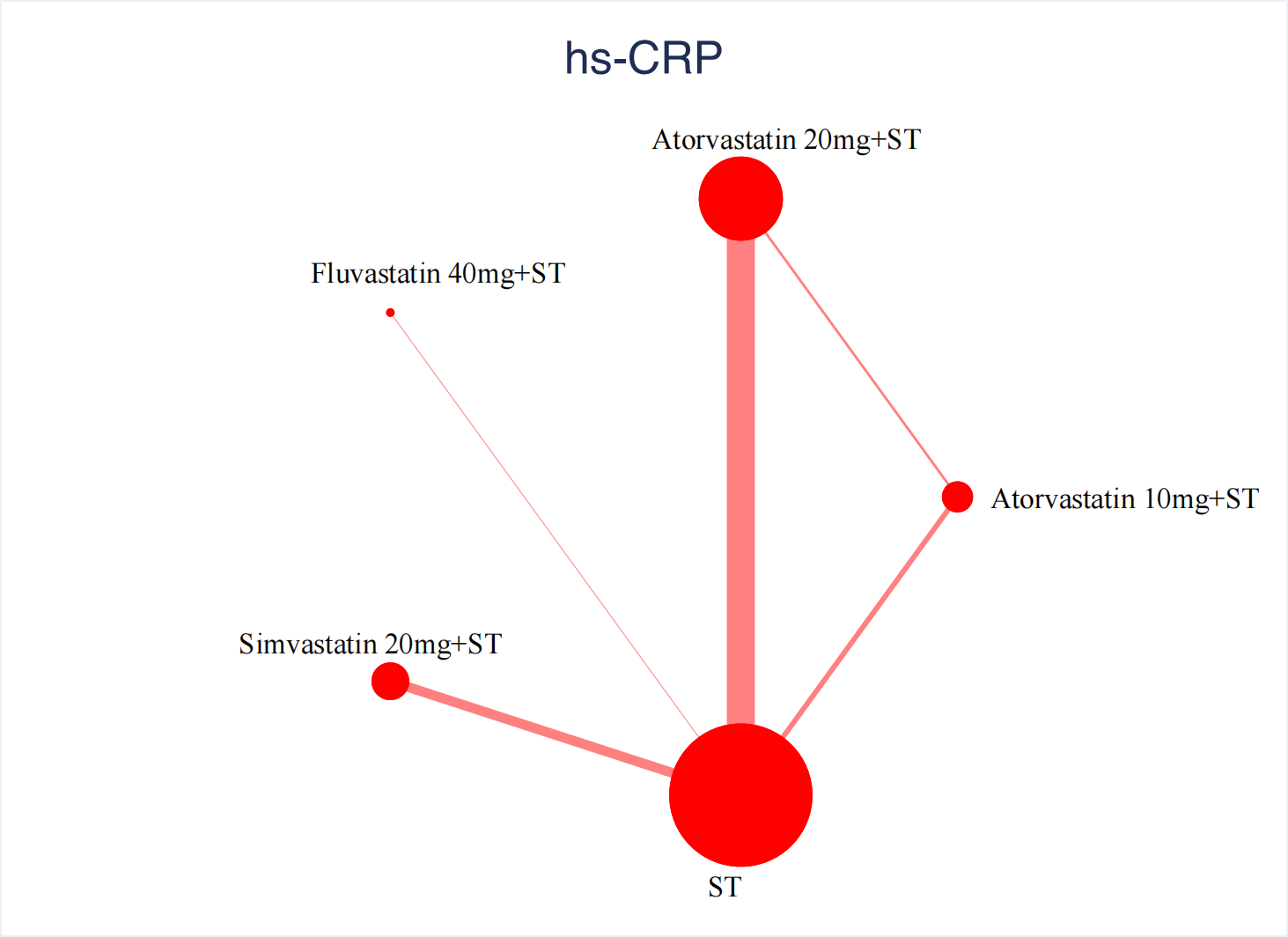
**

**
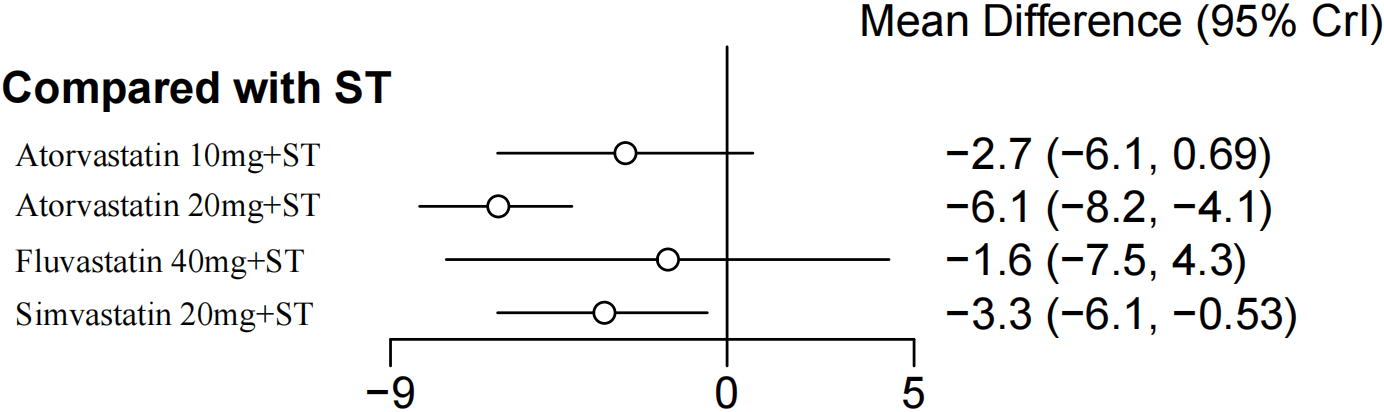
**

**Figure S10.9**:Network and forest plot of the effect on **IL-6**

**
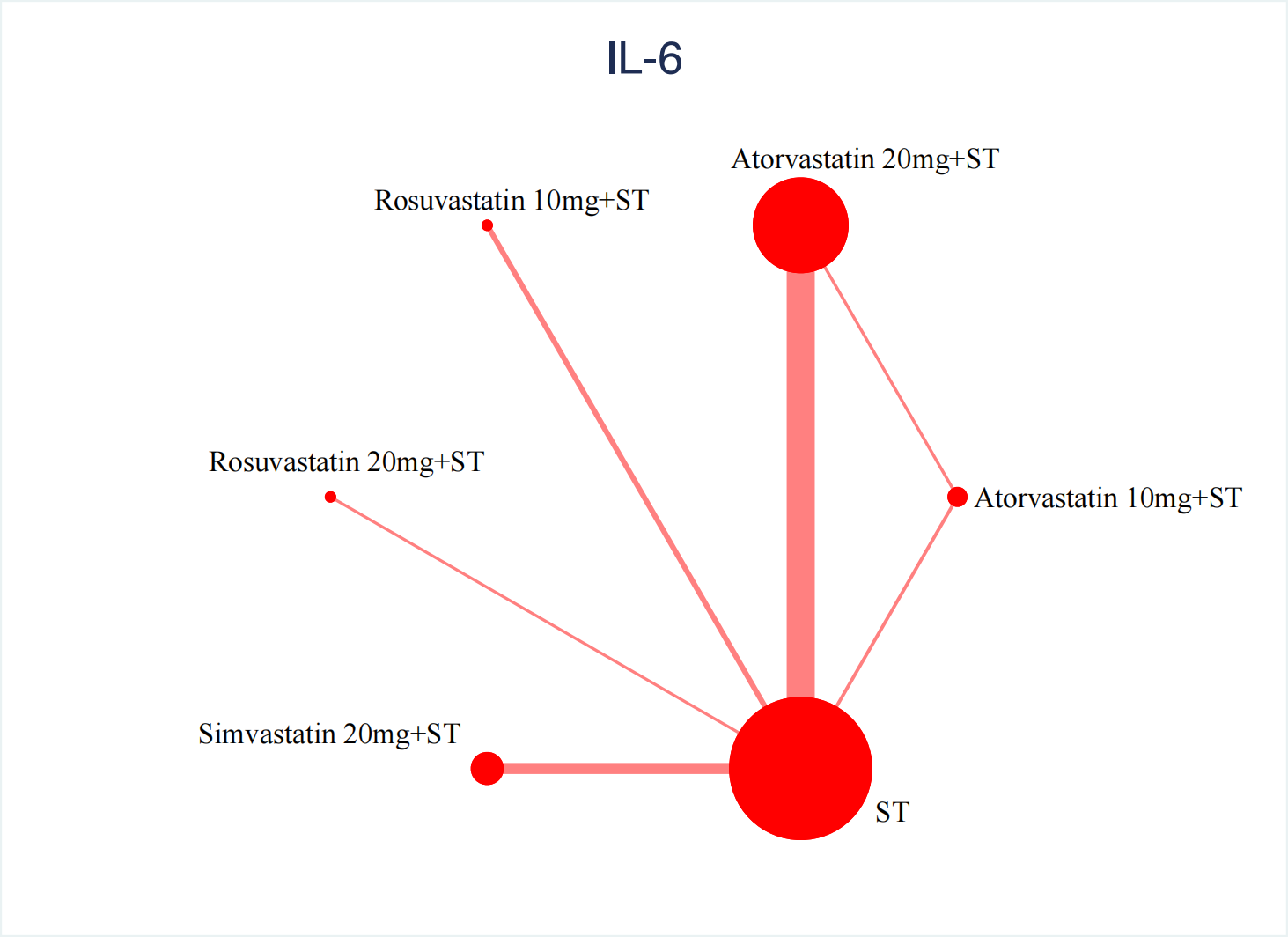
**

**
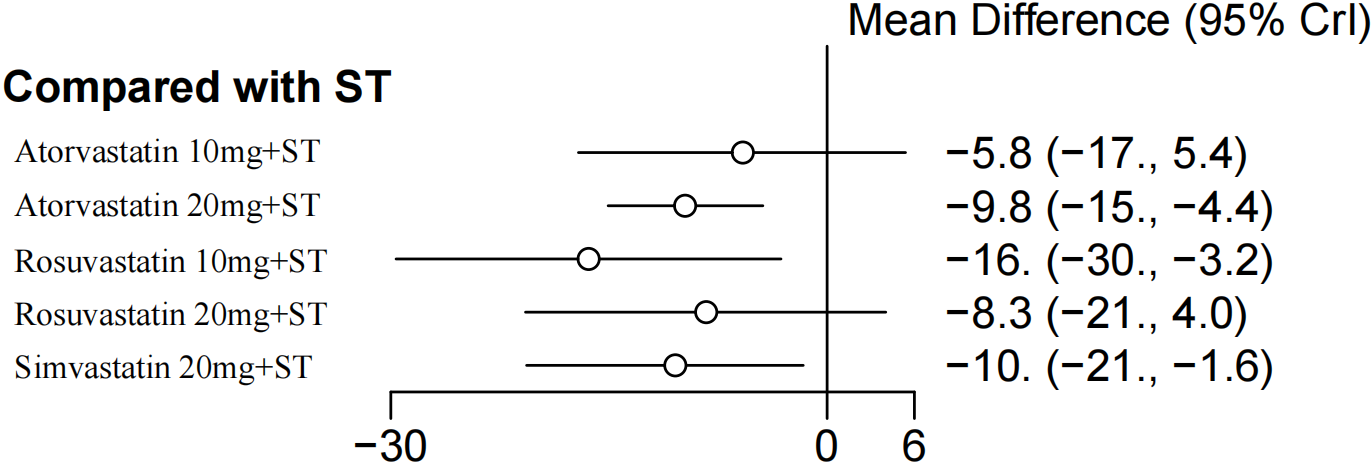
**

**Figure S10.10**: Network and forest plot of the effect on **NO**

**
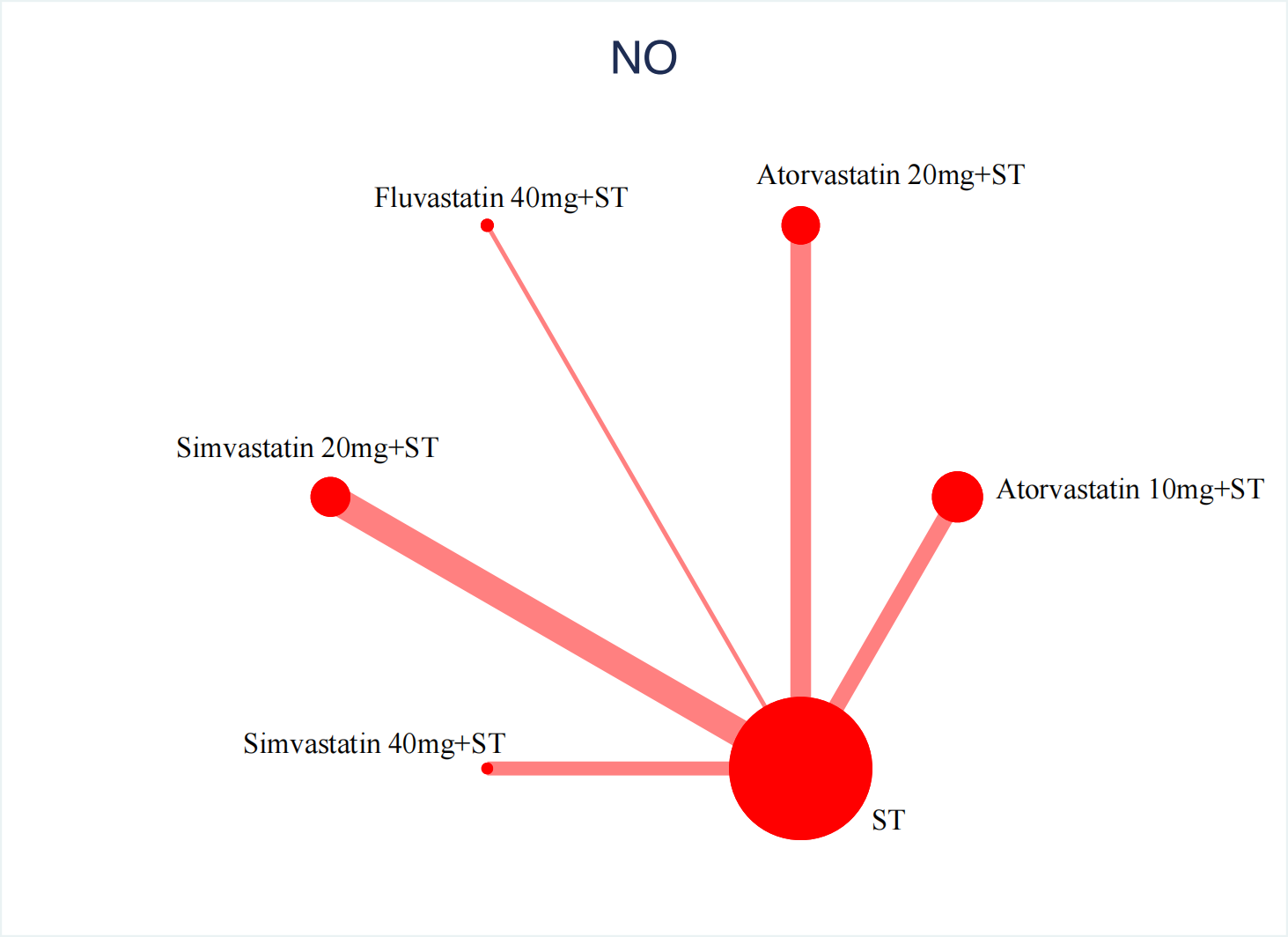
**

**
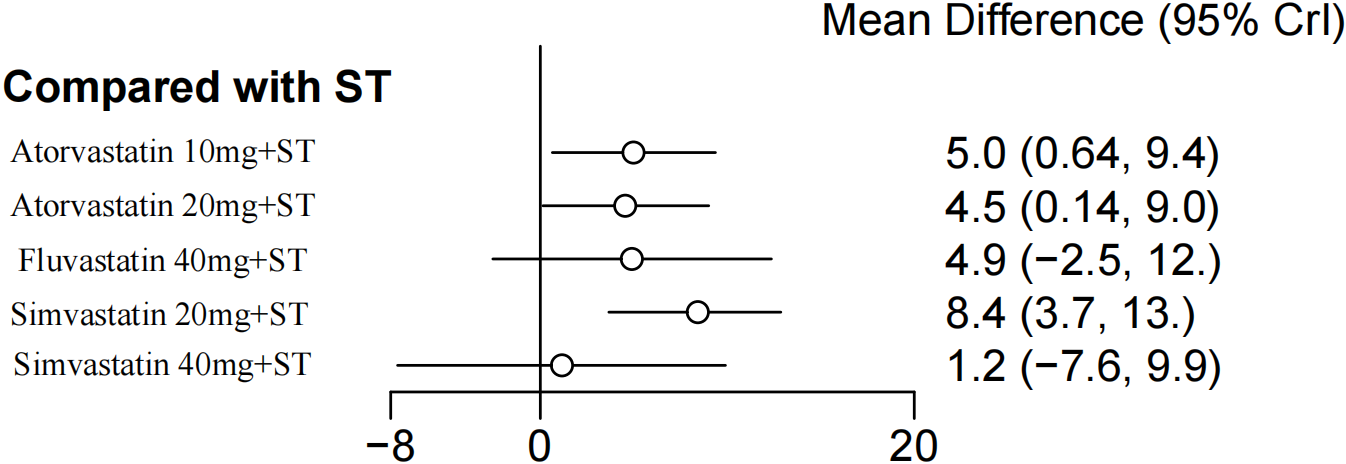
**

**Figure S10.11**: Network and forest plot of the effect on **ET-1**

**
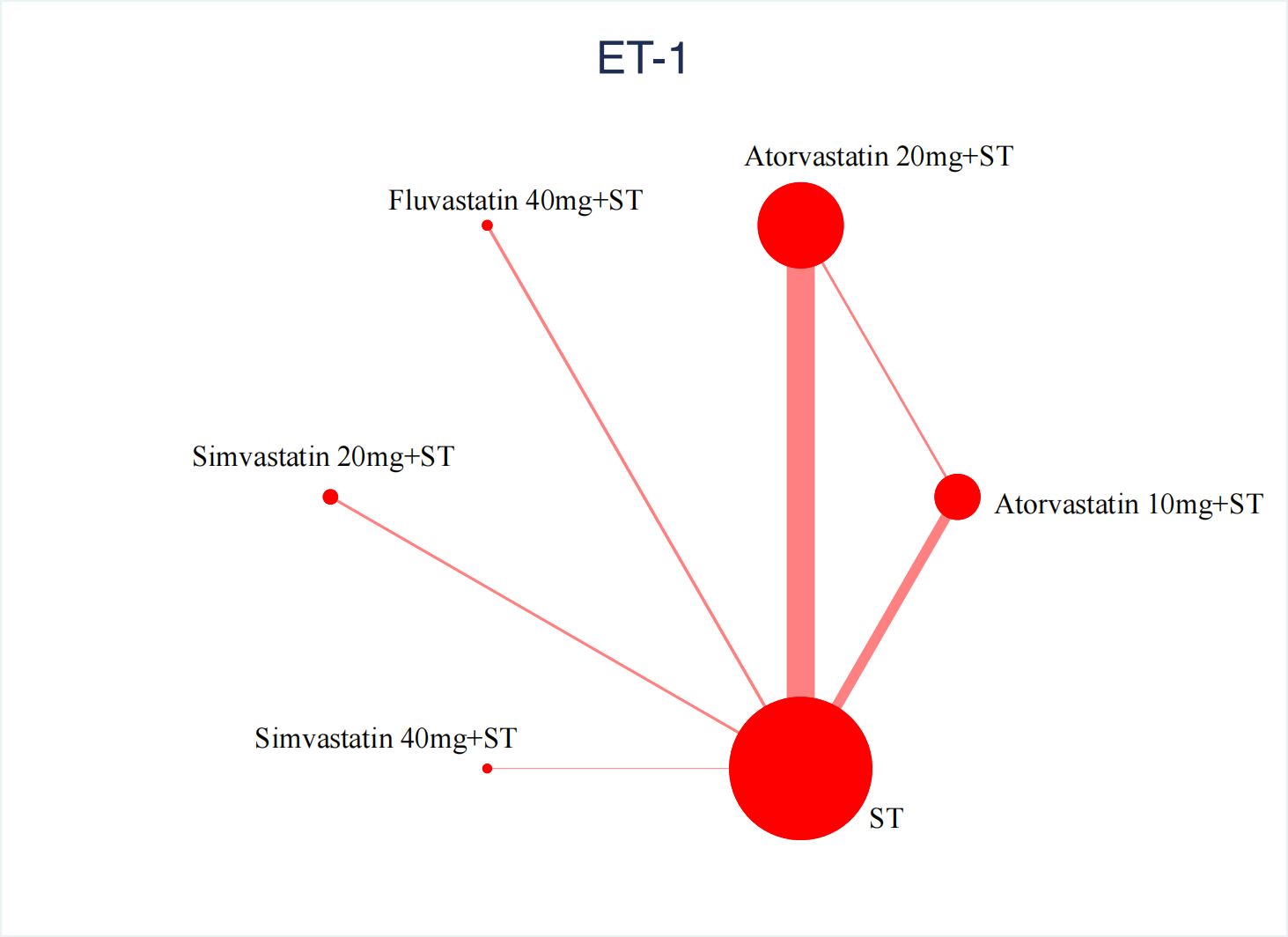
**

**
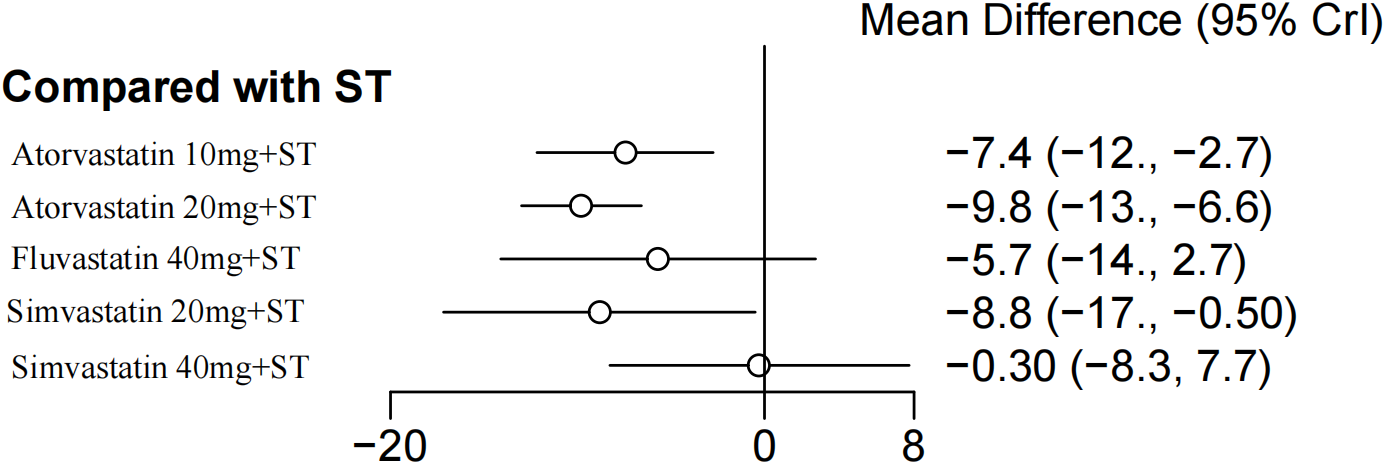
**

**Appendix 11: SUCRA and cumulative probability plots**

**Figure S11.1**: Cumulative ranking curve plots of Stains for **sPAP** in range network. Higher surface under the curve reflects higher probability of association with **sPAP**.


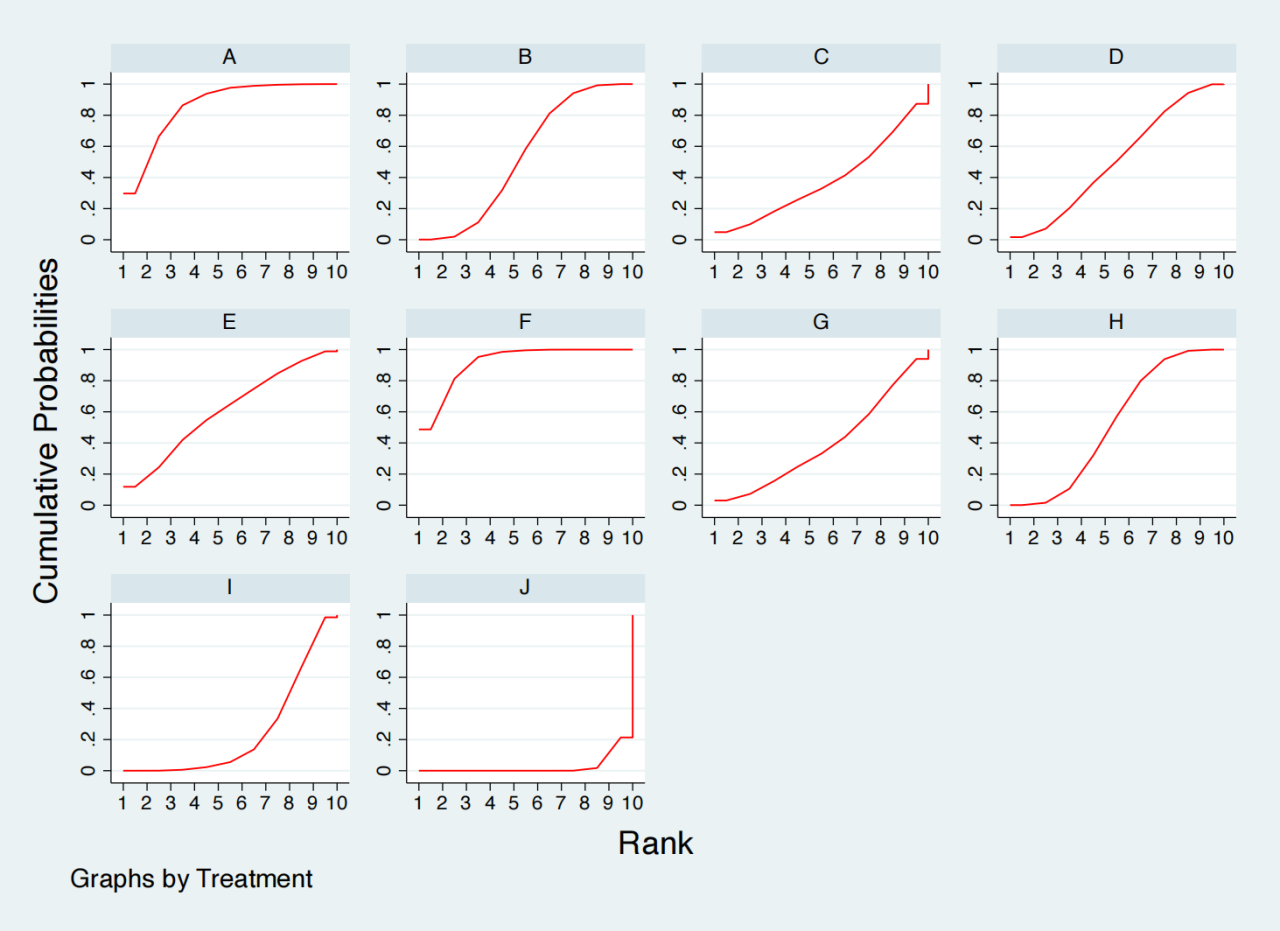


**Abbreviations:**SUCRA, surface under the cumulative ranking curve; A, Atorvastatin 10mg+ST; B, Atorvastatin 20mg+ST; C, Atorvastatin 40mg+ST; D, Fluvastatin 40mg+ST; E, Pravastatin 40mg; F, Rosuvastatin 10mg+ST; G, Rosuvastatin 20mg+ST; H, Simvastatin 20mg+ST; I, Simvastatin 40mg+ST; J, ST.

**Table S11.1**: SUCRA of the effects of various Stains on **sPAP**

| **Treatment** | **SUCRA** | **PrBest** | **MeanRank** |
| --- | --- | --- | --- |
| Atorvastatin 10 mg+ST | 85.8 | 29.7 | 2.3 |
| Atorvastatin 20mg+ST | 53.1 | 0.1 | 5.2 |
| Atorvastatin 40mg+ST | 38.1 | 4.9 | 6.6 |
| Fluvastatin 40mg+ST | 51 | 1.7 | 5.4 |
| Pravastatin 40mg | 61 | 11.8 | 4.5 |
| Rosuvastatin 10mg+ST | 91.5 | 48.7 | 1.8 |
| Rosuvastatin 20mg+ST | 39.7 | 3 | 6.4 |
| Simvastatin 20mg+ST | 52.7 | 0.1 | 5.3 |
| Simvastatin 40mg+ST | 24.5 | 0 | 7.8 |
| ST | 2.6 | 0 | 9.8 |

**Figure S11.2**: Cumulative ranking curve plots of Stains for **mPAP** in range network. Higher surface under the curve reflects higher probability of association with **mPAP**.


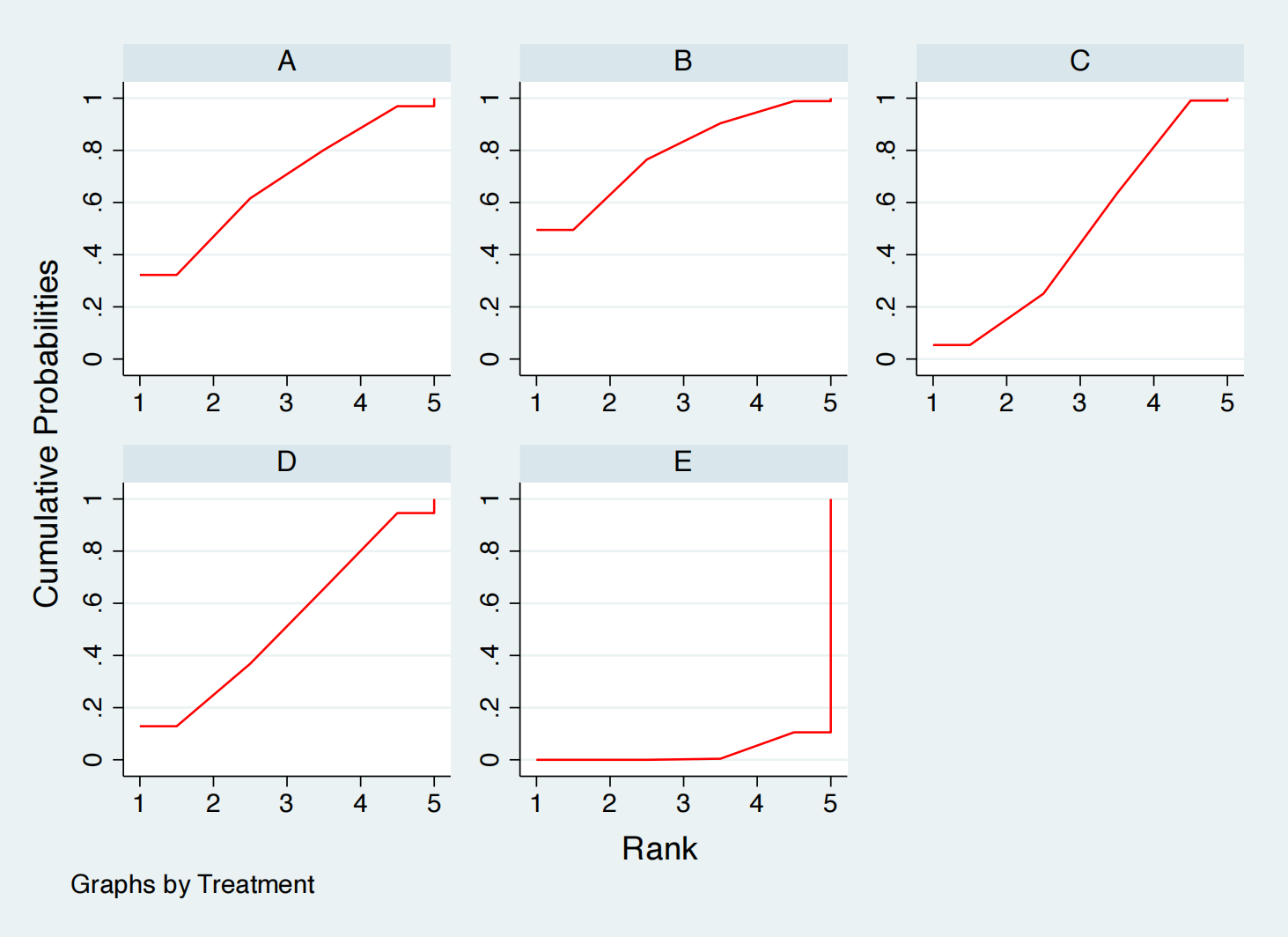


**Abbreviations:**SUCRA, surface under the cumulative ranking curve; A, Atorvastatin 20mg+ST; B, Rosuvastatin 20mg+ST; C, Simvastatin 20mg+ST; D, Simvastatin 40mg+ST; E, ST.

**Table S11.2**: SUCRA of the effects of various Stains on **mPAP**

| **Treatment** | **SUCRA** | **PrBest** | **MeanRank** |
| --- | --- | --- | --- |
| Atorvastatin 20mg+ST | 67.7 | 32.2 | 2.3 |
| Rosuvastatin 20mg+ST | 78.8 | 49.5 | 1.8 |
| Simvastatin 20mg+ST | 48.2 | 5.4 | 3.1 |
| Simvastatin 40mg+ST | 52.5 | 12.9 | 2.9 |
| ST | 2.7 | 0 | 4.9 |

**Figure S11.3**: Cumulative ranking curve plots of Stains for **6MWD** in range network. Higher surface under the curve reflects higher probability of association with **6MWD**.


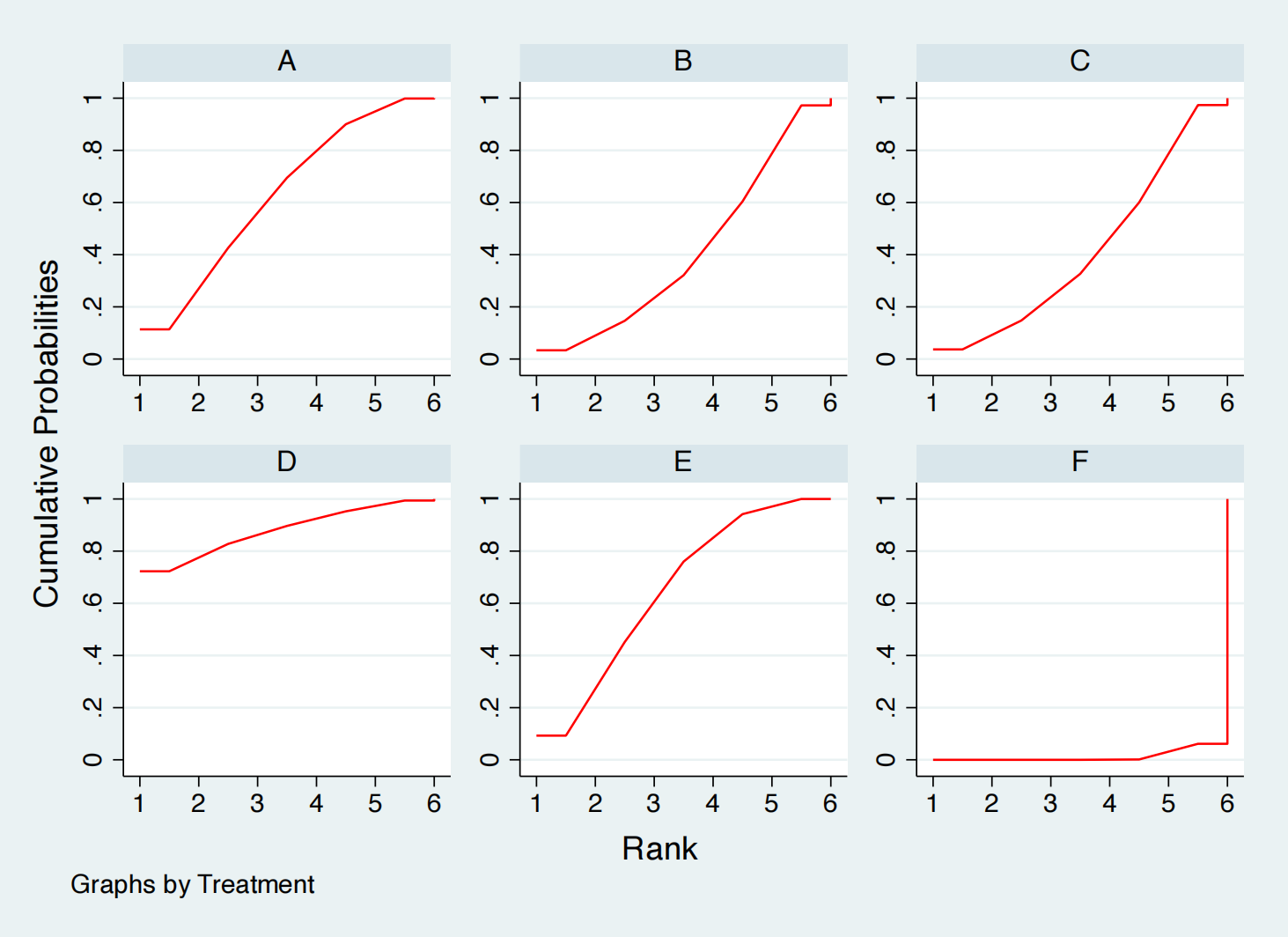


**Abbreviations:**SUCRA, surface under the cumulative ranking curve; A, Atorvastatin 10 mg+ST; B, Atorvastatin 20mg+ST; C, Rosuvastatin 10mg+ST; D, Rosuvastatin 20mg+ST; E, Simvastatin 20mg+ST; F, ST.

**Table S11.3**: SUCRA of the effects of various Stains on **6MWD**

| **Treatment** | **SUCRA** | **PrBest** | **MeanRank** |
| --- | --- | --- | --- |
| Atorvastatin 10 mg+ST | 62.7 | 11.4 | 2.9 |
| Atorvastatin 20mg+ST | 41.6 | 3.4 | 3.9 |
| Rosuvastatin 10mg+ST | 41.7 | 3.7 | 3.9 |
| Rosuvastatin 20mg+ST | 87.9 | 72.3 | 1.6 |
| Simvastatin 20mg+ST | 64.9 | 9.3 | 2.8 |
| ST | 1.3 | 0 | 5.9 |

**Figure S11.4**: Cumulative ranking curve plots of Stains for **FVC** in range network. Higher surface under the curve reflects higher probability of association with **FVC**.


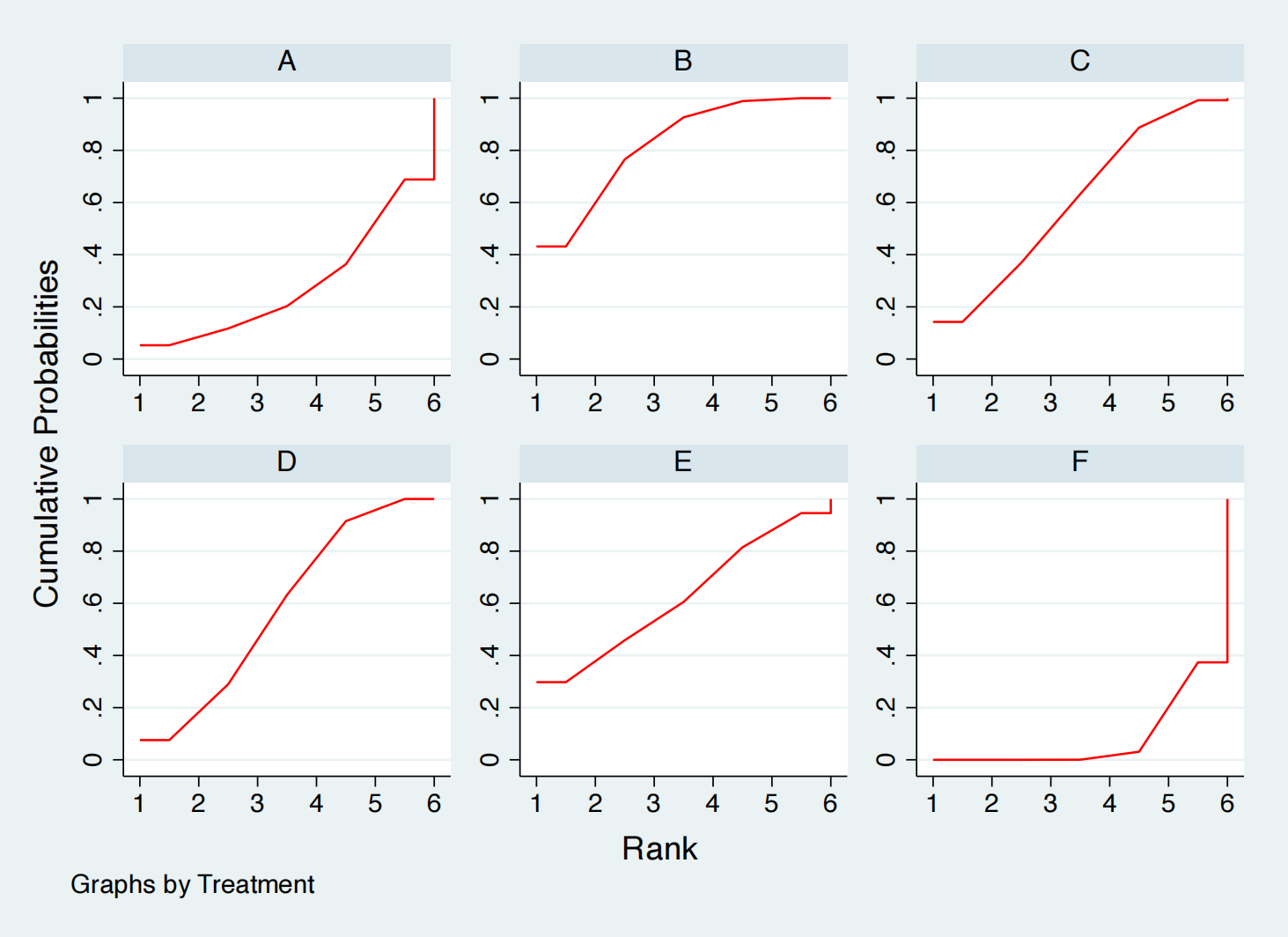


**Abbreviations:**SUCRA, surface under the cumulative ranking curve; A, Atorvastatin 10 mg+ST; B, Atorvastatin 20mg+ST; C, Rosuvastatin 10mg+ST; D, Simvastatin 20mg+ST; E, Simvastatin 40mg+ST; F, ST.

**Table S11.4**: SUCRA of the effects of various Stains on **FVC**

| **Treatment** | **SUCRA** | **PrBest** | **MeanRank** |
| --- | --- | --- | --- |
| Atorvastatin 10 mg+ST | 28.5 | 5.3 | 4.6 |
| Atorvastatin 20mg+ST | 82.2 | 43.1 | 1.9 |
| Rosuvastatin 10mg+ST | 60.5 | 14.2 | 3 |
| Simvastatin 20mg+ST | 58.2 | 7.6 | 3.1 |
| Simvastatin 40mg+ST | 62.4 | 29.8 | 2.9 |
| ST | 8.1 | 0 | 5.6 |

**Figure S11.5**: Cumulative ranking curve plots of Stains for **FEV1** in range network. Higher surface under the curve reflects higher probability of association with **FEV1**.


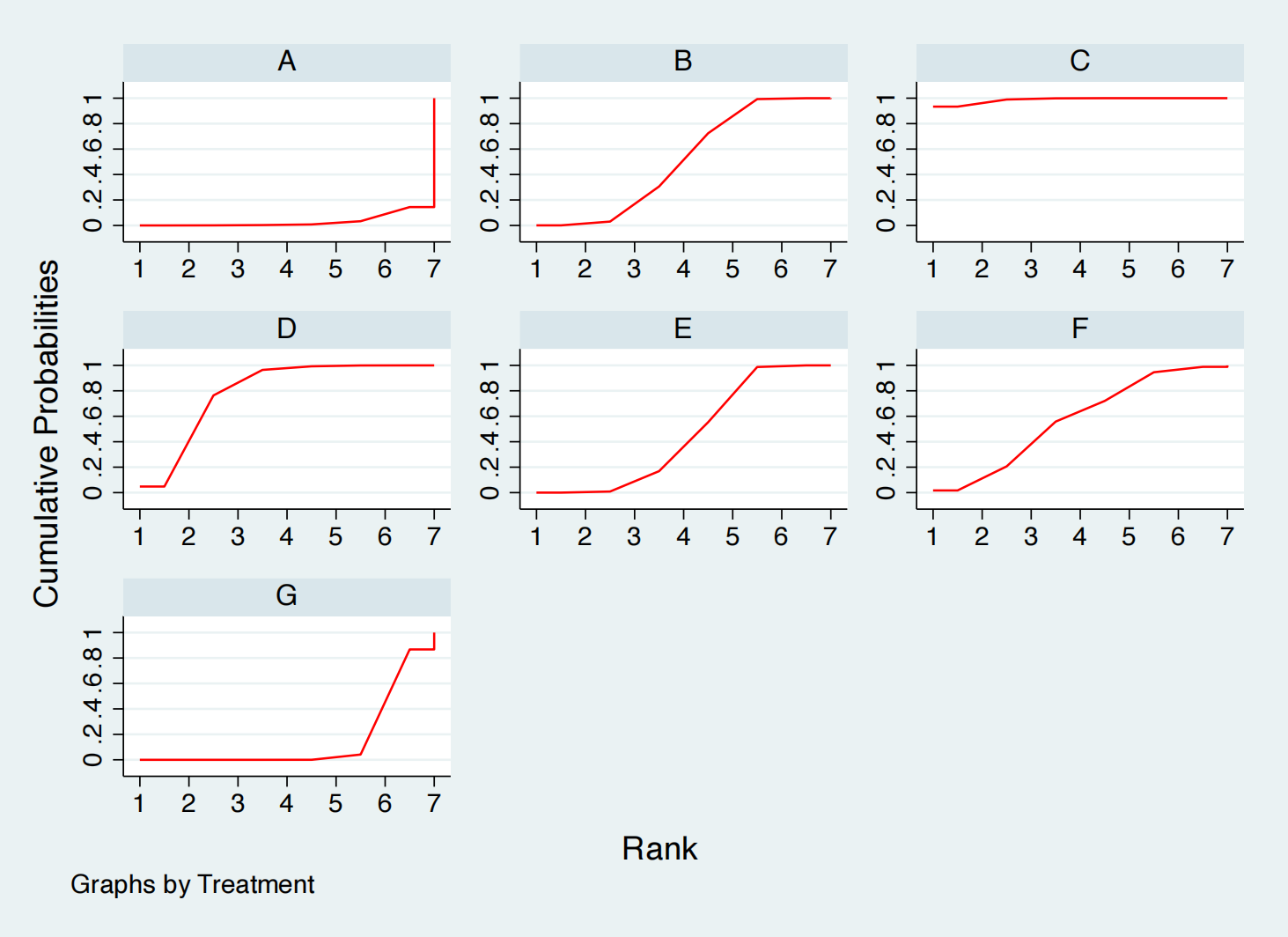


**Abbreviations:**SUCRA, surface under the cumulative ranking curve; A, Atorvastatin 10 mg+ST; B, Atorvastatin 20mg+ST; C, Pravastatin 40mg; D, Rosuvastatin 10mg+ST; E, Simvastatin 20mg+ST; F, Simvastatin 40mg+ST; G, ST.

**Table S11.5**: SUCRA of the effects of various Stains on **FEV1**

| **Treatment** | **SUCRA** | **PrBest** | **MeanRank** |
| --- | --- | --- | --- |
| Atorvastatin 10 mg+ST | 3.2 | 0 | 6.8 |
| Atorvastatin 20mg+ST | 50.9 | 0.1 | 3.9 |
| Pravastatin 40mg | 98.7 | 93.4 | 1.1 |
| Rosuvastatin 10mg+ST | 79.5 | 4.8 | 2.2 |
| Simvastatin 20mg+ST | 45.3 | 0 | 4.3 |
| Simvastatin 40mg+ST | 57.3 | 1.7 | 3.6 |
| ST | 15.2 | 0 | 6.1 |

**Figure S11.6**: Cumulative ranking curve plots of Stains for **FEV1/FVC** in range network. Higher surface under the curve reflects higher probability of association with **FEV1/FVC**.


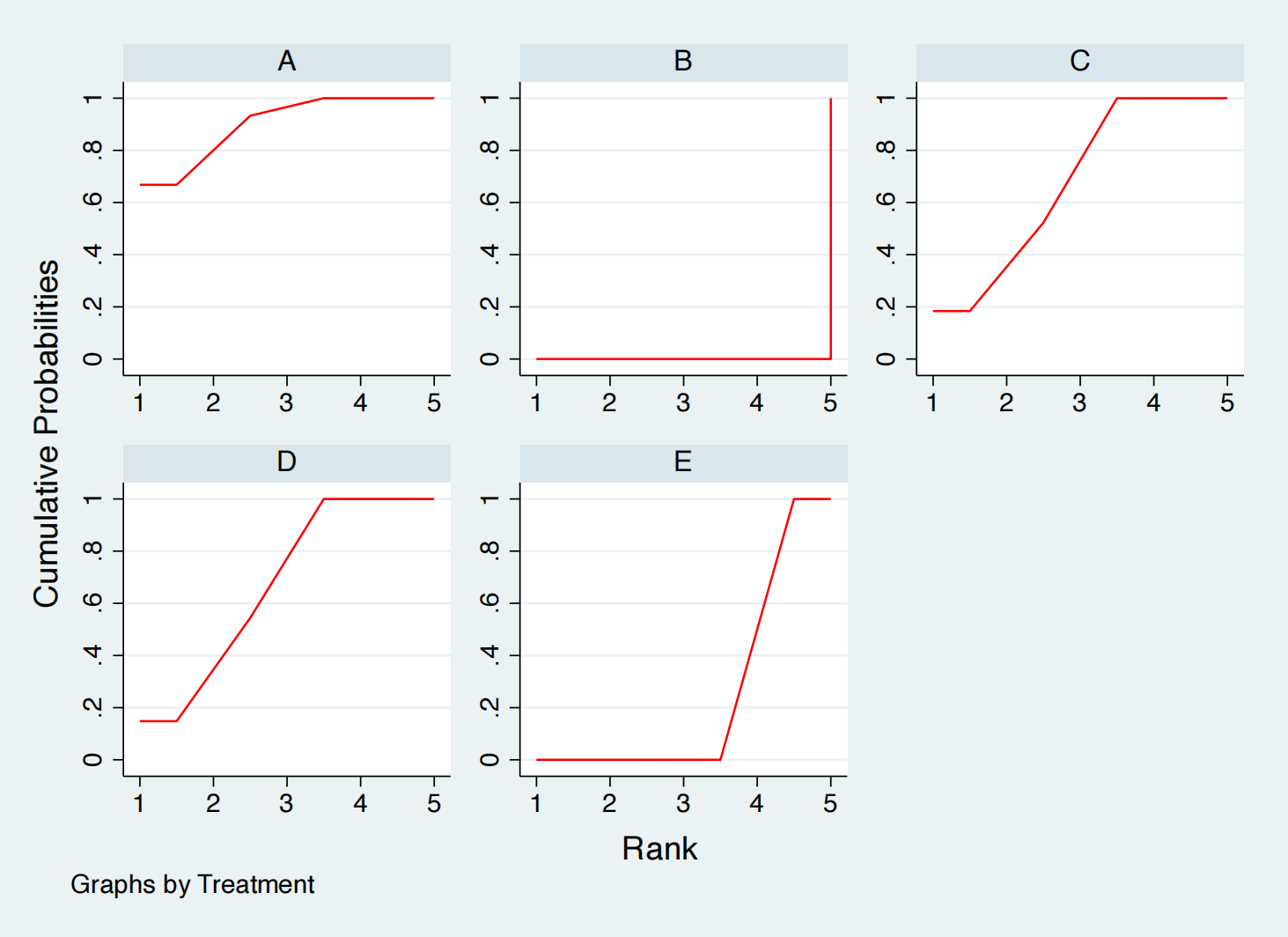


**Abbreviations:**SUCRA, surface under the cumulative ranking curve; A, Atorvastatin 10 mg+ST; B, Fluvastatin 40mg+ST; C, Rosuvastatin 10mg+ST; D, Simvastatin 20mg+ST; E, ST.

**Table S11.6**: SUCRA of the effects of various Stains on **FEV1/FVC**

| **Treatment** | **SUCRA** | **PrBest** | **MeanRank** |
| --- | --- | --- | --- |
| Atorvastatin 20mg+ST | 90 | 66.8 | 1.4 |
| Fluvastatin 40mg+ST | 0 | 0 | 5 |
| Rosuvastatin 10mg+ST | 67.7 | 18.4 | 2.3 |
| Simvastatin 20mg+ST | 67.3 | 14.8 | 2.3 |
| ST | 25 | 0 | 4 |

**Figure S11.7**: Cumulative ranking curve plots of Stains for **PO2** in range network. Higher surface under the curve reflects higher probability of association with **PO2**.

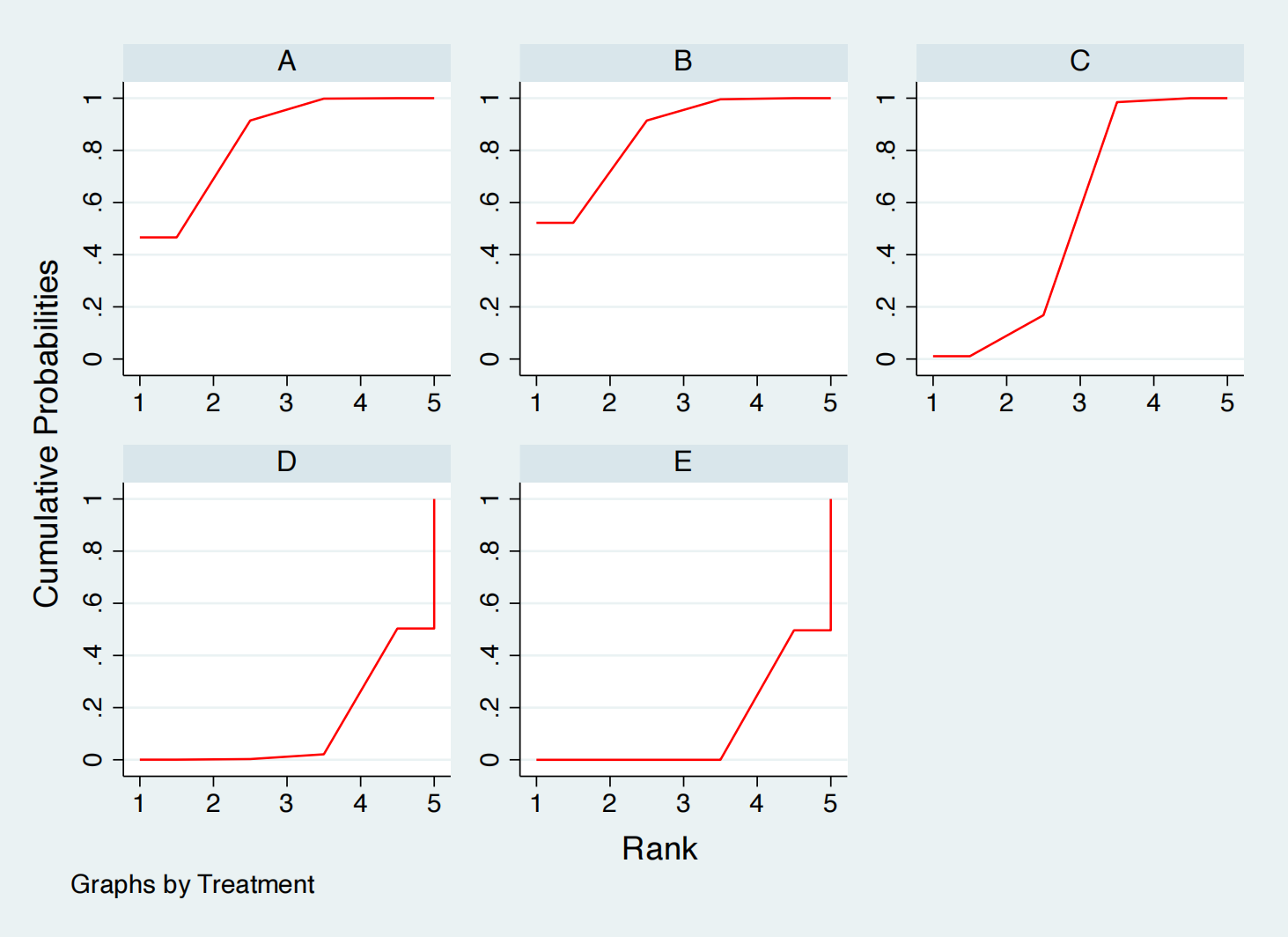


**Abbreviations:**SUCRA, surface under the cumulative ranking curve; A, Atorvastatin 10 mg+ST; B, Atorvastatin 20mg+ST; C, Simvastatin 20mg+ST; D, Simvastatin 40mg+ST; E, ST.

**Table S11.7**: SUCRA of the effects of various Stains on **PO2**

| **Treatment** | **SUCRA** | **PrBest** | **MeanRank** |
| --- | --- | --- | --- |
| Atorvastatin 10 mg+ST | 84.5 | 46.6 | 1.6 |
| Atorvastatin 20mg+ST | 85.8 | 52.2 | 1.6 |
| Simvastatin 20mg+ST | 54.1 | 1.1 | 2.8 |
| Simvastatin 40mg+ST | 13.2 | 0.1 | 4.5 |
| ST | 12.4 | 0 | 4.5 |

**Figure S11.8**: Cumulative ranking curve plots of Stains for **PCO2** in range network. Higher surface under the curve reflects higher probability of association with **PCO2**.


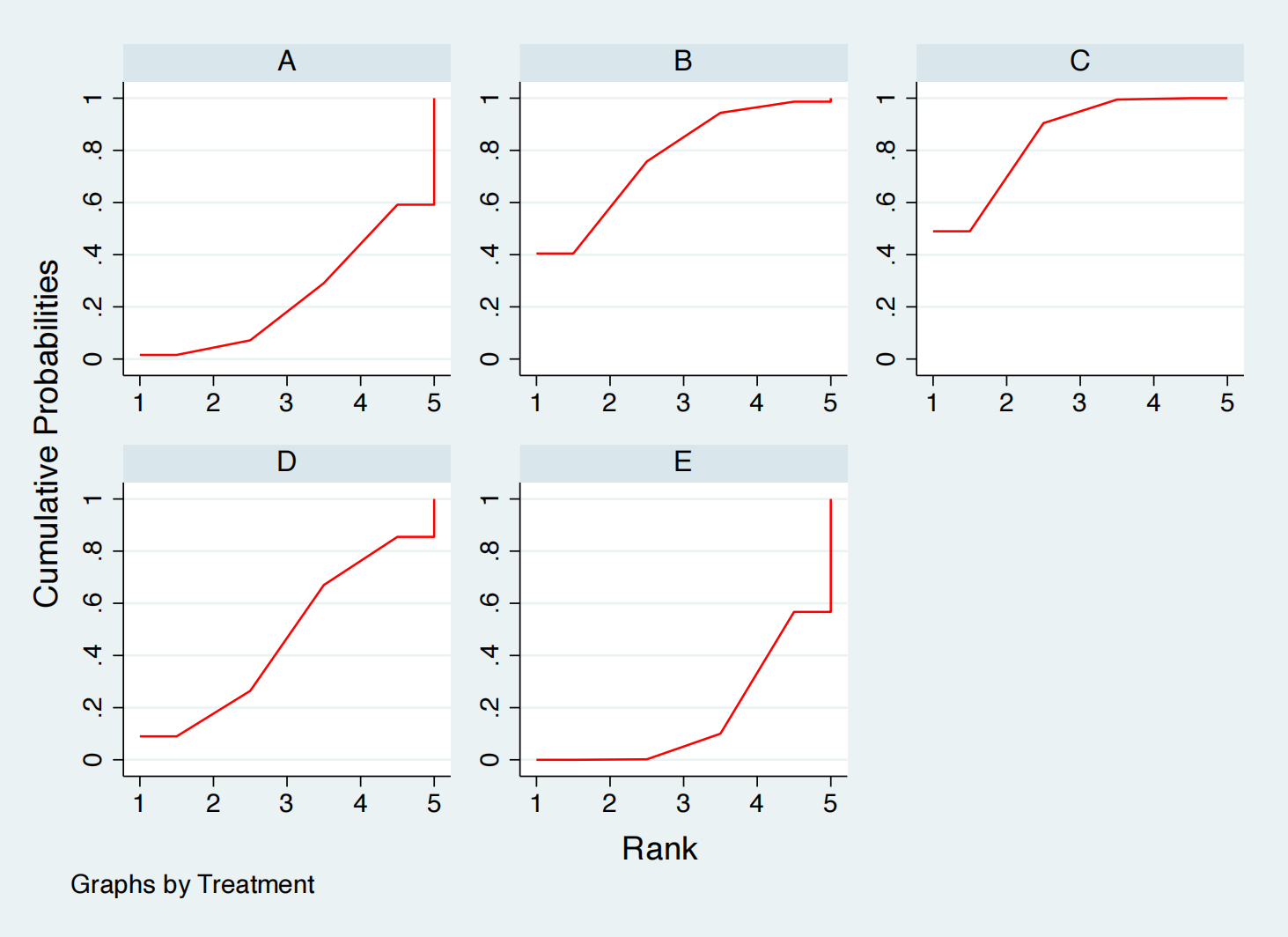


**Abbreviations:**SUCRA, surface under the cumulative ranking curve; A, Atorvastatin 10 mg+ST; B, Atorvastatin 20mg+ST; C, Simvastatin 20mg+ST; D, Simvastatin 40mg+ST; E, ST.

**Table S11.8**: SUCRA of the effects of various Stains on **PCO2**

| **Treatment** | **SUCRA** | **PrBest** | **MeanRank** |
| --- | --- | --- | --- |
| Atorvastatin 10 mg+ST | 24.3 | 1.6 | 4 |
| Atorvastatin 20mg+ST | 77.3 | 40.4 | 1.9 |
| Simvastatin 20mg+ST | 84.7 | 49 | 1.6 |
| Simvastatin 40mg+ST | 47 | 9 | 3.1 |
| ST | 16.7 | 0 | 4.3 |

**Figure S11.9**: Cumulative ranking curve plots of Stains for **TNF-α** in range network. Higher surface under the curve reflects higher probability of association with **TNF-α**.


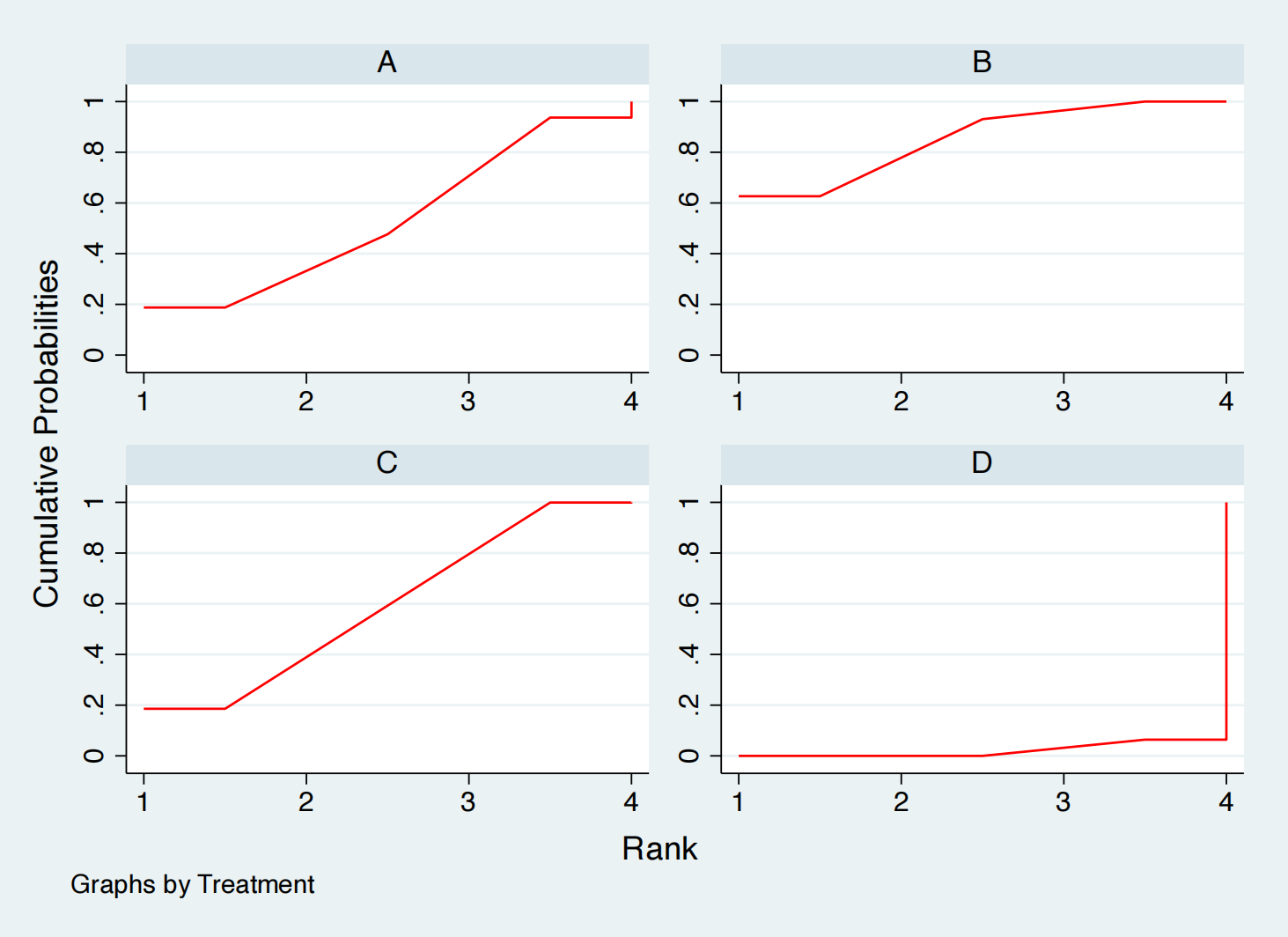


**Abbreviations:**SUCRA, surface under the cumulative ranking curve; A, Atorvastatin 10 mg+ST; B, Atorvastatin 20mg+ST; C, Simvastatin 20mg+ST; D, ST.

**Table S11.9**: SUCRA of the effects of various Stains on **TNF-α**

| **Treatment** | **SUCRA** | **PrBest** | **MeanRank** |
| --- | --- | --- | --- |
| Atorvastatin 10mg+ST | 53.4 | 18.8 | 2.4 |
| Atorvastatin 20mg+ST | 85.2 | 62.7 | 1.4 |
| Simvastatin 20mg+ST | 59.3 | 18.6 | 2.2 |
| ST | 2.1 | 0 | 3.9 |

**Figure S11.10**: Cumulative ranking curve plots of Stains for **hs-CRP** in range network. Higher surface under the curve reflects higher probability of association with **hs-CRP**.


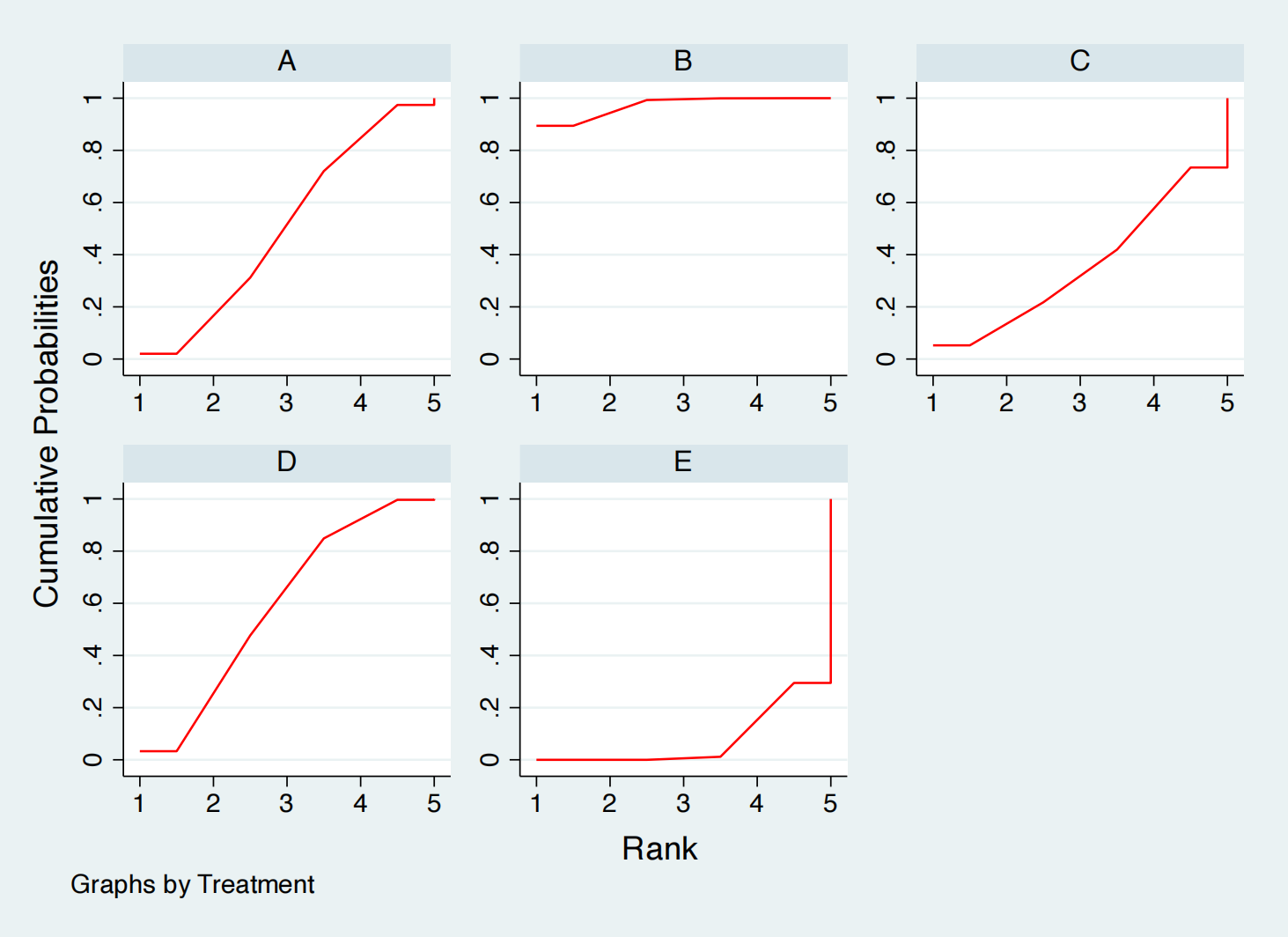


**Abbreviations:**SUCRA, surface under the cumulative ranking curve; A, Atorvastatin 10 mg+ST; B, Atorvastatin 20mg+ST; C, Fluvastatin 40mg+ST;D, Simvastatin 20mg+ST; E, ST.

**Table S11.10**: SUCRA of the effects of various Stains on **hs-CRP**

| **Treatment** | **SUCRA** | **PrBest** | **MeanRank** |
| --- | --- | --- | --- |
| Atorvastatin 10mg+ST | 50.7 | 2 | 3 |
| Atorvastatin 20mg+ST | 97.2 | 89.4 | 1.1 |
| Fluvastatin 40mg+ST | 35.6 | 5.3 | 3.6 |
| Simvastatin 20mg+ST | 58.9 | 3.3 | 2.6 |
| ST | 7.7 | 0 | 4.7 |

**Figure S11.11**: Cumulative ranking curve plots of Stains for **IL-6** in range network. Higher surface under the curve reflects higher probability of association with **IL-6**.


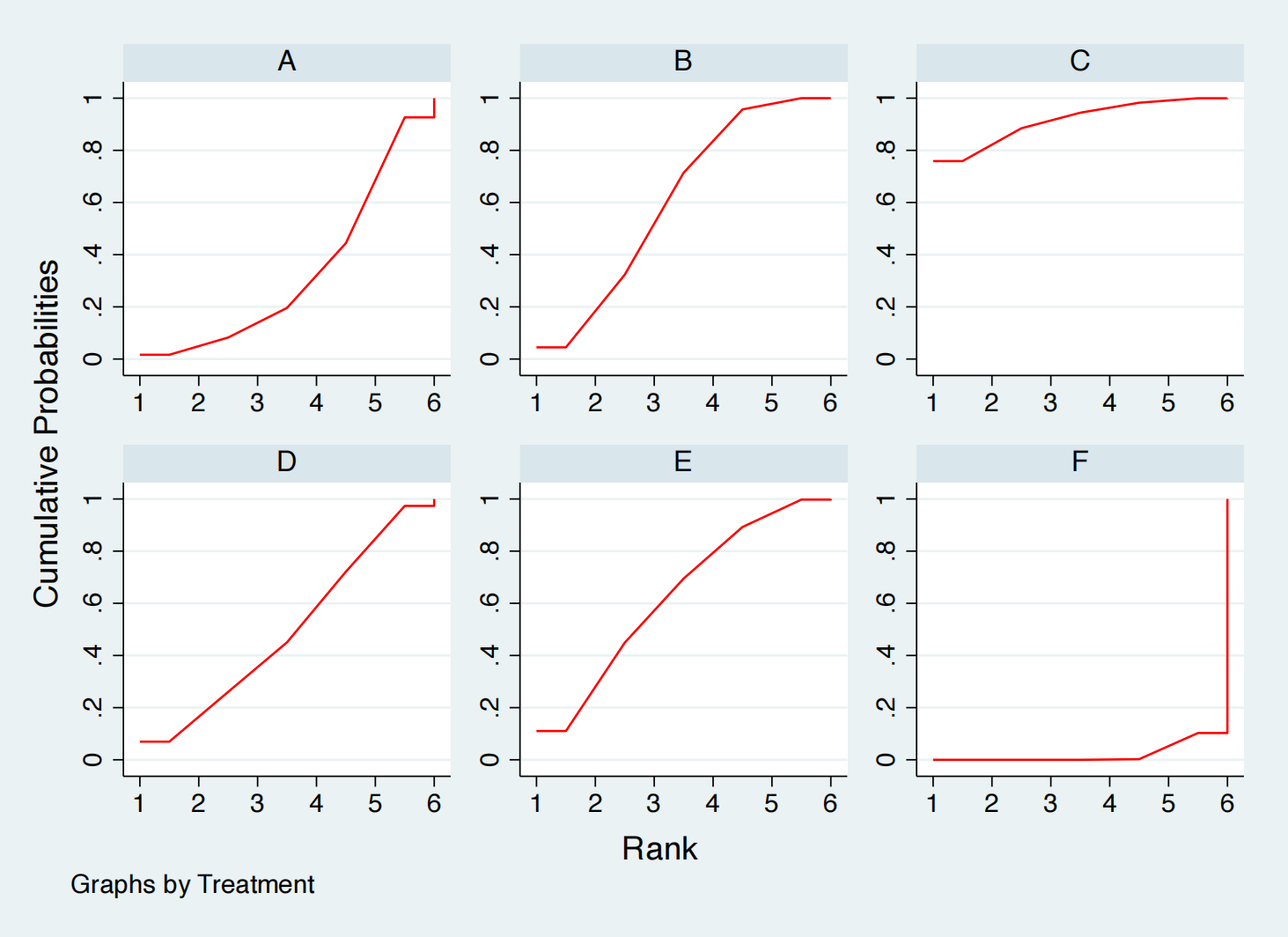


**Abbreviations:**SUCRA, surface under the cumulative ranking curve; A, Atorvastatin 10 mg+ST; B, Atorvastatin 20mg+ST; C, Rosuvastatin 10mg+ST; D, Rosuvastatin 20mg+ST; E, Simvastatin 20mg+ST; F, ST.

**Table S11.11**: SUCRA of the effects of various Stains on **IL-6**

| **Treatment** | **SUCRA** | **PrBest** | **MeanRank** |
| --- | --- | --- | --- |
| Atorvastatin 10mg+ST | 33.3 | 1.6 | 4.3 |
| Atorvastatin 20mg+ST | 60.8 | 4.5 | 3 |
| Rosuvastatin 10mg+ST | 91.4 | 75.9 | 1.4 |
| Rosuvastatin 20mg+ST | 49.5 | 7 | 3.5 |
| Simvastatin 20mg+ST | 62.9 | 11 | 2.9 |
| ST | 2.1 | 0 | 5.9 |

**Figure S11.12**: Cumulative ranking curve plots of Stains for **NO** in range network. Higher surface under the curve reflects higher probability of association with **NO**.


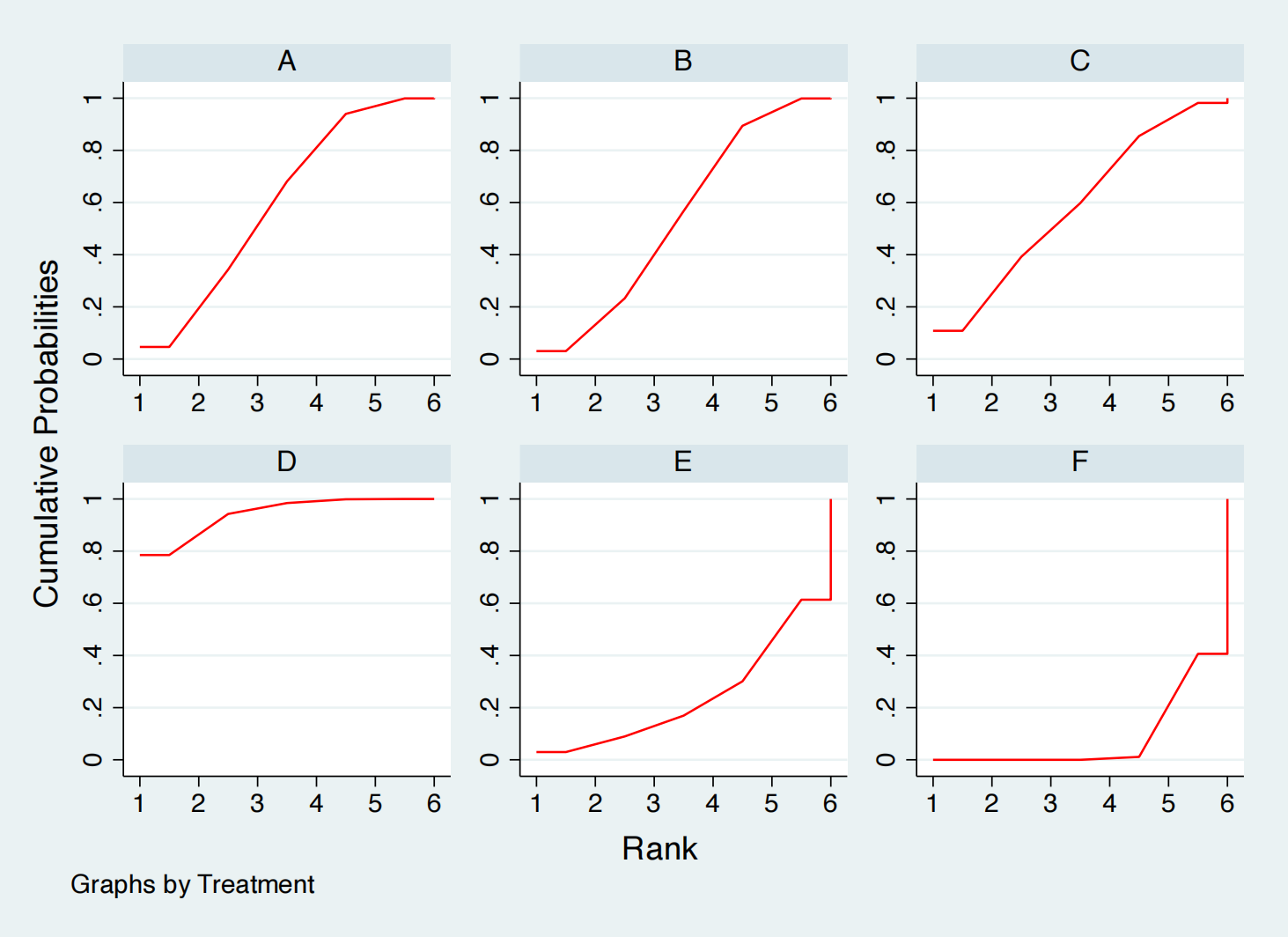


**Abbreviations:**SUCRA, surface under the cumulative ranking curve; A, Atorvastatin 10 mg+ST; B, Atorvastatin 20mg+ST; C, Fluvastatin 40mg+ST; D, Simvastatin 20mg+ST; E, Simvastatin 40mg+ST; F, ST.

**Table S11.12**: SUCRA of the effects of various Stains on **NO**

| **Treatment** | **SUCRA** | **PrBest** | **MeanRank** |
| --- | --- | --- | --- |
| Atorvastatin 10mg+ST | 60.2 | 4.6 | 3 |
| Atorvastatin 20mg+ST | 54.5 | 3 | 3.3 |
| Fluvastatin 40mg+ST | 58.7 | 10.8 | 3.1 |
| Simvastatin 20mg+ST | 94.2 | 78.5 | 1.3 |
| Simvastatin 40mg+ST | 24.1 | 3 | 4.8 |
| ST | 8.4 | 0 | 5.6 |

**Figure S11.13**: Cumulative ranking curve plots of Stains for ET-1 in range network. Higher surface under the curve reflects higher probability of association with ET-1.


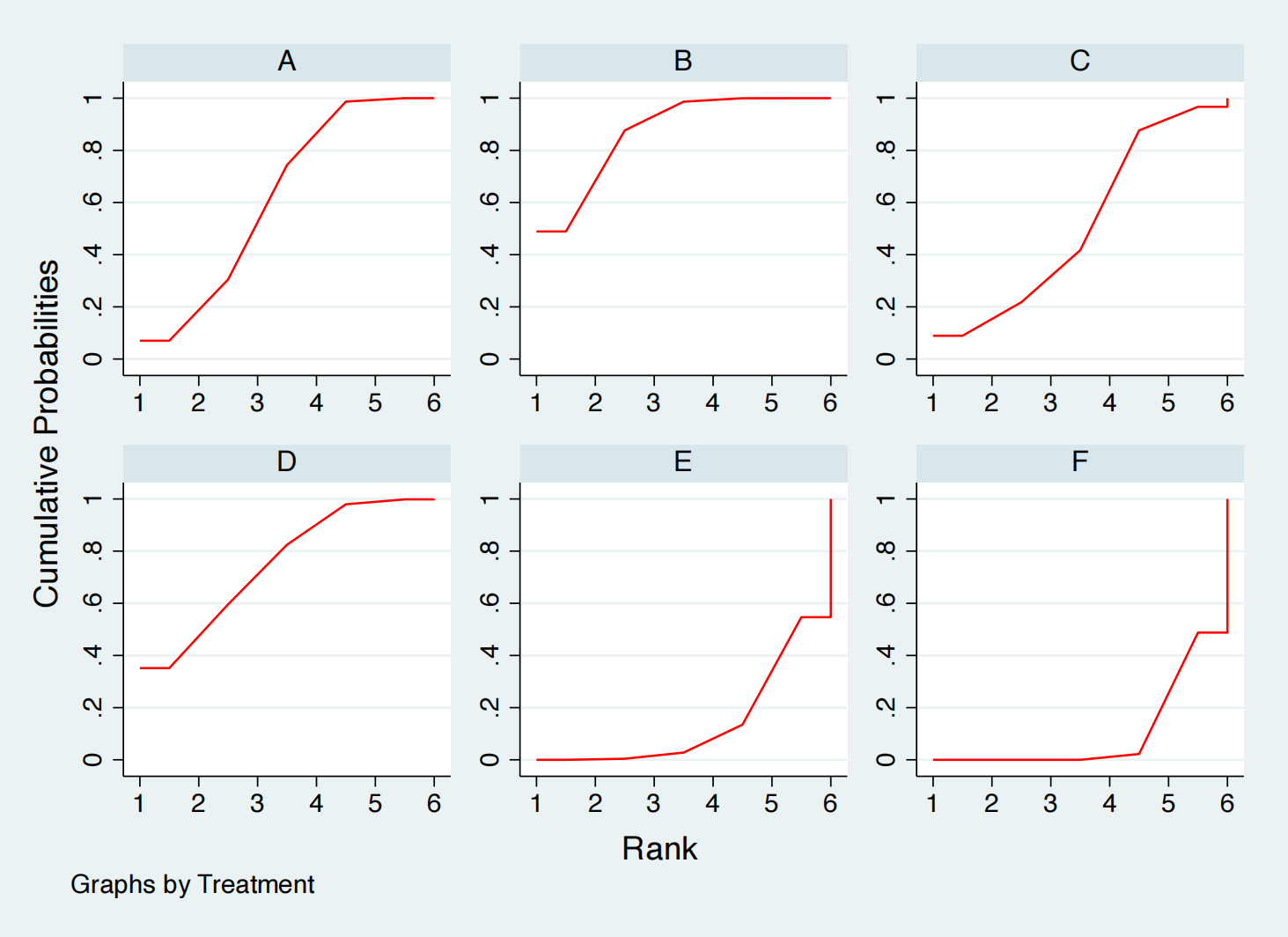


**Abbreviations:**SUCRA, surface under the cumulative ranking curve; A, Atorvastatin 10 mg+ST; B, Atorvastatin 20mg+ST; C, Fluvastatin 40mg+ST; D, Simvastatin 20mg+ST; E, Simvastatin 40mg+ST; F, ST.

**Table S11.13**: SUCRA of the effects of various Stains on ET-1

| **Treatment** | **SUCRA** | **PrBest** | **MeanRank** |
| --- | --- | --- | --- |
| Atorvastatin 10mg+ST | 62.1 | 7 | 2.9 |
| Atorvastatin 20mg+ST | 87 | 48.9 | 1.6 |
| Fluvastatin 40mg+ST | 51.4 | 8.9 | 3.4 |
| Simvastatin 20mg+ST | 75 | 35.2 | 2.2 |
| Simvastatin 40mg+ST | 14.3 | 0 | 5.3 |
| ST | 10.2 | 0 | 5.5 |

**Appendix 12: league table of Summary Estimates for Stains on PH-COPD Derived from Network Meta-analysis of 41 Trials**

**Table S12.1: sPAP**

The columns represent the comparison of the row drug class to the column drug class. The rows represent the comparison of the row drug class to the column drug class. The effect estimates are expressed as MD and 95% CI. For example, the MD in sPAP for Atorvastatin 10 mg+ST compared to Atorvastatin 20mg+ST is -2.96 (95% CI -6.76, 0.89). MD＞0 favors the drug in the row, and MD＜0 favors the drug in the column. According to the CINeMA, the certainty of the evidence for our comparisons of interest is included in the league table, with * as low, † as moderate, and ‡ as high confidence in the evidence. The CINeMA assessment is shown in Appendix 8.

| Atorvastatin 10 mg+ST |  |  |  |  |  |  |  |  |  |
| --- | --- | --- | --- | --- | --- | --- | --- | --- | --- |
| -2.96  (-6.76, 0.89) | Atorvastatin 20mg+ST |  |  |  |  |  |  |  |  |
| -4.5  (-12.07, 3.09) | -1.53  (-8.64, 5.54) | Atorvastatin 40mg+ST |  |  |  |  |  |  |  |
| -3.14  (-8.17, 1.95) | -0.18  (-4.45, 4.09) | 1.36  (-6.49, 9.2) | Fluvastatin 40mg+ST |  |  |  |  |  |  |
| -2.21  (-8.85, 4.48) | 0.76  (-5.36, 6.86) | 2.28  (-6.66, 11.23) | 0.93  (-6.06, 7.89) | Pravastatin 40mg |  |  |  |  |  |
| 0.59  (-3.83, 4.99) | 3.55 *  (0.03, 7.02) | 5.09  (-2.37, 12.5) | 3.73  (-1.13, 8.51) | 2.81  (-3.75, 9.23) | Rosuvastatin 10mg+ST |  |  |  |  |
| -4.21  (-10.77, 2.36) | -1.25  (-7.25, 4.71) | 0.27  (-8.57, 9.15) | -1.08  (-7.89, 5.77) | -2.01  (-10.1, 6.11) | -4.81  (-11.14, 1.62) | Rosuvastatin 20mg+ST |  |  |  |
| -2.99  (-6.79, 0.83) | -0.03  (-2.73, 2.63) | 1.51  (-5.57, 8.58) | 0.14  (-4.15, 4.4) | -0.79  (-6.9, 5.29) | -3.58 *  (-7.03, -0.09) | 1.22  (-4.76, 7.17) | Simvastatin 20mg+ST |  |  |
| -5.35  (-9.72, -0.91) | -2.38  (-5.85, 1.09) | -0.84  (-8.27, 6.54) | -2.21  (-7.03, 2.61) | -3.15  (-9.6, 3.35) | -5.94 *  (-10.02, -1.78) | -1.13  (-7.49, 5.23) | -2.36  (-5.79, 1.12) | Simvastatin 40mg+ST |  |
| -8.21 ‡  (-11.49, -4.87) | -5.25 †  (-7.17, -3.33) | -3.71  (-10.54, 3.12) | -5.07 †  (-8.91, -1.23) | -6.01 *  (-11.81, -0.21) | -8.8 ‡  (-11.68, -5.85) | -3.99  (-9.65, 1.67) | -5.22 †  (-7.08, -3.33) | -2.86  (-5.76, 0.02) | ST |

**Table S12.2: mPAP**

The columns represent the comparison of the row drug class to the column drug class. The rows represent the comparison of the row drug class to the column drug class. The effect estimates are expressed as MD and 95% CI. For example, the MD in mPAP for Atorvastatin 20mg+ST compared to Rosuvastatin 20mg+ST is 0.95 (95% CI -9.96, 11.74). MD＞0 favors the drug in the row, and MD＜0 favors the drug in the column.

| Atorvastatin 20mg+ST |  |  |  |  |
| --- | --- | --- | --- | --- |
| 0.95 (-9.96, 11.74) | Rosuvastatin 20mg+ST |  |  |  |
| -1.72 (-10.41, 7.18) | -2.66 (-11.26, 6.16) | Simvastatin 20mg+ST |  |  |
| -1.46 (-11.89, 8.97) | -2.41 (-12.79, 8.06) | 0.27 (-8.11, 8.5) | Simvastatin 40mg+ST |  |
| -5.06 (-12.7, 2.65) | -6 (-13.57, 1.65) | -3.33 (-7.7, 0.88) | -3.59 (-10.72, 3.55) | ST |

**Table S12.3: 6MWD**

The columns represent the comparison of the row drug class to the column drug class. The rows represent the comparison of the row drug class to the column drug class. The effect estimates are expressed as MD and 95% CI. For example, the MD in 6MWD for Atorvastatin 10 mg+ST compared to Atorvastatin 20mg+ST is 11.74 (95% CI -39.91, 65.2). MD＜0 favors the drug in the row, and MD＞0 favors the drug in the column.

| Atorvastatin 10 mg+ST |  |  |  |  |  |
| --- | --- | --- | --- | --- | --- |
| 11.74 (-39.91, 65.2) | Atorvastatin 20mg+ST |  |  |  |  |
| 12.18 (-38.74, 66.24) | 0.53 (-55.13, 57.83) | Rosuvastatin 10mg+ST |  |  |  |
| -25.99 (-96.76, 48.39) | -37.75 (-111.72, 38.78) | -38.3 (-113.29, 37.21) | Rosuvastatin 20mg+ST |  |  |
| -1.03 (-43.19, 45.34) | -12.84 (-60.36, 37.42) | -13.33 (-61.44, 36.01) | 24.99 (-45.09, 95.32) | Simvastatin 20mg+ST |  |
| 41 (8.75, 76.85) | 29.2 (-9.87, 70.15) | 28.74 (-10.87, 68.92) | 67.03 (2.77, 130.86) | 42.08 (13.43, 69.97) | ST |

**Table S12.4: FVC**

The columns represent the comparison of the row drug class to the column drug class. The rows represent the comparison of the row drug class to the column drug class. The effect estimates are expressed as MD and 95% CI. For example, the MD in FVC for Atorvastatin 10 mg+ST compared to Atorvastatin 20mg+ST is -0.3 (95% CI -0.8, 0.21). MD＜0 favors the drug in the row, and MD＞0 favors the drug in the column.

| Atorvastatin 10 mg+ST |  |  |  |  |  |
| --- | --- | --- | --- | --- | --- |
| -0.3 (-0.8, 0.21) | Atorvastatin 20mg+ST |  |  |  |  |
| -0.19 (-0.75, 0.36) | 0.1 (-0.25, 0.45) | Rosuvastatin 10mg+ST |  |  |  |
| -0.19 (-0.7, 0.32) | 0.11 (-0.16, 0.37) | 0.01 (-0.34, 0.36) | Simvastatin 20mg+ST |  |  |
| -0.21 (-0.88, 0.45) | 0.09 (-0.42, 0.59) | -0.02 (-0.57, 0.54) | -0.02 (-0.53, 0.49) | Simvastatin 40mg+ST |  |
| 0.1 (-0.37, 0.57) | 0.4 (0.21, 0.58) | 0.29 (0, 0.59) | 0.29 (0.1, 0.48) | 0.31 (-0.16, 0.78) | ST |

**Table S12.5: FEV1**

The columns represent the comparison of the row drug class to the column drug class. The rows represent the comparison of the row drug class to the column drug class. The effect estimates are expressed as MD and 95% CI. For example, the MD in FEV1 for Atorvastatin 10 mg+ST compared to Atorvastatin 20mg+ST is -0.3 (95% CI -0.8, 0.21). MD＜0 favors the drug in the row, and MD＞0 favors the drug in the column.

| Atorvastatin 10 mg+ST |  |  |  |  |  |  |
| --- | --- | --- | --- | --- | --- | --- |
| -0.3 (-0.62, -0.01) | Atorvastatin 20mg+ST |  |  |  |  |  |
| -0.69 (-1.1, -0.29) | -0.39 (-0.7, -0.06) | Pravastatin 40mg |  |  |  |  |
| -0.46 (-0.78, -0.13) | -0.15 (-0.35, 0.06) | 0.23 (-0.1, 0.57) | Rosuvastatin 10mg+ST |  |  |  |
| -0.28 (-0.58, 0.02) | 0.02 (-0.14, 0.19) | 0.41 (0.09, 0.72) | 0.18 (-0.03, 0.38) | Simvastatin 20mg+ST |  |  |
| -0.34 (-0.74, 0.06) | -0.04 (-0.34, 0.28) | 0.35 (-0.06, 0.76) | 0.12 (-0.21, 0.45) | -0.06 (-0.36, 0.25) | Simvastatin 40mg+ST |  |
| -0.13 (-0.41, 0.15) | 0.17 (0.06, 0.3) | 0.56 (0.27, 0.85) | 0.33 (0.16, 0.5) | 0.15 (0.04, 0.26) | 0.21 (-0.08, 0.5) | ST |

**Table S12.6: FEV1/FVC**

The columns represent the comparison of the row drug class to the column drug class. The rows represent the comparison of the row drug class to the column drug class. The effect estimates are expressed as MD and 95% CI. For example, the MD in FEV1/FVC for Atorvastatin 20 mg+ST compared to Fluvastatin 40mg+ST is 15.66 (95% CI 9.42, 22.15). MD＜0 favors the drug in the row, and MD＞0 favors the drug in the column.

| Atorvastatin 20mg+ST |  |  |  |  |
| --- | --- | --- | --- | --- |
| 15.66 (9.42, 22.15) | Fluvastatin 40mg+ST |  |  |  |
| 1.38 (-2.9, 6.02) | -14.26 (-21.18, -7.29) | Rosuvastatin 10mg+ST |  |  |
| 1.36 (-2.57, 5.31) | -14.3 (-21.08, -7.76) | -0.01 (-5.05, 4.66) | Simvastatin 20mg+ST |  |
| 6.29 (4.01, 8.87) | -9.36 (-15.22, -3.49) | 4.91 (1.13, 8.61) | 4.93 (1.98, 8.17) | ST |

**Table S12.7: PO2**

The columns represent the comparison of the row drug class to the column drug class. The rows represent the comparison of the row drug class to the column drug class. The effect estimates are expressed as MD and 95% CI. For example, the MD in PO2 for Atorvastatin 20 mg+ST compared to Fluvastatin 40mg+ST is 15.66 (95% CI 9.42, 22.15). MD＜0 favors the drug in the row, and MD＞0 favors the drug in the column.

| Atorvastatin 10 mg+ST |  |  |  |  |
| --- | --- | --- | --- | --- |
| -0.22 (-12.81, 12.28) | Atorvastatin 20mg+ST |  |  |  |
| 4.08 (-5.66, 13.63) | 4.29 (-5.52, 13.98) | Simvastatin 20mg+ST |  |  |
| 11.59 (-1.54, 24.57) | 11.79 (-1.26, 24.89) | 7.51 (-2.66, 17.93) | Simvastatin 40mg+ST |  |
| 11.6 (2.78, 20.43) | 11.81 (2.93, 20.78) | 7.51 (3.71, 11.49) | 0 (-9.55, 9.52) | ST |

**Table S12.8: PCO2**

The columns represent the comparison of the row drug class to the column drug class. The rows represent the comparison of the row drug class to the column drug class. The effect estimates are expressed as MD and 95% CI. For example, the MD in PCO2 for Atorvastatin 10 mg+ST compared to Atorvastatin 20mg+ST is 8.32 (95% CI 11.72, 28.51). MD＞0 favors the drug in the row, and MD＜0 favors the drug in the column.

| Atorvastatin 10 mg+ST |  |  |  |  |
| --- | --- | --- | --- | --- |
| 8.32 (-11.72, 28.51) | Atorvastatin 20mg+ST |  |  |  |
| 9.19 (-6.85, 25.03) | 0.85 (-15.02, 16.82) | Simvastatin 20mg+ST |  |  |
| 3.57 (-16.87, 24.12) | -4.78 (-25.06, 15.72) | -5.63 (-21.8, 10.7) | Simvastatin 40mg+ST |  |
| -0.4 (-14.63, 13.91) | -8.76 (-22.92, 5.47) | -9.59 (-16.65, -2.5) | -3.96 (-18.66, 10.58) | ST |

**Table S12.9: TNF-α**

The columns represent the comparison of the row drug class to the column drug class. The rows represent the comparison of the row drug class to the column drug class. The effect estimates are expressed as MD and 95% CI. For example, the MD in TNF-αfor Atorvastatin 10 mg+ST compared to Atorvastatin 20mg+ST is 3.44 (95% CI -8.92, 15.77). MD＞0 favors the drug in the row, and MD＜0 favors the drug in the column.

| Atorvastatin 10mg+ST |  |  |  |
| --- | --- | --- | --- |
| 3.44 (-8.92, 15.77) | Atorvastatin 20mg+ST |  |  |
| 0.82 (-13.27, 14.97) | -2.62 (-12.4, 7.28) | Simvastatin 20mg+ST |  |
| -6.82 (-19.05, 5.56) | -10.25 (-17.24, -3.14) | -7.64 (-14.47, -0.75) | ST |

**Table S12.10: hs-CRP**

The columns represent the comparison of the row drug class to the column drug class. The rows represent the comparison of the row drug class to the column drug class. The effect estimates are expressed as MD and 95% CI. For example, the MD in hs-CRP for Atorvastatin 10 mg+ST compared to Atorvastatin 20mg+ST is 3.4 (95% CI -0.3, 7.24). MD＞0 favors the drug in the row, and MD＜0 favors the drug in the column.

| Atorvastatin 10mg+ST |  |  |  |  |
| --- | --- | --- | --- | --- |
| 3.4 (-0.3, 7.24) | Atorvastatin 20mg+ST |  |  |  |
| -1.13 (-8.01, 5.68) | -4.53 (-10.88, 1.67) | Fluvastatin 40mg+ST |  |  |
| 0.57 (-3.83, 4.99) | -2.83 (-6.32, 0.58) | 1.69 (-4.84, 8.31) | Simvastatin 20mg+ST |  |
| -2.72 (-6.14, 0.69) | -6.12 (-8.22, -4.15) | -1.58 (-7.51, 4.33) | -3.28 (-6.14, -0.53) | ST |

**Table S12.11: IL-6**

The columns represent the comparison of the row drug class to the column drug class. The rows represent the comparison of the row drug class to the column drug class. The effect estimates are expressed as MD and 95% CI. For example, the MD in IL-6 for Atorvastatin 10 mg+ST compared to Atorvastatin 20mg+ST is 4.02 (95% CI -7.28, 15.11). MD＞0 favors the drug in the row, and MD＜0 favors the drug in the column.

| Atorvastatin 10mg+ST |  |  |  |  |  |
| --- | --- | --- | --- | --- | --- |
| 4.02 (-7.28, 15.11) | Atorvastatin 20mg+ST |  |  |  |  |
| 10.64 (-6.9, 27.94) | 6.64 (-7.71, 20.87) | Rosuvastatin 10mg+ST |  |  |  |
| 2.53 (-14.38, 19.21) | -1.48 (-14.99, 11.98) | -8.14 (-26.22, 10.11) | Rosuvastatin 20mg+ST |  |  |
| 4.67 (-9.43, 20.08) | 0.67 (-9.53, 12.21) | -5.93 (-21.54, 11.03) | 2.15 (-12.68, 18.45) | Simvastatin 20mg+ST |  |
| -5.75 (-17.12, 5.39) | -9.77 (-15.08, -4.44) | -16.41 (-29.64, -3.04) | -8.28 (-20.69, 4.16) | -10.44 (-20.65, -1.67) | ST |

**Table S12.12: NO**

The columns represent the comparison of the row drug class to the column drug class. The rows represent the comparison of the row drug class to the column drug class. The effect estimates are expressed as MD and 95% CI. For example, the MD in NO for Atorvastatin 10 mg+ST compared to Atorvastatin 20mg+ST is 0.44 (95% CI -5.78, 6.66). MD＜0 favors the drug in the row, and MD＞0 favors the drug in the column.

| Atorvastatin 10mg+ST |  |  |  |  |  |
| --- | --- | --- | --- | --- | --- |
| 0.44 (-5.78, 6.66) | Atorvastatin 20mg+ST |  |  |  |  |
| 0.09 (-8.54, 8.75) | -0.35 (-8.99, 8.26) | Fluvastatin 40mg+ST |  |  |  |
| -3.44 (-9.6, 3.08) | -3.89 (-10.11, 2.7) | -3.52 (-12.13, 5.36) | Simvastatin 20mg+ST |  |  |
| 3.84 (-5.92, 13.65) | 3.39 (-6.44, 13.28) | 3.76 (-7.76, 15.21) | 7.26 (-2.78, 17.02) | Simvastatin 40mg+ST |  |
| 4.98 (0.64, 9.37) | 4.54 (0.14, 9.01) | 4.89 (-2.54, 12.36) | 8.42 (3.66, 12.86) | 1.16 (-7.64, 9.9) | ST |

**Table S12.13: ET-1**

The columns represent the comparison of the row drug class to the column drug class. The rows represent the comparison of the row drug class to the column drug class. The effect estimates are expressed as MD and 95% CI. For example, the MD in ET-1 for Atorvastatin 10 mg+ST compared to Atorvastatin 20mg+ST is 2.38 (95% CI -2.92, 7.64). MD＞0 favors the drug in the row, and MD＜0 favors the drug in the column.

| Atorvastatin 10mg+ST |  |  |  |  |  |
| --- | --- | --- | --- | --- | --- |
| 2.38 (-2.92, 7.64) | Atorvastatin 20mg+ST |  |  |  |  |
| -1.74 (-11.38, 7.84) | -4.14 (-13.11, 4.84) | Fluvastatin 40mg+ST |  |  |  |
| 1.37 (-8.13, 10.93) | -1.01 (-9.89, 7.95) | 3.13 (-8.68, 14.89) | Simvastatin 20mg+ST |  |  |
| -7.11 (-16.36, 2.22) | -9.5 (-18.07, -0.82) | -5.35 (-16.85, 6.29) | -8.47 (-20.07, 3.1) | Simvastatin 40mg+ST |  |
| -7.44 (-12.14, -2.73) | -9.82 (-13.03, -6.6) | -5.68 (-14.03, 2.7) | -8.8 (-17.13, -0.5) | -0.33 (-8.35, 7.68) | ST |

**Appendix 13:** **Sensitivity analyses of sPAP.**

The term "before" refers to the combined mead difference (MD) from all studies directly compared to ST, which are -5.57[-6.72, -4.43]. We employed a leave-one-out analysis to investigate whether the exclusion of each study significantly affected the original effect size. For example, in the comparison with ST, after excluding “Yu 2012”, the remaining effect size is -5.36 [-6.51, -4.22].

**
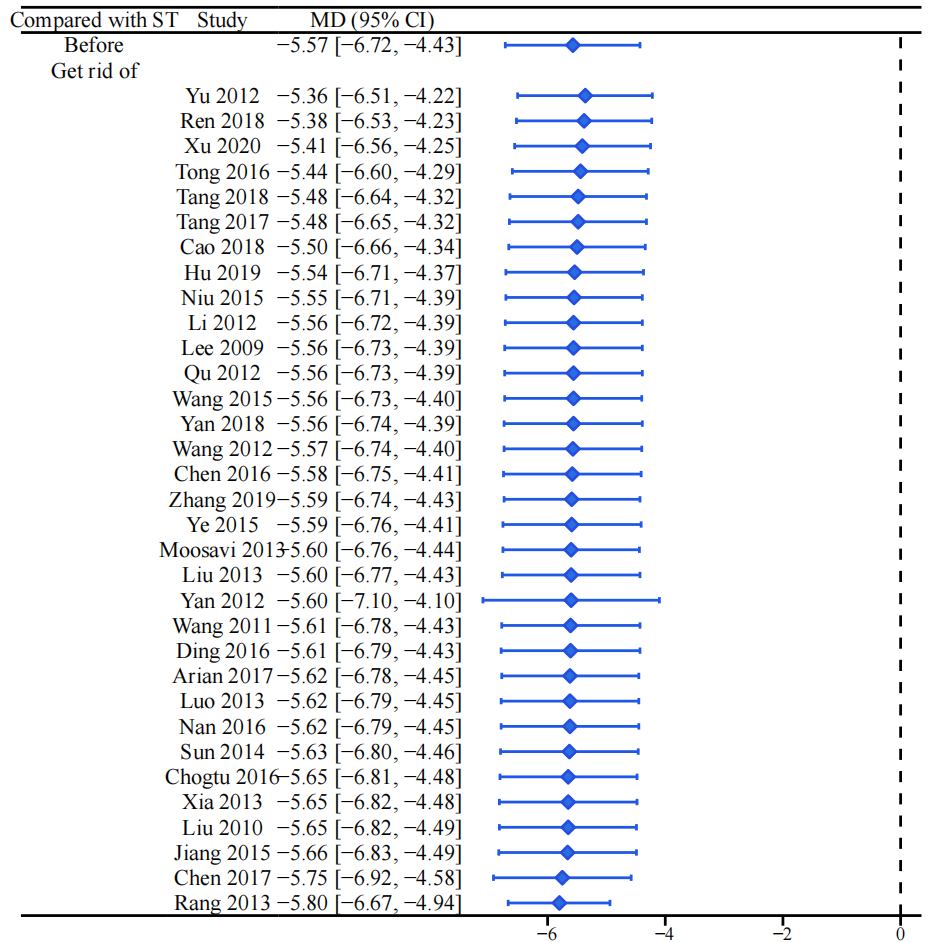
**

**Appendix 14: The meta-regression of the factors that may lead to differences to the main outcome indicators**

| **Factors** | **sPAP** | | | | |
| --- | --- | --- | --- | --- | --- |
|  | **Coefficient** | **std err** | **t** | **P value** | **95%CI** |
| **sPAP** | 0.0209 | 0.247 | 0.072 | 0.982 | -0.828, 0.848 |
| **COPD stage** | -0.5318 | 0.252 | -1.853 | 0.116 | -2.016, 0.236 |
| **Age** | -0.0106 | 0.182 | -0.046 | 0.919 | -0.391, 0.279 |

**Appendix 15: Summary of Treatment Effects and CINeMA Confidence Ratings for Primary Outcomes**

| Compared to ST | ①sPAP | ②mPAP | ③6WMD | ④FVC | ⑤FEV1 | ⑥FEV1 FVC | ⑦PO2 | ⑧PCO2 | CiNeMA |
| --- | --- | --- | --- | --- | --- | --- | --- | --- | --- |
| Atorvastatin 10 mg+ST | -**8.21 (-11.49, -4.87)** | -5.06 (-12.7, 2.65) | **41 (8.75, 76.85)** | 0.1 (-0.37, 0.57) | -0.13 (-0.41, 0.15) | NA | **11.6 (2.78, 20.43)** | -0.4 (-14.63, 13.91) | High |
| Atorvastatin 20mg+ST | **-5.25 (-7.17, -3.33)** | NA | 29.2 (-9.87, 70.15) | **0.4 (0.21, 0.58)** | **0.17 (0.06, 0.3)** | **6.29 (4.01, 8.87)** | **11.81 (2.93, 20.78)** | -8.76 (-22.92, 5.47) | Moderate |
| Atorvastatin 40mg+ST | -3.71 (-10.54, 3.12) | NA | NA | NA | NA | NA | NA | NA | Low |
| Fluvastatin 40mg+ST | **-5.07 (-8.91, -1.23)** | NA | NA | NA | NA | **-9.36 (-15.22, -3.49)** | NA | NA | Moderate |
| Pravastatin 40mg | **-6.01 (-11.81, -0.21)** | NA | NA | NA | **0.56 (0.27, 0.85)** | NA | NA | NA | Low |
| Rosuvastatin 10mg+ST | **-8.8 (-11.68, -5.85)** | NA | 28.74 (-10.87, 68.92) | 0.29 (0, 0.59) | **0.33 (0.16, 0.5)** | **4.91 (1.13, 8.61)** | NA | NA | High |
| Rosuvastatin 20mg+ST | -3.99 (-9.65, 1.67) | -6 (-13.57, 1.65) | **67.03 (2.77, 130.86)** | NA | NA | NA | NA | NA | Low |
| Simvastatin 20mg+ST | **-5.22 (-7.08, -3.33)** | -3.33 (-7.7, 0.88) | **42.08 (13.43, 69.97)** | **0.29 (0.1, 0.48)** | **0.15 (0.04, 0.26)** | **4.93 (1.98, 8.17)** | **7.51 (3.71, 11.49)** | **-9.59 (-16.65, -2.5)** | Moderate |
| Simvastatin 40mg+ST | -2.86 (-5.76, 0.02) | -3.59 (-10.72, 3.55) | NA | 0.31 (-0.16, 0.78) | 0.21 (-0.08, 0.5) | NA | 0 (-9.55, 9.52) | -3.96 (-18.66, 10.58) | Low |

**Appendix 16: R Codes and Final Dataset Used for Analysis**

# Step 1: Install packages

install.packages("gemtc")

install.packages("readxl")

# Step 2: Load packages

library(gemtc)

library(readxl)

# Step 3: Import data (adjust file path accordingly; continuous data)

file_path <- "/Users/zhoujingchao/Desktop/Workbook1.xlsx"

data <- read_excel(file_path)

# Step 4: Build network meta-analysis data structure

network <- mtc.network(data)

# Step 5: Plot network diagram

plot(network)

# Step 6: Build model (default MD; likelihood and link for continuous outcomes)

model <- mtc.model(network, type = "consistency", n.chain = 4, likelihood = "normal", link = "identity", linearModel = "random")

# Step 7: Run MCMC

result <- mtc.run(model, n.adapt = 20000, n.iter = 50000, thin = 1)

# Step 8: Summarize results

summary(result)

# Calculate heterogeneity tau²

sd <- 1.0895

tau_squared <- sd^2

print(tau_squared)

# Step 9: Forest plot (all vs a specific reference)

forest(relative.effect(result, t1 = "7"), use.description = TRUE)

# Forest plot (specific interventions vs control group)

forest(relative.effect(result, t1 = "M", c("A", "B", "C", "D")), use.description = TRUE)

# Step 10: Assess MCMC convergence

gelman.plot(result)

plot(result)

# Step 11: Generate SUCRA ranking results

ranks <- rank.probability(result, preferredDirection = -1)

print(ranks)

plot(ranks)

plot(ranks, beside = TRUE)

# Step 12: Calculate SUCRA values

sucra <- function(ranks) {

apply(ranks, 1, function(p) {

a <- length(p)

sum(cumsum(p[-a]) / (a - 1))

})

}

sucra_values <- sucra(ranks)

print(sucra_values)

# Step 13: Create league table

setwd("/Users/zhoujingchao/Desktop/")

a <- round(relative.effect.table(result), 2)

print(a)

write.csv(a, "/Users/zhoujingchao/Desktop/leaguetable.csv")

# Step 14: Build UME (Unrelated Mean Effects) model

modelume <- suppressWarnings(mtc.model(network, type = "ume", n.chain = 4, likelihood = "normal", link = "identity", linearModel = "random"))

# Step 15: Run MCMC for UME model

resultume <- mtc.run(modelume, n.adapt = 20000, n.iter = 50000, thin = 1)

# Step 16: Summarize UME model results

summary(resultume)

# Step 17: Node-splitting method for local inconsistency

resultnodesplit <- mtc.nodesplit(network)

# Step 18: Summarize node-splitting results

b <- summary(resultnodesplit)

print(b)

# Step 19: Plot node-splitting results

plot(b)

# Save long figure

tiff("1.tiff", height = 10000, width = 4000, res = 600)

plot(b)

dev.off()

# Step 20: Heterogeneity analysis

resultanohe <- mtc.anohe(network)

c <- summary(resultanohe)

print(c)

plot(c)

# Save long figure

tiff("2.tiff", height = 20000, width = 5000, res = 600)

plot(c)

dev.off()
